# Dynamic Neural Deactivation Bridges Direct and Competitive Inhibition Processes

**DOI:** 10.1002/advs.202506833

**Published:** 2025-08-26

**Authors:** Zhenhong He, Yifan Du, Ziqi Fu, Youcun Zheng, Nils Muhlert, Barbara Sahakian, Rebecca Elliott

**Affiliations:** ^1^ School of Psychology Shenzhen University Shenzhen 518060 China; ^2^ Division of Psychology and Mental Health School of Health Sciences University of Manchester Manchester M13 9PT UK; ^3^ Department of Psychiatry School of Clinical Medicine University of Cambridge Cambridge CB2 0SP UK; ^4^ Department of Psychology Sungkyunkwan University Jongno‐gu 03063 South Korea; ^5^ Department of Psychology McGill University Montreal H3A 1G1 Canada; ^6^ School of Science and Engineering The Chinese University of Hong Kong Shenzhen 518172 China

**Keywords:** inhibition, multimodal neuroimaging, neural deactivation, sensory suppression

## Abstract

Inhibition is an important concept in cognitive neuroscience. Direct inhibition, characterized by the active suppression of stimuli, and competition‐induced inhibition, which involves ignoring irrelevant stimuli by prioritizing relevant ones, have traditionally been considered distinct and studied separately. Although their spatial neural overlap has been highlighted, the temporal dimension—the development of neural activities over time—remains largely unexplored. Using multimodal neuroimaging and behavioral experiments in the auditory and visual domains, in addition to conjunction analyses that capture their neural commonalities, we observed that both inhibition types exhibit a shared deactivation temporal dynamic. It is characterized by a progressive reduction in frontoparietal activation and increased deactivation in sensory regions, a pattern that is positively correlated with improved inhibition performance and whose causal disruption contributes to reduced inhibitory effect. Furthermore, this deactivation‐dominant pattern is consistent across different sensory modalities and generalizes to various low‐processing demand scenarios, whether actively induced or passively experienced. In addition, functional blurring in information clarity during inhibition is found. Overall, the findings reveal that diverse inhibitory processes for modulating information input converge on a shared neural substrate characterized by dynamic feedforward signal attenuation, thereby bridging previously disconnected domains of inhibition research and offering new perspectives of neural deactivation.

## Graphical abstract

Dynamic neural deactivation bridges traditionally distinct inhibitory mechanisms—direct inhibition and competition‐induced inhibition—revealing a common neural signature across modalities. Multimodal neuroimaging and behavioral experiments demonstrate a temporal dynamic characterized by progressive frontoparietal activation decay and enhanced sensory‐region deactivation, crucially linked to inhibition efficacy. This universal neural deactivation mechanism, confirmed through causal manipulation, reshapes understanding of cognitive control processes.

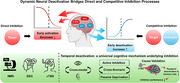

## 1 Introduction

Inhibition cognitive neuroscience. In humans, it plays an important role in the navigation of complex environments by selectively filtering or suppressing irrelevant information.^[^ ^1^, ^2^ ^]^ This critical function is broadly divided into two systems: competition‐induced inhibition, which prioritizes goal‐relevant inputs over distractions (e.g., focusing on a conversation in noise,^[^ ^3^ ^]^ and direct inhibition, which actively blocks the processing of specific stimuli (e.g., suppressing intrusive thoughts).^[^ ^4^ ^]^ Although these systems are functionally distinct—competition‐induced inhibition relies on attentional selection^[^ ^5^, ^6^ ^]^ and direct inhibition depends on explicit suppression^[^ ^4^, ^7^ ^]^—their neural overlaps remain an important open question.

Neuroimaging and clinical studies traditionally dissociate the neural substrates of direct and competition‐induced inhibition. Competition‐induced inhibition primarily activates attentional networks involving posterior cortical regions,^[^ ^8^, ^9^ ^]^ whereas direct inhibition relies on response suppression mechanisms involving the prefrontal‐basal ganglia pathways.^[^ ^1^, ^2^ ^]^ Lesion studies reinforce this distinction: damage to the prefrontal regions can selectively impair direct inhibition, while largely preserving competition‐based processes.^[^ ^10^ ^]^ Meta‐analyses further delineate specialized networks: frontoparietal regions for direct inhibition and posterior hubs for competitive filtering.^[^ ^11^, ^12^ ^]^ Historically, researchers have separately studied these mechanisms, with attention research focusing on competition‐induced inhibition^[^ ^8^ ^]^ and memory/response control paradigms focusing on direct inhibition.^[^ ^1^ ^]^

However, emerging evidence challenges this strict dichotomy. Direct and competition‐induced inhibition both engage overlapping cognitive control mechanisms, such as goal execution and maintenance via the frontoparietal regions,^[^ ^13^ ^]^ and they lead to similar outcomes, including sensory attenuation in modality‐specific cortices.^[^ ^14^, ^15^ ^]^ The identified co‐activation of frontoparietal control networks and sensory deactivation across different inhibition types suggests a shared neural architecture.^[^ ^11^ ^]^ Additional spatial commonalities include shared neural substrates in the right inferior frontal gyrus,^[^ ^1^ ^]^ overlapping activity in the anterior cingulate cortex during conflict monitoring,^[^ ^16^ ^]^ and mutual reliance on basal ganglia structures for implementing motor inhibition.^[^ ^17^ ^]^

However, there remains a fundamental gap in existing knowledge. Although the spatial patterns of neural activation are well‐mapped,^[^ ^11^ ^]^ the temporal dynamics—how neural activity during inhibition unfolds over time—remain unexplored. This oversight is not trivial. Effective interventions (e.g., timing neuromodulation therapies)^[^ ^18^ ^]^ and the development of accurate cognitive models depend on the understanding of the time‐dependent mechanisms underlying inhibition.

Effective inhibition relies on two seemingly opposing processes: activation in control networks to trigger suppression and deactivation in sensory regions to maintain suppression.^[^ ^1^, ^19^ ^]^ However, it remains unclear how both states evolve over time. As inhibitory processes continue, information processing gradually weakens, leading to the everyday experience of stimuli perception without cognitive engagement with the stimuli, e.g., “hearing without listening” or “looking without seeing.”^[^ ^20^, ^21^ ^]^ This pattern suggests a shared neural dynamic underlying various inhibitory processes that similarly reduce information processing. Given the close association between cognitive inhibition and neural deactivation,^[^ ^22^ ^]^ we hypothesized that neural activity across control and sensory networks follows a trajectory toward progressive disengagement during sustained inhibition. We define this disengagement as temporal deactivation, operationally characterized by the reduction in activity of the same brain region from early to late stages. This temporal signature likely serves as the mechanistic connection between direct and competition‐induced inhibition.

**Figure 1:**
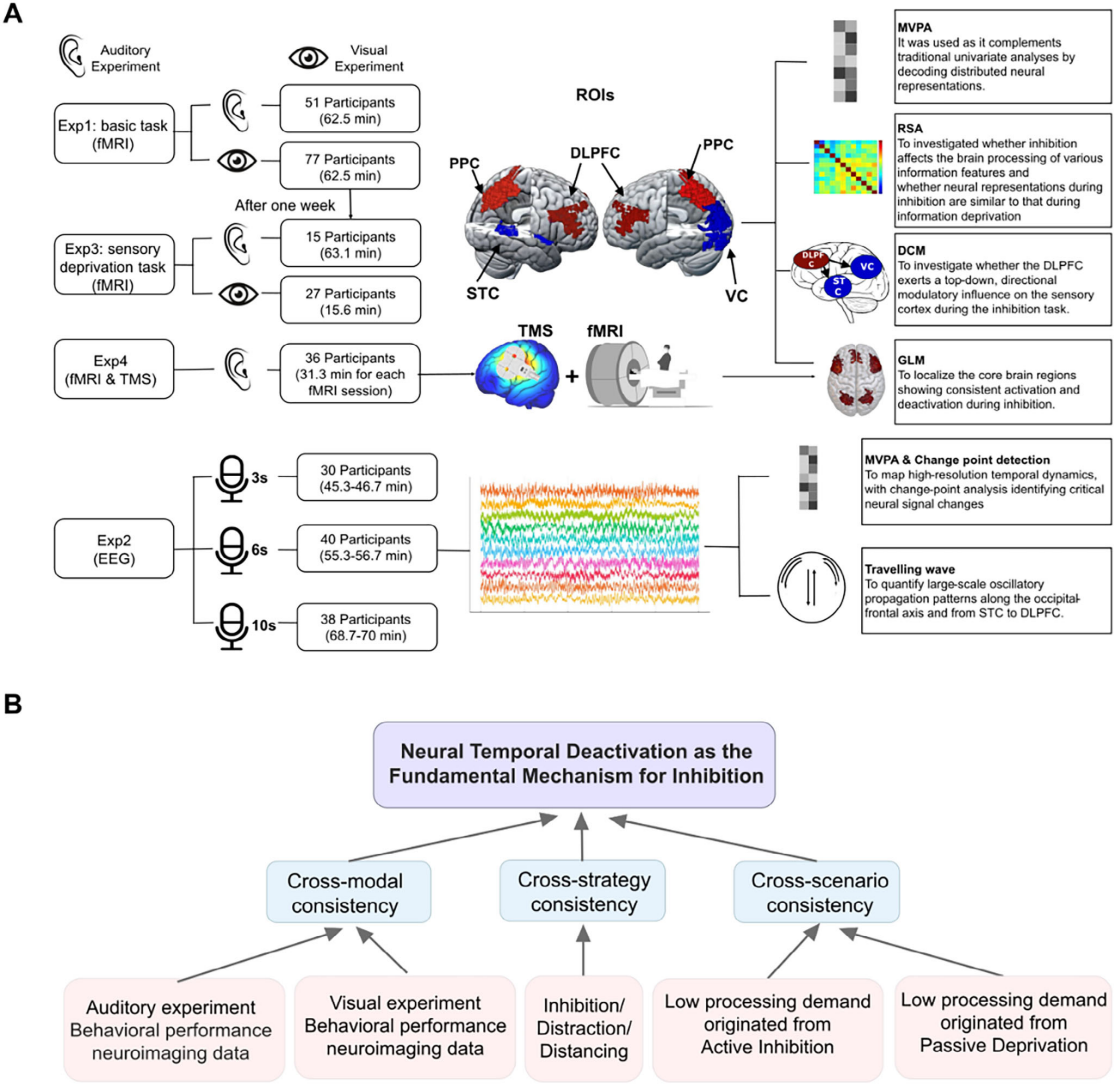
Empirical framework and experimental design of the study. A) Overview of the four experiments conducted to investigate the neural mechanisms of inhibition. Experiment 1 (fMRI) examined cross‐modal inhibition in auditory and visual domains using different participant samples. Experiment 2 (EEG) mapped high‐resolution temporal dynamics across three stimulus durations (3s, 6s, 10s) with independent participant samples. Experiment 3 (fMRI) contrasted active inhibition with concentration and passive deprivation conditions using returning participants from Experiment 1. Experiment 4 (cTBS + fMRI) evaluated the causal role of temporal deactivation (through disrupting DLPFC) in inhibition performance. Multiple analytical approaches used were as follows: region‐of‐interest (ROI) analysis, representational similarity analysis (RSA), DCM, general linear model (GLM), MVPA, change‐point detection, and traveling wave analysis. B) Conceptual framework illustrating how multiple lines of evidence, including cross‐modal consistency (auditory vs visual), cross‐strategy consistency (inhibition/distraction/distancing), and cross‐scenario consistency (low processing demands originated from active inhibition or passive information deprivation), converge to support the hypothesis that neural temporal deactivation serves as a fundamental, universal cognitive mechanism underlying inhibition. The framework demonstrates that diverse inhibitory processes across different modalities, strategies, and contexts all exhibit the same underlying neural signature of progressive deactivation.

To test this hypothesis, we conducted four experiments using multimodal neuroimaging (see **Figure** **1**A; Figure S1, Supporting Information for an overview): Experiment 1 (functional Magnetic Resonance Imaging [fMRI]) examined neural correlates of inhibition across both auditory and visual domains, implementing direct inhibition and competition‐based strategies (distraction and distancing). In addition, dynamic causal modeling (DCM) was used to examine the neural pathways involved in the inhibitory process. In Experiment 2 (Electroencephalogram [EEG]), we mapped high‐resolution temporal dynamics, with change‐point analysis identifying critical neural signal changes and traveling wave analysis^[^ ^23^ ^]^ quantifying large‐scale oscillatory propagation patterns along the occipital‐frontal axis and the temporal‐frontal axis. Experiment 3 (fMRI) contrasted active inhibition with concentration (sustained attention) and passive deprivation (scrambled auditory inputs, eye closure) conditions to dissociate task‐general deactivation patterns. Finally, in Experiment 4 (continuous theta burst stimulation [cTBS] + fMRI), we used a causal intervention to directly test the causal role of temporal deactivation in inhibitory performance, compared to it being merely an epiphenomenal phenomenon. Furthermore, in this experiment, we used cTBS to temporarily disrupt the dorsolateral prefrontal cortex (DLPFC), a key node for inhibitory control, and examined whether this disruption would alter the temporal deactivation dynamic in the sensory cortex and ultimately affect behavioral inhibitory performance. Throughout these experiments, we combined fMRI conjunction analyses^[^ ^24^ ^]^ across strategies with pre‐post change point comparisons to isolate shared mechanisms and their temporal evolution. In addition, multivariate pattern analysis (MVPA)^[^ ^25^ ^]^ was performed as it complements traditional univariate analyses by decoding distributed neural representations.

We identified a consistent temporal deactivation dynamic shared by direct and competition‐induced inhibition, characterized by an early decrease in frontoparietal activation coupled with progressively increasing sensory region deactivation around a critical temporal boundary (≈3s). Traveling wave analysis revealed a sustained suppression of high‐frequency oscillations (beta) alongside alpha‐band amplification during inhibition, which are signatures of a deactivation‐dominant state. This deactivation process coincides with information blurring, where the ability of the brain to associate stimulus features with corresponding neural patterns becomes significantly weakened. Notably, MVPA confirmed these findings, revealing congruent yet complementary evidence through decoding neural states, thus validating distributed representation changes. Providing definitive causal evidence, our cTBS experiment demonstrated that disrupting the DLPFC, the source of top‐down control, directly impaired this temporal deactivation dynamic and consequently weakened inhibitory performance. Furthermore, this temporal deactivation signature appears specific (as states of concentrated attention show the opposite trend) and universal (as passive deprivation produces similar dynamics). Therefore, the observed pure “deactivation‐dominant” temporal dynamic in inhibition does not support deactivation as a secondary consequence of activation, but positions it as the dominant mechanistic endpoint across diverse inhibitory contexts. Figure 1B shows a conceptual framework illustrating how empirical data from multiple dimensions—including cross‐modal, cross‐strategy, and cross‐contextual analyses—converge to support the hypothesis that neural deactivation is a fundamental mechanism for inhibition. This study offers novel insights into the common mechanisms and neural dynamics underlying human inhibitory control.

## 2 Results

### 2.1 Inhibition: A Deactivation‐Dominant Neural Dynamic in Auditory and Visual Modalities

#### 2.1.1 Experimental Design and Behavioral Evidence

**Figure 2:**
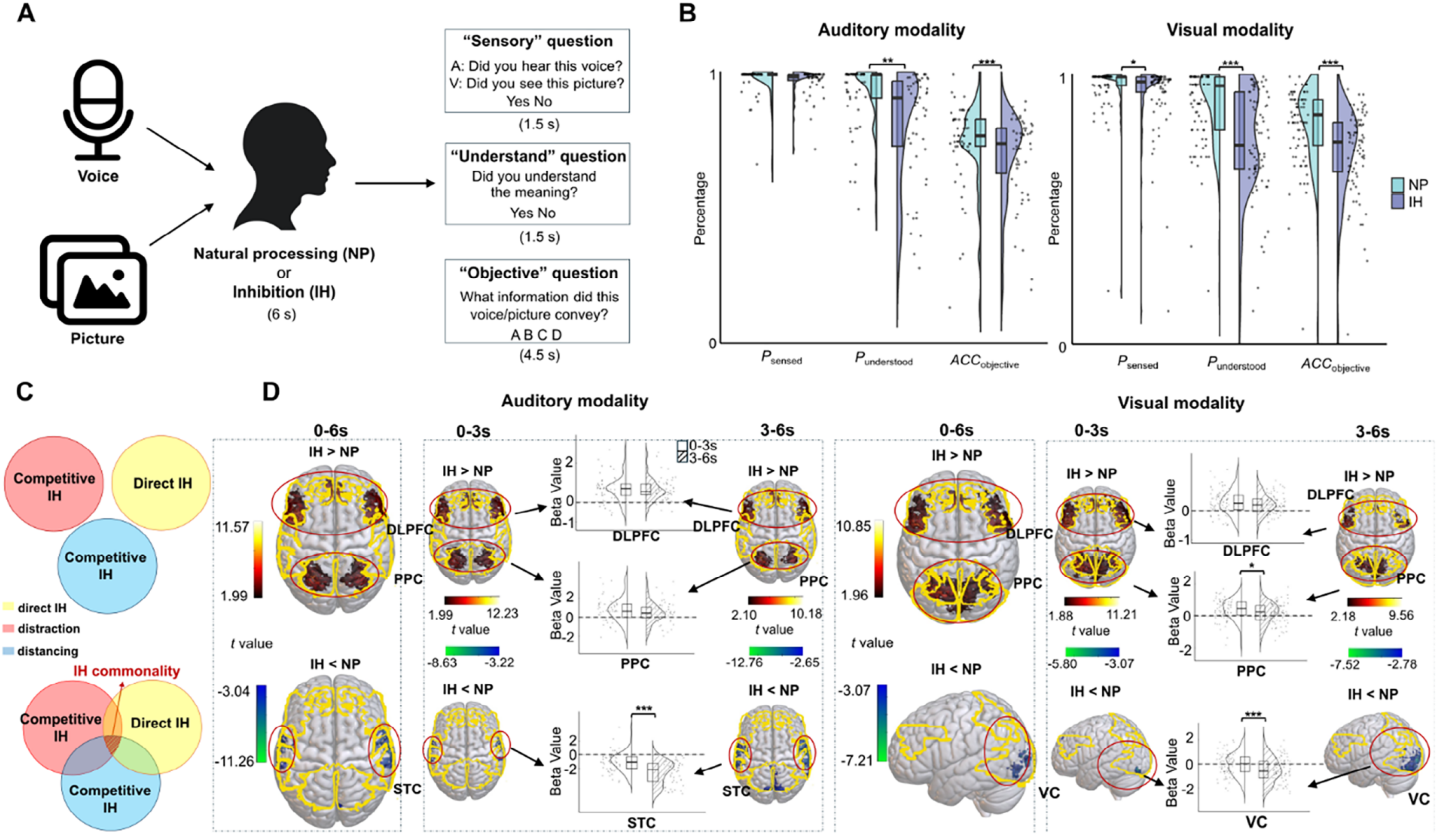
Experimental design and brain responses during inhibition. A) Schematic of auditory and visual inhibition experiments. Participants engaged in either NP or IH of voice or picture stimuli, followed by answering three questions to assess sensory perception, understanding, and the objective information conveyed. B) Behavioral results of auditory (left panel) and visual (right panel) experiments. Raincloud plots represent the distribution of response rates of perceiving (*P_sensed_*) and understanding the stimuli (*P_understood_*), along with accuracy in identifying the conveyed information (*ACC_objective_*). The box plots embedded in the raincloud plots represent the median (center line), interquartile range (boxes; 25–75th percentiles), and whiskers extending to the most extreme data points within 1.5 times the interquartile range. Data beyond this range is considered outliers (same definition applies below). C) Schematic illustrating the logic of conjunction analysis. The upper diagram depicts a scenario where conjunction analysis yields no results, indicating three completely independent inhibitory strategies with no neural overlap. The lower diagram shows the actual findings, where conjunction analysis revealed inhibition—the neural commonalities shared across three inhibitory strategies: two competitive strategies (distraction and distancing) and one direct strategy (inhibition). This overlap demonstrates that these seemingly distinct approaches to cognitive regulation share underlying neural substrates. D) fMRI temporal dynamics of the auditory (left panel) and visual (right panel) experiments. Brain maps show inhibition‐induced activations in the DLPFC, PPC, STC, and VC during early (0–3s) and late ^(3–6s)^ phases. The analysis was conducted at the ROI level. Yellow contours indicate predefined ROI. For the auditory experiment, the ROIs included DLPFC, PPC, and STC, whereas for the visual experiment, the ROIs included DLPFC, PPC, and VC. The color scale indicates t‐values, with the range dynamically set from the minimum to the maximum value among the statistically significant voxels displayed. Raincloud plots show inhibition‐related activation/deactivation (beta values) in each region, segmented into early and late phases. Data were obtained from Experiment 1, involving independent participant samples (Auditory: n = 51; Visual: n = 77). Each data point represents an individual participant (biological replicate). Statistical comparisons (inhibition vs NP conditions, pre‐3s vs post‐3s periods) were performed using paired *t*‐tests on the mean values of each condition. *p(FDR) < 0.05, **p(FDR) < 0.01, ***p(FDR) < 0.001, (two‐sided). All quantitative analyses were conducted using the full anatomical ROI masks, with colored voxels within each mask denoting the regions of maximal statistical significance.

We developed parallel auditory and visual inhibition tasks (Auditory and Visual for short) to investigate the existence, neural representations, and temporal dynamics across sensory modalities. Participants alternated between two conditions: natural processing (NP; passive viewing/listening without explicit control) and inhibition (IH, implemented via direct inhibition, distraction, or distancing strategies). Supporting Information (SI)‐Text 1 provides detailed instructions for implementing cognitive strategies. For each stimulus (voice clips or pictures; see Text S2, Supporting Information for the validation of these experimental materials), participants answered three assessment questions evaluating sensory perception, comprehension, and objective recall (schematic of the trial structure can be seen in **Figure** **2**A; Figure S1A, Supporting Information). Further details are provided in “Experimental Section‐Behavioral task”. Behavioral results confirmed inhibition efficacy: participants exhibited modality‐general reductions in sensory/attentional processing (Visual: *p* < 0.05; Figure 2B), comprehension (*p*‐values  < 0.01, derived from generalized (logistic) linear mixed‐effect models; Figure 2B), and objective identification accuracy (*p*‐values < 0.05; Figure 2B; Figure S1B, Table S1, and Text S3, Supporting Information). These results suggest that inhibition disrupts information propagation, as early sensory‐level disruptions cascaded into downstream cognitive deficits (e.g., impaired comprehension and memory).

#### 2.1.2 Neural Architecture of Cross‐Modal Inhibition (functional Magnetic Resonance Imaging, fMRI)

We first confirmed the strategy‐specific activation patterns among the three individual strategies—namely, direct inhibition, distraction, and distancing. In both auditory and visual experiments, we found that compared to NP, all individual strategies recruited frontoparietal activation (DLPFC / posterior parietal cortex [PPC]) coupled with simultaneous sensory region deactivation (superior temporal cortex [STC[for auditory experiment / visual cortex [VC] for visual experiment; Figure S2, Supporting Information). While the strategies showed some differences in brain activity (Text S4 and Table S2A, Supporting Information), the core pattern of temporal deactivation (post‐3s *β*_(IH‐NP)_‐pre‐3s *β*_(IH‐NP)_) was not significantly different between them in either the visual (*p*(false discovery rate, FDR)‐values ≥ 0.989) or auditory (*p*(FDR)‐values ≥ 0.300) modalities (Table S2b, Supporting Information).

To identify common neural substrates of inhibition across the three strategies, we performed an fMRI conjunction analysis^[^ ^24^ ^]^ across these three conditions (Figure 2C, see “Experimental Section‐Data recording and analysis – MRI data” for method details). This analysis revealed a supra‐strategy control mechanism consistently involving frontoparietal activation (DLPFC / PPC); Figure S3A,B and Tables S3 and S4, Supporting Information) coupled with simultaneous sensory region suppression (STC for auditory experiment / VC for visual experiment; Figure S3A,B, Supporting Information; for full results, see Text S5, Supporting Information). This pattern aligns with findings in existing literature.^[^ ^10^ ^]^ In addition, successful implementation of an inhibition strategy correlated with stronger frontoparietal activation across both modalities (Auditory: *r* = −0.41, *p*(FDR) = 0.014; Visual: *r*‐values ≤ −0.29, *p*(FDR)‐values = 0.036) [Figure S3C,D and Table S5, Supporting Information] and more pronounced sensory region deactivation (Auditory: *r* = 0.60, *p*(FDR) < 0.001; Visual: *r‐*values = 0.31, *p*(FDR)‐values = 0.036) [Figure S3 C,D and Table S5, Supporting Information], further supporting the supramodal nature of inhibition mechanisms (SI‐Text S7).

To verify whether prefrontal regions drive the observed sensory deactivation during inhibition, we used DCM (Figure S4A,B and ‐Text S6, Supporting Information; see “Experimental Section‐Data recording and analysis‐MRI data” – “DCM” for method details), which revealed that this activation‐suppression pattern was driven by significant top‐down inhibitory modulation from the DLPFC to the corresponding sensory cortex (Auditory: ‐0.68 [‐1.02, ‐0.35]; Visual: ‐2.24 [‐1.31, ‐0.86]) (Figure S4C, Supporting Information). More importantly, our analyses revealed a functional dissociation between the sensory cortex and the default model network (DMN) during the inhibition process. This dissociation was not only evident in their distinct activation (Auditory: *p*(FDR) < 0.001, Visual: *p*(FDR) = 0.017; Figure S4D,E and Table S6, Supporting Information) and temporal deactivation (Auditory: *p* < 0.001, Visual: < 0.001; Text S5 and Figure S4D,E, Supporting Information) profiles but was most critically demonstrated by their differential connectivity patterns with the DLPFC. Our DCM results showed that the top‐down modulatory influence exerted by the DLPFC on the sensory cortex was significantly different from its influence on the DMN during the inhibition task (Auditory: *t* ^[^ ^31^ ^]^ = −2.01, *p* = 0.053, *d* = −0.36; Visual: *t* ^[^ ^24^ ^]^ = −3.55, *p* = 0.002, *d* = −0.68; Figure S4C and Table S7, Supporting Information). This finding provides strong evidence that the sensory deactivation observed in our study was not a simple extension of general DMN suppression,^[^ ^26^ ^]^ but rather a functionally specific and actively regulated process.

#### 2.1.3 Oscillatory Signatures of The Inhibitory State (Electroencephalogram, EEG)

Using high‐temporal‐resolution EEG (see “Experimental Section‐Data recording and analysis‐EEG data” for method details), we investigated traveling waves along the occipital‐frontal axis and temporal‐frontal axis to characterize large‐scale neural dynamics during inhibition. We found that inhibition enhanced both forward (occipital‐frontal axis [Figure S5B, Supporting Information]: *t* ^[^ ^39^ ^]^ = 4.33, *p*(FDR) < 0.001; temporal‐frontal axis [Figure S5C, Supporting Information]: *t* ^[^ ^39^ ^]^ = 3.06, *p*(FDR) = 0.008) and backward theta‐band waves (occipital‐frontal axis: *t* ^[^ ^39^ ^]^ = 4.10, *p*(FDR) < 0.001; temporal‐frontal axis: *t* ^[^ ^39^ ^]^ = 4.17, *p*(FDR) < 0.001) along these pathways. Simultaneously, inhibition suppressed beta‐band activity in both directions across both axes (forward waves‐occipital‐frontal axis: *t* ^[^ ^39^ ^]^ = ‐2.88, *p*(FDR) = 0.012; temporal‐frontal axis: *t* ^[^ ^39^ ^]^ = −3.15, *p*(FDR) = 0.008; backward waves‐occipital‐frontal axis: *t* ^[^ ^39^ ^]^ = −3.87, *p*(FDR) < 0.001; temporal‐frontal axis: *t* ^[^ ^39^ ^]^ = −3.01, *p*(FDR) = 0.008). In addition, we observed a direction‐specific enhancement of alpha power specifically for backward waves (occipital‐frontal axis: *t* ^[^ ^39^ ^]^ = 7.04, *p*(FDR) < 0.001; temporal‐frontal axis: *t* ^[^ ^39^ ^]^ = 2.88, *p*(FDR) = 0.008). This spectral profile‐reduced high‐frequency synchronization and enhanced alpha power align with the deactivation‐dominant pattern observed in fMRI (see Section 2.1.5), suggesting that inhibition stabilizes a low‐resource state by suppressing active cortical communication^[^ ^27^, ^28^ ^]^ and engaging rhythmic suppression mechanisms;^[^ ^29^ ^]^ Text S8, Supporting Information). In addition, using EEG source localization, we found that the differences in neural activity between conditions mainly originated from our predefined DLPFC and PPC (Table S8 and Figure S5D, Supporting Information).

#### 2.1.4 Temporal Boundary in Inhibition (EEG)

We aimed to characterize the temporal evolution of the neural mechanisms underlying sustained inhibition. Therefore, we sought to divide the 6‐s inhibition period into distinct early and late phases for analysis. This methodological choice is supported by previous evidence demonstrating distinct neural responses during different temporal phases of short‐duration stimuli. Specifically, early and late response segments of short‐duration events have been shown to differ in both their spatial specificity and their temporal dynamics, reflecting distinct cognitive and neurophysiological processes.^[^ ^30^ ^]^ Moreover, recent advances in event‐related fMRI analyses, including spatiotemporal segmentation methods, underscore the importance and validity of examining short cognitive events at a finer temporal resolution.^[^ ^31^, ^32^ ^]^ To establish a data‐driven and principled basis for this division, we first used the high‐temporal‐resolution EEG data. A change‐point analysis applied to the temporal decoding accuracy curves revealed a significant and robust shift in neural dynamics occurring consistently at approximately 3s post‐stimulus onset (Figure S5 E—G, Supporting Information). The identification of this temporal boundary around 3s was highly robust. It was consistently observed across different stimulus durations (i.e., in 6 and 10‐s stimuli, although absent in 3‐s stimuli where the process likely did not fully unfold); it was further validated across different analytical approaches (Figure S5 H—J, Supporting Information), including analyses onwhole‐scalp and fMRI‐guided electrode selections (see Text S9, Supporting Information for detailed results). Informed by this empirically identified neural transition point, we segmented our subsequent fMRI analyses into early (0–3s) and late phases (3–6 s). This EEG‐guided approach allowed us to meaningfully investigate the evolution of brain activity patterns over time, based on an observable shift in neural processing, instead of relying on an arbitrary division.

#### 2.1.5 Deactivation‐Dominant Neural Dynamics in Inhibition (fMRI)

Further analyses around the 3‐s boundary in fMRI revealed a strikingly conserved temporal neural pattern in both modalities: initial strong frontoparietal activation, followed by gradual diminution (Figure 2D; Table S3 and S4, Supporting Information), coinciding with progressively increasing sensory region deactivation (Figure 2D). This temporal deactivation dynamic was identified across auditory and visual modalities (Text S5, Supporting Information). Notably, more successful inhibition implementation was associated with more pronounced temporal deactivation dynamic (post‐3s *β*_(IH‐NP)_‐pre‐3s *β*_(IH‐NP)_) in sensory regions across both modalities (Auditory: *r* = 0.45, *p*(FDR) < 0.001; Visual: *r* = 0.40, *p*(FDR) < 0.001) [Figure S6A‐B, Table S9, and Text S7, Supporting Information]. Thus, inhibition effectiveness is significantly associated with the temporal coordination and amplification of neural deactivation. Together, these findings establish inhibition as a cross‐modal control.

### 2.2 Cross‐Modality Consistency of Inhibition

We observed similar patterns of neural temporal deactivation during the inhibition process in both the visual and auditory modalities. Therefore, we further investigated the cross‐modal characteristics of the inhibition mechanism. Cross‐modal consistency describes the implementation of the same cognitive process through shared supramodal (i.e., modality‐independent) neural mechanisms across different sensory channels.^[^ ^33^ ^]^ We examined the cross‐modal neural manifestations of inhibition from multiple analytical perspectives.

#### 2.2.1 Cross‐Modal Generalizability of Neural Patterns (fMRI)

**Figure 3:**
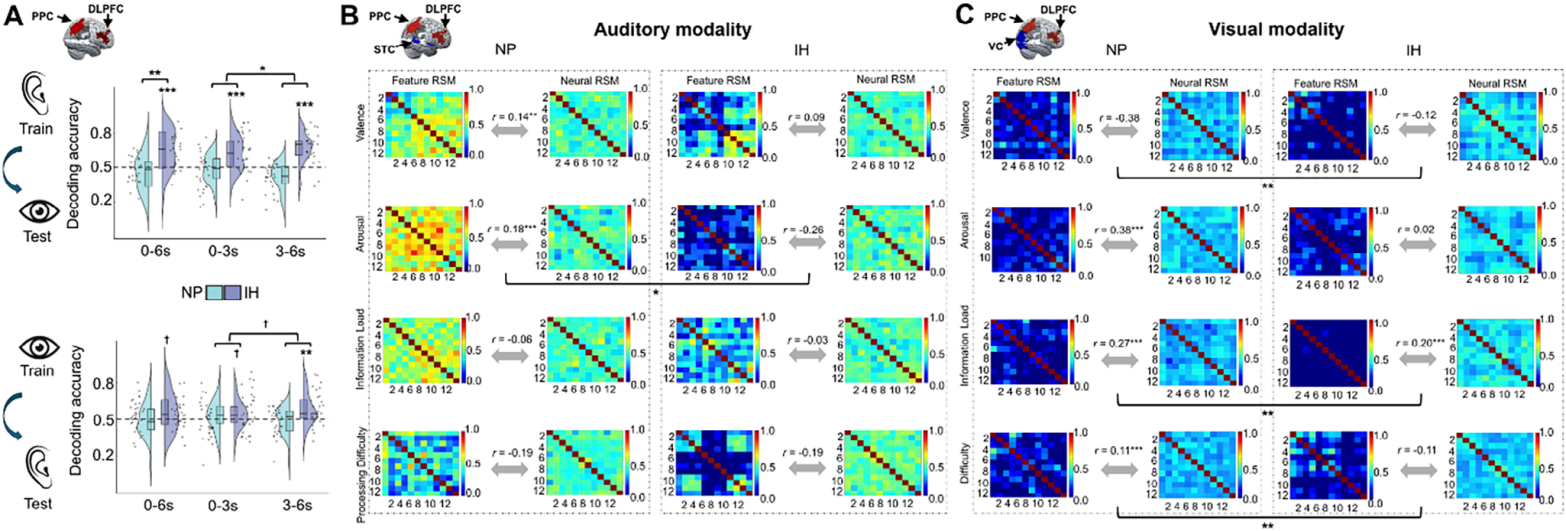
Cross‐modality consistency of inhibition. A) Decoding performance for cross‐modal decoding, trained on auditory modality and tested on visual modality (top panel) and vice versa (bottom panel). The analysis was conducted at the ROI level using the frontoparietal ROI, which was defined by combining regions (DLPFC and PPC) consistently involved in the auditory and visual experiments. This approach was selected to facilitate cross‐modal analysis. Raincloud plots show the distribution of decoding accuracy. Asterisks (*) indicate significant decodability in each ROI. B,C) Correlation analysis between the feature and neural RSMs under NP and inhibition conditions in the auditory (B) and visual (C) modalities. The analysis was conducted at the ROI level. For the auditory experiment, the ROIs included frontoparietal and STC; for the visual experiment, the ROIs included frontoparietal and VC. This modality‐specific approach was adopted to capture how feature representations are processed in the domain‐general control network (frontoparietal) and respective sensory cortices specialized for each modality (STC for auditory and VC for visual), allowing us to examine the shared mechanisms and modality‐specific processing within a consistent analytical framework. The individual cells on the RSM plots represent trial‐wise features or beta values. Connecting lines with * represent significant differences between conditions. Data were obtained from Experiment 1, involving independent participant samples (Auditory: n = 32; Visual: n = 27). Each data point represents an individual participant (biological replicate). The decoding accuracy for each condition was statistically tested against chance level (0.5) using a one‐sample t‐test. Statistical comparisons between inhibition and NP conditions were performed using paired t‐tests. The significance of correlations between neural RSMs was determined using Spearman correlation coefficients. Correlations between neural‐feature RSMs under inhibition versus NP conditions were further compared using Steiger's z‐tests. *p(FDR) < 0.05, **p(FDR) < 0.01, ***p(FDR) < 0.001 (two‐sided).

To test whether inhibition recruits supramodal neural processes, we conducted cross‐modal decoding analyses^[^ ^34^ ^]^ using a frontoparietal region of interest (ROI) encompassing regions consistently activated in auditory and visual tasks (see “Methods‐Data Recording and Analysis‐MRI Data‐Decoding Analysis”). A linear support vector machine (SVM) classifier trained to distinguish inhibition from NP in one modality successfully decoded inhibition states in the other modality (Auditory→Visual: 64.7% accuracy, *p*(FDR) < 0.001; Visual→Auditory: 55.8%, *p* = 0.043 [not survived after FDR but marginally significant]) [**Figure** **3**A; Table S10 and S11, Supporting Information]. Critically, this cross‐modal decoding was specific to the inhibition condition: generalization failed during NP (*p*(FDR)‐values > 0.05) [Figure 3A], ruling out task structure confounds and confirming the unique supramodal signature of inhibition (Text S10, Supporting Information).

Temporal analysis revealed a dynamic improvement in cross‐modal accuracy. Early‐phase (0‐3s post‐stimulus) generalization was significant for inhibition (Auditory→Visual: 61.8%, *p*(FDR) < 0.001; Visual→Auditory: 54.2%, *p* = 0.042 [not survived after FDR but marginally significant]) [Figure 3A and Tables S11 and S12, Supporting Information] but absent for NP. This effect was amplified in the late phase (3‐6s post‐stimulus; Auditory→Visual: 66.8%, *p*(FDR) < 0.001; Visual→Auditory: 58.3%, *p*(FDR) = 0.005) [Figure 3A; Tables S11 and S12, Supporting Information]. We statistically compared the differences between the early and late phases in determining the decoding accuracy. We found that the inhibition‐NP generalization gap widened significantly over time (Auditory→Visual: *t* ^[^ ^26^ ^]^ = 2.61, *p*(FDR) = 0.030; Visual→Auditory: *t* ^[^ ^31^ ^]^ = 1.97, *p*(FDR) = 0.057 [marginally significant]), suggesting progressive stabilization of a cross‐modal inhibition state (Figure 3A; Tables S10 and S11, and Text S10, Supporting Information).

#### 2.2.2 Feature‐Specific Attenuation Across Modalities (fMRI)

We investigated whether inhibition affects the brain processing of various information features and whether these effects show cross‐modal consistency. We conducted representational similarity analysis (RSA)^[^ ^35^ ^]^ on fMRI data linking neural patterns to four subjective dimensions: valence, arousal, processing difficulty, and information load (derived from behavioral ratings collected from a separate, demographically matched sample; see “Experimental materials” for detailed methods and Text S2, Supporting Information for detailed results). By constructing representational similarity matrices (RSMs) for subjective features and neural activity, we analyzed the relationship between feature‐based and neural RSMs to identify which features maintain neural representation under inhibition and which are successfully suppressed.

To quantify inhibition‐induced decoupling, we compared neural‐feature RSM correlations between inhibition and NP conditions. We found that inhibition decoupled the alignment between neural RSMs and emotional or perceptual feature RSMs across both modalities, although with feature‐specific and modality‐specific patterns. In the auditory domain, inhibition primarily affected arousal representation (*Δr* = −0.44, Steigers’*z* = −2.81, *p*(FDR) = 0.005), with insignificant effects on other features (Figure 3B). In the visual domain, inhibition significantly reduced neural representation of multiple features (valence, information load, and processing difficulty, all *p*(FDR)‐values ≤ 0.003), demonstrating broad attenuation of feature encoding (Figure 3C). Despite these modality‐specific differences, both conditions showed a similar pattern of feature decoupling, with arousal showing the most consistent effects across modalities (Text S11, Supporting Information).

These results indicate that inhibition uses a feature attenuation approach in both modalities, although the most affected specific features may depend on the sensory channel. This pattern likely reflects modality‐specific neural representation nuances. Future research could systematically explore these differences.

### 2.3 Multivariate Pattern Analysis Reveals the Neural Dynamics of Inhibition

**Figure 4:**
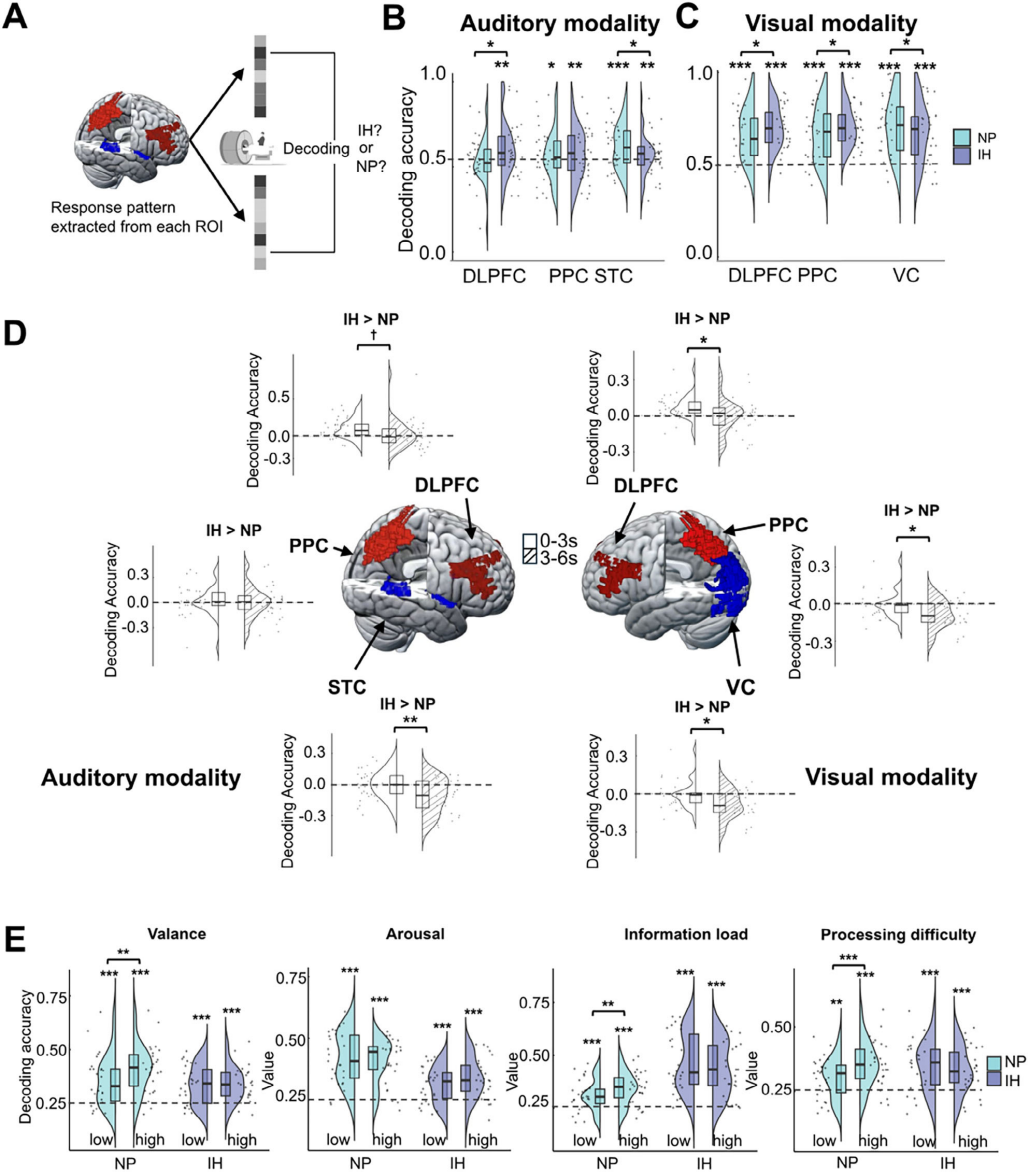
Inhibition‐related neural coding. A) Schematic of fMRI decoding analysis in ROIs, showing cross‐modal classification between NP and inhibition conditions. B,C) ROI decoding performance comparing NP and inhibition conditions in auditory (B) and visual (C) modalities. D) ROI decoding accuracy differences (inhibition > NP) during early (0–3 s) and late (3–6 s) periods for auditory (left panel) and visual (right panel) modalities. Brain maps show ROI locations in the Montreal Neurological Institute (MNI) space: DLPFC (dark red), PPC (light red), and STC/VC (blue). Raincloud plots depict decoding accuracy differences (inhibition > NP), segmented into early and late periods. E) Decoding performance in the combined ROI (DLPFC/PPC/VC) to distinguish the following four conditions: high and low intensities of information features under the inhibition state, and high and low intensities of information features under the NP state. Raincloud plots depict decoding accuracy. Data were obtained from Experiment 1, involving independent participant samples (Auditory: n = 32; Visual: n = 27). Each data point represents an individual participant (biological replicate). The decoding accuracy for each condition was statistically tested against chance level (0.5 or 0.25) using a one‐sample *t*‐test. Statistical comparisons (inhibition vs NP conditions, pre‐3s vs post‐3s periods) were performed using paired t‐tests. *p(FDR) < 0.05, **p(FDR) < 0.01, ***p(FDR) < 0.001 (two‐sided).

Although univariate GLM analyses identify focal activations, they overlook distributed neural representations, which may provide more information to improve the understanding of the cognitive functions.^[^ ^36^ ^]^ Therefore, we used MVPA to characterize how inhibition dynamically reconfigures population‐level neural representations. An SVM classifier with leave‐one‐run‐out cross‐validation was trained to decode inhibition versus NP states within predefined ROIs (see “Methods‐Data Recording and Analysis‐MRI Data‐Decoding Analysis”). The above‐chance ability of the classifier to decode inhibition and NP conditions indicates that voxel patterns within specific ROIs contain sufficient information to distinguish between cognitive states, demonstrating condition‐specific neural coding (**Figure** **4**A).^[^ ^25^ ^]^

During the whole‐time phase (0–6 s post‐stimulus), the classifier achieved significantly above‐chance accuracy (50%) in all ROIs (except for DLPFC during NP state in the auditory task) of all modalities during the inhibition (accuracies > 57.4%, *p*(FDR)‐values ≤ 0.008) [Figure 4B,C] and NP states (accuracies > 57.2%, *p*(FDR)‐values ≤ 0.019) [Figure 4B,C, Text S12, and Table S12, Supporting Information]. To ensure that the preprocessing process (smooth or unsmooth) has no impact on MVPA, we compared the MVPA results under different preprocessing methods, which are presented in Text S12 and Table S12 (Supporting Information).

#### 2.3.1 Region‐Specific Coding Profiles (fMRI)

We compared the differences in decoding accuracy between NP and IH under the same ROI. The results revealed two key dissociations: 1) frontoparietal enhancement: inhibition boosted decoding accuracy in executive regions (Auditory: DLPFC *t* ^[^ ^31^ ^]^ = 2.30, *p*(FDR) = 0.042; Visual: DLPFC *t* ^[^ ^26^ ^]^ = 2.31, *p*(FDR) = 0.029; PPC *t* ^[^ ^26^ ^]^ = 2.34, *p*(FDR) = 0.029) [Figure 4B‐C, Table S13a), aligning with their increased activation (as shown in Section 2.1.2); 2) sensory suppression: conversely, inhibition reduced decoding accuracy in modality‐specific cortices (Auditory: STC *t* ^[^ ^31^ ^]^ = ‐2.66, *p*(FDR) = 0.012; Visual: VC *t* ^[^ ^26^ ^]^ = −2.53, *p*(FDR) = 0.029), mirroring their deactivation (Figure 4B‐C; Table S13a and Text S12).

#### 2.3.2 Temporal Evolution of Neural Coding (fMRI)

Based on the above region‐specific coding profiles, comparison of the pre‐3s and post‐3s periods revealed two important temporal trajectories: 1) Progressive attenuation of frontoparietal activation: early inhibition‐related enhancements in executive regions diminished significantly at the late phase (Auditory: DLPFC *t* ^[^ ^31^ ^]^ = −2.12, *p*(FDR) = 0.065 (marginally significant); Visual: DLPFC *t* ^[^ ^26^ ^]^ = −2.37, *p*(FDR) = 0.043; PPC *t* ^[^ ^26^ ^]^ = −2.13, *p*(FDR) = 0.043) [Figure 4D; Table S13b, Supporting Information]; 2) Progressive intensification of sensory deactivation: early inhibition‐related reduction in sensory region declined further over time (Auditory: STC *t* ^[^ ^31^ ^]^ = −3.32, *p*(FDR) = 0.006; Visual: VC *t* ^[^ ^26^ ^]^ = −2.19, *p*(FDR) = 0.043) [Figure 4D; Table S13b, Supporting Information]. These results corresponded to the progressive deactivation observed in univariate analyses (Text S12, Supporting Information).

#### 2.3.3 Global Feature Representation Attenuation (fMRI)

Within a combined ROI (DLPFC/PPC/VC) in the visual modality, inhibition broadly disrupted neural discrimination between high and low levels of stimulus features that were robustly encoded during NP (valence: *F* ^[^ ^1^, ^26^ ^]^ = 6.06, *p* = 0.021; information load: *F* ^[^ ^1^, ^26^ ^]^ = 7.74, *p* = 0.010; processing difficulty: *F* ^[^ ^1^, ^26^ ^]^ = 27.26, *p* < 0.001; the *F*‐tests were derived from simple effects analyses of the task condition*feature intensity interaction) [Figure 4E; Table S14, Supporting Information]. This pan‐feature suppression aligns with RSA findings (Results‐Section 2), confirming that inhibition broadly attenuates the neural representation of information feature (Text S13, Supporting Information).

These findings indicate that inhibition not only progressively diminishes the neural coding capacity but also broadly blurs the encoding of information features, thereby providing supportive evidence for a deactivation‐dominant mechanism in inhibition.

### 2.4 Causal Evidence for the Role of Dorsolateral Prefrontal Cortex in Driving Temporal Deactivation

Previous studies have established that the DLPFC plays a core role in inhibitory control; for example, applying excitatory neuromodulation to this region enhances inhibitory performance,^[^ ^37^ ^]^ whereas inhibitory modulation impairs the performance.^[^ ^38^ ^]^ We hypothesized that this causal role of the DLPFC is achieved not merely through a general suppression but via the modulation of the specific neural dynamics of temporal deactivation in the sensory cortex.

**Figure 5:**
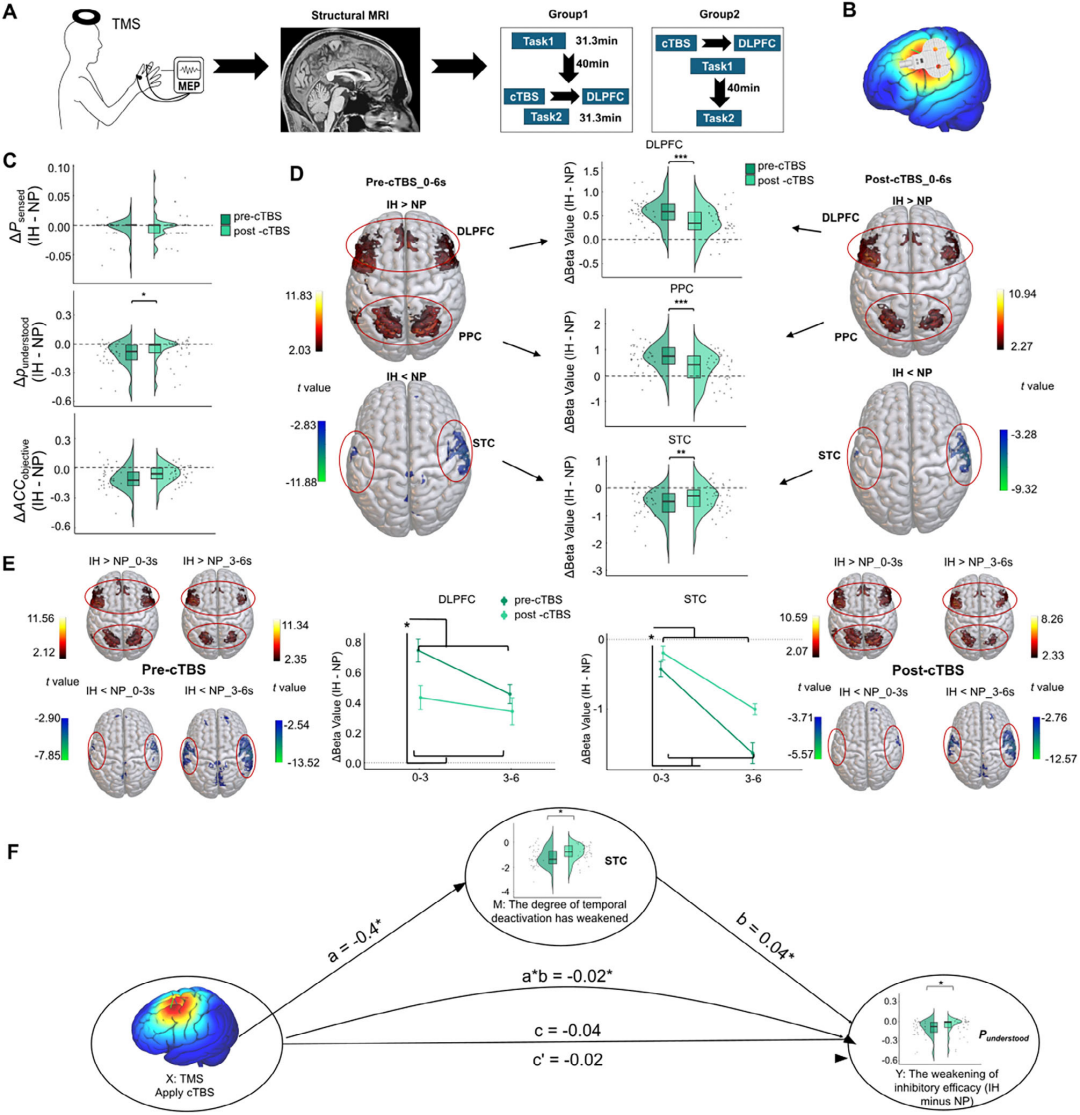
Causal evidence for the role of temporal deactivation in inhibition performance (cTBS‐fMRI). A) Experimental design showing cTBS applied to the left DLPFC followed by fMRI scanning. Participants were randomly assigned to two counterbalanced groups differing in the timing of cTBS administration. B) Transcranial Magnetic Stimulation (TMS) electric fields during cTBS application to the DLPFC, visualized using SimNIBS software (www.simnibs.org). The color scale represents electric field strength, ranging from 0 (blue) to the individual maximum (red). C) Behavioral effects of cTBS. Raincloud plots show the change (Δ) in behavioral performance (perceiving (*P*_sensed_) and understanding the stimuli (*P*_understood_), along with accuracy in identifying the conveyed information (*ACC*_objective_)) for the inhibition condition (IH > NP) after cTBS (post) relative to before (pre). D) Brain activation and deactivation differences comparing pre‐cTBS and post‐cTBS conditions during the 0–6 s period. Raincloud plots show the distribution of beta values for IH > NP contrast in the DLPFC, PPC, and STC regions. E) Temporal dynamics showing the progressive change from the 0–3 s to 3–6 s periods for the DLPFC (left) and STC (right) regions, comparing pre‐cTBS (dark green) and post‐cTBS (light green) conditions. All the voxels presented are statistically significant. Line charts (centre) display mean ± SEM. F. Mediation analysis demonstrating the causal pathway from cTBS intervention to behavioral inhibitory performance via temporal deactivation. The diagram shows path coefficients, with the indirect effect (a × b = −0.02, 95% CI [‐0.04, ‐0.0009]) indicating that cTBS impairs behavioral inhibition by disrupting the temporal deactivation dynamic in the sensory cortex. Data were obtained from Experiment 4 (n = 36). Each data point represents an individual participant (biological replicate). Statistical comparisons between pre‐cTBS and post‐cTBS conditions were performed using paired t‐tests. *p(FDR) < 0.05, **p(FDR) < 0.01, ***p(FDR) < 0.001 (two‐sided). All quantitative analyses were conducted using the full anatomical ROI masks, with colored voxels within each mask denoting the regions of maximal statistical significance.

To directly test this hypothesis, we used, in Experiment 4, inhibitory cTBS to temporarily interfere with the function of the left DLPFC and observe its effects on the auditory inhibition task (**Figure** **5**A,B, see “Experimental Section – Experimental Procedure – Experiment 4” for method details). Compared to the pre‐intervention state, cTBS significantly decreased the inhibition efficacy (Figure 5C) and weakened the overall neural signatures of inhibition (reducing activation in the DLPFC/PPC and deactivation in the STC; Figure 5D and Tables S15a and S16, Supporting Information). More critically, cTBS specifically disrupted our key hypothesized mechanism: it significantly attenuated the characteristic temporal deactivation dynamic—the progressive increase in deactivation from the early to the late phase—in the STC (*t* ^[^ ^35^ ^]^ = −2.69, *p*(FDR) = 0.033, *d* = −0.45) [Figure 5E, Tables S15b and S16 Text S14, Supporting Information].

To elucidate the causal chain between DLPFC function, the temporal deactivation dynamic, and behavioral inhibitory performance, we conducted a mediation analysis. The results provided decisive evidence: the negative effect of cTBS on behavioral inhibitory performance was fully mediated by the weakening of the temporal deactivation dynamic in the sensory cortex (indirect effect a × b = ‐0.02, 95% CI [‐0.04, ‐0.0009]) [Figure 5F; Text S15, Supporting Information], whereas the direct effect of cTBS on behavior was insignificant. This finding directly demonstrates that temporal deactivation is not merely an epiphenomenon of the inhibition process but an important causal mechanism actively driven by the DLPFC, and it is crucial for achieving successful behavioral inhibition.

### 2.5 From Inhibition to Managing Reduced Processing Demand: The Deactivation Response of the Brain

Although our prior findings established temporal deactivation patterns during inhibition—characterized by progressive frontoparietal decay and sensory suppression—a critical question remains: Is this mechanism specific to active inhibition, or does it reflect a general response to reduced processing demand?

#### 2.5.1 Experimental Dissection of Processing Demand

To address the above‐mentioned question, we operationalized information processing demand across three experimental conditions: 1) Active inhibition: involves a subjective reduction in information processing (high demand initially→low demand after regulation), implemented through deliberately inhibiting information intake; 2) Active reception (concentration): maintains high information processing demand throughout (high demand→sustained high demand), implemented through active focus on stimuli; 3) Passive deprivation: presents objectively low information validity that naturally requires minimal processing (low demand→low demand), implemented through auditory scrambling (replacing original stimuli with scrambled voice) or input blockade (eye closure). Behavioral results and neural profiles during the entire time phase (0–6 s post‐stimulus) across these three conditions are presented in Figure S7A,B (Supporting Information) and detailed in Texts S16 and S17 (Supporting Information).

#### 2.5.2 Analytical Approaches

We used two complementary methods to compare the neural representations across different scenarios. The first method examined the temporal dynamics of neural activity patterns. We analyzed the changes in brain activation and deactivation patterns across the early (0–3 s) and late (3–6 s) phases. This analysis allowed tracking of the temporal progression of neural responses and identification of scenario‐specific patterns of activation change over time. By comparing these temporal signatures across conditions, we could determine whether different scenarios exhibited similar or distinct patterns of neural dynamics. The second method was RSA, which is particularly effective for comparing patterns of neural representation across different experimental conditions^[^ ^35^ ^]^ (see “Experimental Section‐Data recording and analysis‐MRI data‐RSA”). We constructed RSMs for each scenario based on the neural activation patterns across all voxels in our ROIs. By quantifying the alignment between RSMs from different scenarios, we could assess which conditions generated similar neural representations to those observed during inhibition.

#### 2.5.3 Double Dissociation–Processing Demand Dictates Neural Dynamics (fMRI)

**Figure 6:**
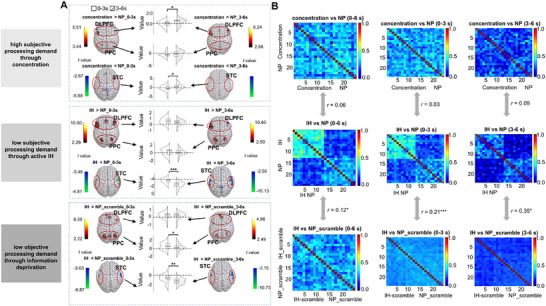
The relationship between inhibition and brain deactivation patterns. The left column indicates the processing demands associated with each experimental condition: high subjective processing demand through concentration (top), low subjective processing demand through active inhibition (middle), and low objective processing demand through information deprivation (bottom). A) Brain activation and deactivation differences for the following comparisons during early (0–3 s) and late (3–6 s) periods: concentration versus NP (top panel), inhibition versus NP (middle panel), inhibition versus NP‐scramble (bottom panel; NP‐scramble: passive listening to scrambled auditory stimuli). Brain maps display activation and deactivation, and raincloud plots depict beta values, divided into early and late periods. All the voxels presented are statistically significant. B) Correlations between neural RSMs for the following contrasts across different time windows (0–6 s, 0–3 s, 3–6 s) in auditory experiment: concentration versus NP and inhibition versus NP, as well as inhibition versus NP and inhibition versus NP‐scramble. Data were from Experiment 3, involving independent participant samples (Auditory: n = 15; Visual: n = 27). Each data point represents an individual participant (biological replicate). Statistical comparisons between pre‐3s versus post‐3s periods were performed using paired t‐tests. The significance of the correlations between neural RSMs was determined using pearson correlation coefficients. *p(FDR) < 0.05, **p(FDR) < 0.01, ***p(FDR) < 0.001 (two‐sided). All quantitative analyses were conducted using the full anatomical ROI masks, with colored voxels within each mask denoting the regions of maximal statistical significance.

Our analyses revealed a significant double dissociation that demonstrates the brain's response to processing demand:

1) When processing demand increases (comparing active reception to active inhibition): During the concentration condition, neural dynamics reversed the pattern observed during active inhibition. The frontoparietal regions showed progressive activation (low‐to‐high pattern), whereas the sensory regions displayed diminishing deactivation (high‐to‐low pattern) [**Figure** **6**A; Tables S17 and S18 and Text S16, Supporting Information]. RSA confirmed this functional distinction, revealing minimal similarity between concentration and inhibition neural patterns across different time windows (0–6 s, 0–3 s, 3–6 s; *r*‐values ≤ 0.09, *p*(FDR)‐values > 0.350) [Figure 6B; Text S18, Supporting Information]. Therefore, the results indicate the distinction in neural patterns corresponding to high versus low subjective processing demands.

2) When processing demand decreases (comparing passive deprivation to active inhibition): Conditions of passive deprivation reproduced inhibition‐related neural dynamic patterns. Scrambled voice conditions generated temporal deactivation patterns similar to active inhibition (Figure 6A; Tables S19 and S20, Supporting Information). Text S17 (Supporting Information) provides the full results. RSA confirmed this functional distinction. Both scrambled voice (*r*‐values ≥ 0.12, *p*(FDR) ‐values ≤ 0.044) [Figure 6B] and eye closure (*r*‐values > 0.87, *p*(FDR)‐values < 0.001) [Figure S7C, Supporting Information] conditions showed high neural pattern similarity with their related inhibition conditions (Text S18, Supporting Information). Therefore, the results indicate a shared neural representation of reduced processing demand.

Together, these findings confirmed functional specificity (temporal deactivation patterns when processing demand is low but not when it is high) and universality (similar deactivation patterns emerge regardless of whether the reduction in processing demand originates from subjective strategies [active inhibition] or objective deficiencies [passive deprivation]).

## 3 Discussion

### 3.1 Direct and Competition‐Induced Inhibition Both Involved a Deactivation Temporal Dynamic

Although traditional inhibitory mechanisms—such as direct inhibition in memory or motor control^[^ ^1^ ^]^ or competition‐induced inhibition in attention^[^ ^6^ ^]^—have historically emphasized specificity and been studied as isolated processes, our findings highlight that both converge on a common neural implementation: a shared neural deactivation dynamic.

Conjunction analyses capturing neural commonalities revealed that both inhibition types consistently engage the frontoparietal control network (DLPFC and PPC) while suppressing sensory regions (auditory/visual cortices); besides, more critically, they exhibit an identical temporal deactivation dynamic. This dynamic is characterized by brain activity patterns and neural coding capabilities. Initially, strong frontoparietal activation and high decoding performance within executive regions were observed, reflecting an early engagement of cognitive control mechanisms; this finding is consistent with previous studies on executive‐control.^[^ ^13^, ^39^ ^]^ Our DCM analysis confirmed this top‐down directionality, revealing a significant inhibitory effect from the DLPFC to sensory cortices during inhibition. However, these initial activation effects progressively diminished over time. Concurrently, sensory regions showed progressive deactivation and reduced inhibition decoding performance, becoming more pronounced particularly after the 3‐s boundary. In addition, more effective inhibition was associated with stronger deactivation dynamics within sensory regions, suggesting that deactivation—particularly its progressive amplification over time—is related to the success of inhibition rather than reflecting random or spontaneous neural fluctuations. These convergences position inhibition as a foundational cognitive control system for information rejection, transcending tactical differences in implementation.

### 3.2 The Primacy of Deactivation: a Dynamic Spatiotemporal Framework

The classical view of inhibition as a static “neural brake” fails to capture its temporal essence.^[^ ^1^, ^10^ ^]^ In this study, we observed “activation decay coupled with deactivation amplification” trajectory in brain activity patterns and coding capabilities, which gives us a novel spatiotemporal signature: inhibition is not merely associated with static neural intensity, but is fundamentally characterized by its temporal unfolding—a feedforward neural signal attenuation. The suppression of beta oscillations, critical for active cortical communication^[^ ^23^ ^]^ as revealed by traveling wave analysis, aligns with this neural attenuation. Besides, the low‐energy theta frequency band maintains the connection between the primary sensory cortex and prefrontal cortex. This spectral profile suggests that inhibition stabilizes a low‐resource state by tonically suppressing high‐frequency processing while rhythmically gating irrelevant inputs through alpha oscillations.^[^ ^29^ ^]^

These dynamics generalize beyond specific modalities or contexts. Identical deactivation patterns emerged in both auditory and visual inhibition tasks, with cross‐modal similarity scores exceeding chance levels. Notably, input deprivation experiments dissociated deactivation from intentional control: artificially degrading sensory input clarity (scramble voices or eye closure) replicated the inhibition‐like deactivation trajectory. This convergence implies that deactivation serves as a universal adaptive solution—whether invoked by top‐down control (“active rejection”) or imposed by bottom‐up constraints (“passive deprivation”). The causal nature of this active rejection was directly tested in our cTBS experiment; temporarily disrupting the DLPFC, the source of top‐down control, impaired the ability of the brain to implement the deactivation dynamic and adversely affected behavioral inhibition. Such findings further demonstrate the neural efficiency hypothesis,^[^ ^40^ ^]^ which emphasizes minimizing activity in task‐irrelevant regions. In our study, deactivation emerged not merely as a resource‐conserving mechanism but as an active, domain‐agnostic strategy for optimizing information processing, even in regions traditionally considered task‐relevant.

Collectively, these findings suggest a shift in understanding deactivation. Although prior models relegated deactivation to a supportive role—facilitating resource reallocation or suppressing distractors^[^ ^41^, ^42^, ^43^, ^44^, ^45^ ^]^—our evidence underscores its primacy. Three lines of evidence solidify this claim: 1) Temporal specificity: deactivation amplification directly predicts behavioral success, excluding explanations based on random neural fluctuations; 2) Functional necessity: The coupled decay of frontoparietal activation and strenghtening of sensory deactivation forms a closed‐loop control system. Our DCM and cTBS results causally validated this as a top‐down inhibitory pathway crucial for successful inhibition; and 3) Systemic universality: robust replication across strategies, modalities, and experimental manipulations confirms the foundational role of deactivation. These insights suggest reconceptualizing inhibition as a deactivation‐dominant process, where progressive neural signal attenuation—not sustained activation—is the core mechanism. This framework bridges diverse inhibitory phenomena, providing a unified explanation of how the brain adaptively gates information flow through dynamic spatiotemporal dynamics.

### 3.3 Cross‐Modal Consistency in Inhibition

Inhibition is not associated with a single sensory modality; instead, it appears to recruit largely overlapping neural processes, regardless of whether auditory or visual input is inhibited.

Specifically, inhibition showed a significant degree of consistency across sensory modalities. Auditory and visual inhibition tasks activated highly similar frontoparietal control networks while producing deactivation in corresponding sensory regions, with the same temporal progression of deactivation. Although this findings align with previous findings on supramodal attention models emphasizing shared control substrates across senses,^[^ ^46^, ^47^ ^]^ inhibition uniquely extends these frameworks by revealing that cross‐modal generalization applies not just to information reception (through attentional selection^[^ ^47^ ^]^) but also to information rejection (through inhibition).

Building on the proposed cross‐modal neural generalization (e.g.,^[^ ^48^ ^]^), our cross‐modal decoding analysis provides a nuanced picture. Notably, this generalization was asymmetric: classifiers trained on auditory inhibition could successfully identify inhibition states in the visual modality but the reverse was only marginally effective. Critically, this generalization capability strengthens as inhibition continues, reflecting the transition of a brain to a stable, modality‐agnostic “inhibition state,” a finding that expands prior findings on transient cross‐modal suppression^[^ ^49^ ^]^ by highlighting progressive and sustained deactivation as the neural signature of information rejection.

A notable finding is the asymmetry in its direction: in the suppression task, the classifier trained based on auditory data can robustably predict the states in the visual task, whereas the reverse prediction (from visual to auditory) is less effective, only reaching the edge significance level. In humans, vision is widely regarded as the dominant sensory modality, meaning it often captures attention and better guides perception than other senses, particularly in situations of cross‐modal conflict.^[^ ^50^, ^51^ ^]^ This dominance is reflected neurally by the vast cortical territory dedicated to visual processing compared to auditory processing.^[^ ^52^ ^]^ Suppressing input from a non‐dominant channel like audition may be achieved via a relatively simple, potent, and metabolically efficient top‐down signal from the executive control network. This could generate a robust and generalizable neural pattern of inhibition that can be successfully decoded and applied to the visual modality. Conversely, attempting to suppress the dominant visual stream likely constitutes a more formidable cognitive challenge. Preventing potent visual information from reaching conscious processing may require not just a simple “gate‐closing” signal but a more intricate and resource‐intensive reallocation of neural resources.^[^ ^53^ ^]^ This inhibitory pattern may be more complex, nuanced, and “visual‐centric”, making its core neural features less readily transferable to the distinct processing architecture of the auditory system.

The cross‐modal universality of inhibition is further evidenced by its supramodal attenuation of input quality. RSA indicated a broad decoupling between stimulus features (e.g., arousal) and their corresponding neural representations, irrespective of sensory modality (auditory or visual). This modality‐invariant attenuation of feature encoding extends efficient coding principles^[^ ^54^ ^]^ into the aspect of active cognitive control. Whereas traditional efficient coding emphasizes passive mechanisms—such as sensory systems reducing redundant signals by adapting to statistical regularities (e.g., retinal adaptation^[^ ^55^ ^]^)—inhibition represents an active, top‐down, and goal‐directed approach that reduces information input.

### 3.4 Information Blurring During Inhibition

Evidence suggests that as inhibitory processes persist, information processing progressively weakens, resulting in information degradation or loss.^[^ ^20^, ^21^ ^]^ Our findings provide empirical support for this “information blurring” phenomenon. Behaviorally, inhibition induces cascading deficits across multiple cognitive stages, i.e., inhibition significantly reduces perceptual/attentional clarity, comprehension accuracy, and objective memory retention, indicating that inhibition may reduce the clarity of incoming information.

Neural evidence supports the concept of information blurring through several key findings. MVPA revealed a significant reduction in neural discriminability between high and low levels of emotional valence, informational load, and processing difficulty. In addition, RSA indicated a broad decoupling between stimulus features and their corresponding neural representations, further confirming that the brain actively “blurs” the clarity of input information. Moreover, our study found similar neural deactivation patterns between active inhibition and passive input deprivation conditions (e.g., auditory scrambling or visual deprivation via eye closure). This alignment suggests that information suppression of the brain through internal inhibition closely resembles the neural signatures associated with inputs that have been externally degraded, further supporting the notion that inhibition operates by reducing input clarity.

### 3.5 The Brain Adopts a Deactivation Mode not Just for Inhibition, but for Generalized Low Processing Demand

The temporal deactivation pattern is not limited to inhibition but appears universally in scenarios of low processing demand. Our results directly support this claim through a carefully designed comparison between active inhibition, active concentration (high processing demand), and passive deprivation (low processing demand) conditions.

Specifically, in conditions involving high processing demand (active concentration), participants exhibited an opposite neural dynamic compared to inhibition: concentration progressively amplified frontoparietal activation (low‐to‐high) while simultaneously reducing sensory region deactivation (high‐to‐low). This pattern aligns closely with previous findings from attention research, which emphasizes increased neural engagement during sustained, effortful attention.^[^ ^56^ ^]^ In contrast, our inhibition condition exhibited the reverse pattern (progressively decreasing frontoparietal activation paired with increasing sensory deactivation), which clearly distinguishes it from the active concentration condition. Neural RSA further confirmed this dissociation, demonstrating that the neural representations between inhibition and active concentration states showed low similarity, reinforcing the specificity of the deactivation pattern to low‐demand contexts rather than active cognitive engagement.

To further verify that the temporal deactivation pattern reflects a generalized neural response to low processing demand, we introduced passive deprivation conditions (e.g., scrambled voices, visual input deprivation via eye closure). Notably, these passive deprivation manipulations reproduced inhibition‐like temporal deactivation patterns. Neural RSA analyses revealed significant representational similarities between active inhibition conditions and passive deprivation conditions, suggesting that the neural response of the brain to externally reduced input clarity closely mirrors internally driven inhibition processes.

These results improve the findings of previous studies, which primarily attributed cortical deactivation under passive deprivation conditions to reduced bottom‐up signal quality.^[^ ^57^ ^]^ Our data indicate that top‐down, active cognitive processes (inhibition) produce strikingly similar neural patterns as passive bottom‐up‐driven reductions in sensory input. This convergence holds considerable promise for unifying previously distinct research areas—namely cognitive control mechanisms^[^ ^13^ ^]^ and sensory deprivation effects^[^ ^58^ ^]^—under a coherent, deactivation‐centric theoretical framework.

Taken together, our findings support a broader cognitive principle: the brain consistently adopts a similar neural strategy—characterized by temporal deactivation—to manage information processing in scenarios where processing demand is low or the information value is minimal, irrespective of whether this demand reduction originates from internal (endogenous) goals or external (exogenous) stimulus conditions.

In the processing of information deemed unnecessary or of low value, the brain could theoretically use two strategies:^[^ ^1^ ^]^ investing more cognitive resources to activate specific neural mechanisms that inhibit the reception of information or^[^ ^2^ ^]^ reducing resource investment, thereby diminishing the processing of that information. Our research yielded an unexpected finding: the brain predominantly use a temporal deactivation pattern in such a scenario. This phenomenon is not unique to our study but has been observed in other contexts, such as repetition suppression in animal and human studies^[^ ^59^, ^60^ ^]^ and memory suppression in human research,^[^ ^4^ ^]^ where similar deactivation responses were found to be related to reduced processing demands. These findings suggest that deactivation is a widespread mechanism for minimizing information processing, a phenomenon that has not been previously investigated or systematically summarized.

## 4 Experimental Section

Experiments 1–4 were approved by the Ethical Committee at the School of Psychology, Shenzhen University (Approval No. SZU_PSY_2024_067).

### 4.1 Participants

In this study, participants self‐identified their ethnicity as East Asian Chinese, which was subsequently verified against their government‐issued identity cards for accuracy. A total of 278 participants were enrolled across four experiments. Specifically, Experiment 1 involved fMRI assessments and was subdivided into two parts: Auditory (n = 55) and Visual (n = 77). Experiment 2 involved EEG assessments, further subdivided into three parts: 2.1 (n = 30), 2.2 (n = 40), and 2.3 (n = 40). Experiment 3 utilized fMRI and included participants from Experiment 1 who agreed to participate again. Specifically, the auditory experiment involved 15 participants from Experiment 1‐Auditory, while the visual experiment included 27 participants from Experiment 1‐Visual. Experiment 4 combined TMS and fMRI, and included 36 participants. All participants provided informed written consent, and the study procedures were approved by the Shenzhen University Institutional Review Board. Six participants were lost to attrition (four from Experiment 1, two from Experiment 2). Data from the remaining 272 participants were analyzed: Experiment 1 (Auditory, n = 51; Visual, n = 77), Experiment 2 (2.1: n = 30, 2.2: n = 40, 2.3: n = 38), Experiment 3 (Auditory: n = 15, revisited participants from Experiment 1‐Auditory; Visual: n = 27, revisited participants from Experiment 1‐Visual), and Experiment 4 (n = 36). Participants’ demographic and neuropsychological data are summarized in Table S21 (Supporting Information). All participants provided written informed consent and were monetarily compensated for their participation.

An appropriate sample size a priori was determined based on prior experience. To verify whether power was available to detect a significant behavioral difference between task conditions, *post hoc* power analyses using the R package SIMR (https://cran.r‐project.org/web/packages/simr/index.html) were conducted for all predictors in the best‐performing models across the experiments. The analysis revealed that all models from Experiments 1–4 exhibited robust power, ranging from 85% to 100%.

### 4.2 Experimental Materials

The Auditory experimental materials comprised a total of 400 human voice clips, divided into two sets. The normal set consisted of 300 voice clips, with 100 clips each of 3, 6, and 10‐s durations. The scrambled set was generated by converting each of the 100 normal 6‐s voice clips into scrambled counterparts, resulting in an additional 100 scrambled voice clips.

The original texts were in Chinese, and they were translated into English for readability. The normal voice clips were generated through the following process: First, ChatGPT generated a sentence that allowed for a natural speaking speed of 6 s (e.g., “This is a very complex problem. It is difficult to find a solution. It requires professional knowledge and skills”). Based on this 6‐s sentence, GPT then created a shorter sentence with fewer information (3 s speaking speed allowed;, e.g., “This problem is too difficult to solve”), and a longer sentence with more information (10 s speaking speed allowed;, e.g., “This is a very complex problem, difficult to find a solution, requiring professional knowledge and skills. Many people have tried, but there is still no progress, needing more time and resources”). Second, the voice clips were recorded by AI speakers (half male, half female; generated from https://ttsmaker.cn) reading aloud the short or long sentences, at a normal speaking speed, volume, and pitch. Third, for each of the 6‐s and the corresponding short and long sentences, GPT generated a multiple‐choice quiz with four options (e.g., “What did this voice say? A: There might be problems in the future; B: This was an easy problem.; C: This was a complex problem; D: The problem had been solved”). Only one option (in this case, option C) correctly matched the information conveyed in the corresponding voice clip. These quizzes were designed to assess participants’ understanding of the spoken content.

The scrambled voice clips were created by taking the 100 normal 6‐s voice clips and thoroughly scrambling the audio, rendering them meaningless. Voice scrambling was achieved using a custom script in MATLAB. Each of the 100 normal 6‐s voice (from Experiment 1) clips was segmented into short, 50 ms windows. The order of these windows was then randomly permuted, and the segments were re‐synthesized to create a new audio file. This procedure effectively destroys all semantic and phonetic content, rendering the clips unintelligible, while preserving the long‐term spectral characteristics and amplitude envelope of the original speech.

The stimulus set used in Experiment 3 (the passive deprivation conditions) and Experiment 4 (TMS+fMRI) was drawn directly from the materials created for Experiment 1. Specifically, the “scrambled voice” condition in Experiment 3 utilized the same 100 scrambled voice clips that served as counterparts to the normal 6‐s voice clips from Experiment 1, ensuring consistency of materials across the experiments.

The experimental materials for the Visual comprised 100 images randomly selected from the International Affective Picture System (IAPS^[^ ^61^ ^]^). Both the normal voice clips and the IAPS pictures were randomly assigned to four different experimental conditions: 1) NP‐Participants listened to the voice clips or viewed the pictures in a natural manner, without any additional instructions or interventions; 2) direct inhibition‐ Participants were instructed to inhibit or suppress their natural responses while listening to the voice clips or viewing the pictures; 3) Distraction‐Participants were asked to perform a secondary task (e.g., imagining their home) while listening to the voice clips or viewing the pictures; 4) Distancing‐participants were instructed to mentally distance themselves from the content of the voice clips or pictures, viewing them as if they were not personally involved. Detailed instruction texts for each condition can be found in Dataset S1 (Supporting Information).

Balancing randomization and analytical uniformity, an equal number of stimuli was selected for each of the four experimental conditions, maintaining consistency across subsequent experiments. Specifically, each condition included an equal number (n = 25) of either IAPS images or voice clips. The voice clips were 6 s in duration or their corresponding 3‐ or 10‐second versions. To assess potential confounds, It was evaluated whether emotional valence, arousal, information load, and processing difficulty differed significantly between the assigned conditions. An independent sample, demographically matched to the main study participants, rated the stimuli using nine‐point scales: valence (1 = most negative to 9 = most positive), arousal (1 = least arousing to 9 = most arousing), information load (1 = lowest to 9 = highest), and processing difficulty (1 = least difficult to 9 = most difficult). The results indicated that the stimuli were well‐matched across conditions, minimizing potential confounds related to emotional content, information density, or processing difficulty. The full results are presented in Text S2 (Supporting Information). The full text of all voice clips, the IAPS IDs of the pictures, the associated quizzes, descriptive statistics for average ratings and individual stimulus ratings, and all statistical results are provided in Dataset S1 (Supporting Information).

For the EEG experiment, voice clips were presented via headphones (Sennheiser HD200 PRO) at a comfortable level (80–85 decibels (dB) SPL). For the fMRI experiments, voice clips were presented using Media Control Functions (DigiVox, Montreal, Canada) via electrostatic headphones (NordicNeuroLab, Norway, or Sensimetrics, USA) at a comfortable level (80–85 dB SPL).

### 4.3 Behavioral Task

Prior to participating in the auditory/visual experiment, participants received thorough briefings on the detailed instructions associated with each instruction trigger word: “NP”, “direct inhibition”, “distraction”, or “distancing”. Participants were trained to associate each trigger word with its corresponding cognitive strategy (for detailed instructions, see Text S1, Supporting Information).

The experimental paradigm, illustrated in Figure 2A, consisted of the following sequence for each trial:^[^ ^1^ ^]^ Presentation of the instruction trigger word (“NP”, “direct inhibition”, “distraction”, or “distancing”) for 1.5 s;^[^ ^2^ ^]^ A fixation period lasting either 1.5 or 3 s;^[^ ^3^ ^]^ Voice or picture presentation, during which participants applied the strategy indicated by the trigger word for 6 s;^[^ ^4^ ^]^ A “sensory” question, either “Did you hear this voice?”, “for the Auditory experiment” or “Did you see this picture?” for the Visual experiment, presented for 1.5 s;^[^ ^5^ ^]^ An “understand” question, either “Did you understand the meaning of the voice?” for the Auditory experiment or “Did you understand the meaning of the picture?”, “for the Visual experiment”, also presented for 1.5 s;^[^ ^6^ ^]^ An “objective” question, either “What information did this voice convey?”, “for the Auditory experiment” or “What information did this picture convey?” for the Visual experiment, presented with four possible options, including one correct answer, for 4.5 s.

### 4.4 Experimental Procedure

Three experiments were conducted to investigate the neural mechanisms underlying inhibition. In Experiment 1, fMRI was utilized to assess the neural correlates of Auditory and Visual, employing distinct participant samples for each modality. Experiment 2 employed EEG to capture the temporal neural dynamics associated with Auditory. This experiment comprised one main study (2.1), which utilized 6‐s duration voice stimuli, alongside two complementary studies (2.2 and 2.3), each using 3 or 10‐s voice stimuli, with different participant samples for each. Experiment 3 utilized fMRI to verify the specificity of inhibition by comparing its brain activity patterns with those observed during “concentration” and “complete shielding” conditions. While Experiments 1, 2 each utilized different participant samples, Experiment 3 involved a subset of participants from Experiment 1 who returned for further testing. While Experiments 1, 2 each utilized different participant samples, Experiment 3 involved a subset of participants from Experiment 1 who returned for further testing. Experiment 4 employed cTBS combined with fMRI, aiming to probe the causal role of the temporal deactivation mechanism in inhibition. All experiments employed a within‐subject, event‐related design, which was well‐suited to minimize order‐dependent confounds such as practice and fatigue effects. All the conditions in the experiment are presented randomly in sequence.

All experiments included both biological replicates (different participants) and technical measures to ensure reliability. Each participant completed multiple trials per condition, serving as technical replicates. Key findings were consistent across the different experimental paradigms and participant groups, demonstrating the robustness of the observed neural mechanisms.

#### 4.4.1 Experiment 1 (functional Magnetic Resonance Imaging, fMRI)

Both the Auditory and Visual experiments followed a within‐subject, event‐related design with four task conditions (NP, direct inhibition, distraction, and distancing). Each voice clip or picture was repeated twice to ensure sufficient trials (n = 50) for each task condition. The interval between the trials was randomly 1.5 s. All experimental trials were presented to each participant within a single, continuous functional run, lasting ≈60 min.

#### 4.4.2 Experiment 2 (Electroencephalogram, EEG)

Experiment 2 was the Auditory experiment. The experimental design for this study was identical to that of Experiment 1. Experiment 2.1 used 6‐s voice stimuli, while the complementary Experiments 2.2 and 2.3 employed 3 and 10‐s voice stimuli, respectively, to replicate the findings across varying stimulus lengths.

#### 4.4.3 Experiment 3 (fMRI)

##### Auditory

A total of 15 participants from Experiment 1‐Auditory revisited the experiment one week later. The task procedure and experimental paradigm were identical to those of Experiment 1, with the following exceptions: First, this experiment utilized 25 normal voice stimuli, randomly selected from the original 100 voice clips (from Experiment 1), along with 100 scrambled voice stimuli. Second, since participants had previously engaged in inhibiting normal voices in Experiment 1, this supplementary experiment instructed them either to concentrate on the normal voice (representing maximal attention) or to employ inhibition strategies while processing the scrambled voice (representing minimal attention or near‐complete inhibition). Third, trials with scrambled voices included only the “sensory” and “understand” questions. Although the “understand” question for scrambled stimuli may seem unnecessary, it serves a technical purpose in confirming that the scrambled voices convey no meaningful information, thereby validating the intended function of the stimuli.

The design of this experiment allowed for a within‐subject comparison between participants’ first‐visit conditions (NP‐normal, direct inhibition‐normal, distraction‐normal, distancing‐normal) and their second‐visit conditions (concentration‐normal, NP‐scramble, direct inhibition‐scramble, distraction‐scramble, distancing‐scramble), resulting in a 9‐task condition within‐subject design.

##### Visual

A total of 27 participants from Experiment 1‐Visual also revisited the experiment one week later. The task procedure remained identical to Experiment 1, with the following modifications: First, this experiment utilized 25 IAPS pictures, randomly selected from the original set of 100. Second, since participants had previously engaged in shielding pictures in Experiment 1, this supplementary experiment required them to close their eyes during the picture presentation period.

This design allowed for a within‐subject comparison of participants’ first‐visit conditions (NP, direct inhibition, distraction, distancing) and their second‐visit condition (close‐eye), resulting in a 5‐task condition within‐subject design.

#### 4.4.4 Experiment 4 (continuous theta burst stimulation, cTBS+fMRI)

To investigate the causal role of DLPFC in implementing temporal deactivation during inhibition, a counterbalanced crossover design was employed that controlled for time effects, practice effects, and test‐retest reliability. The DLPFC was selected as the TMS target based on extensive previous literature establishing it as the most critical brain region for inhibitory control.^[^ ^62^ ^]^ Specifically, prior studies have demonstrated that excitatory neuromodulation of the DLPFC enhances inhibitory control performance,^[^ ^63^ ^]^ while inhibitory neuromodulation impairs it,^[^ ^38^ ^]^ making this region an ideal candidate for investigating causal mechanisms underlying inhibitory processes.

Participants were randomly assigned to two groups (n = 18 each; Figure 5A):

Group 1 (cTBS‐first): cTBS → immediate fMRI → 40‐min break → recovery fMRI

Group 2 (cTBS‐second): baseline fMRI → 40‐min break → cTBS → immediate fMRI

This design allowed to compare neural activity immediately following cTBS (disrupted DLPFC function) with non‐disrupted states (baseline or recovered), while controlling for potential confounds through counterbalancing.

##### Experimental Procedure

The experimental task was identical to Experiment 1‐Auditory, with modifications to accommodate the cTBS protocol. Each fMRI session consisted of 100 trials (25 per condition: NP, direct inhibition, distraction, distancing), using the same voice stimuli across sessions to ensure comparability. The 40‐min interval between sessions was chosen based on established cTBS recovery times,^[^ ^64^ ^]^ allowing sufficient time for neural effects to diminish in Group 1 while avoiding excessive delays that might introduce additional confounds.

Participants were positioned comfortably beside the MRI scanner during cTBS administration to minimize movement between cTBS and scanning. Following cTBS, participants were immediately transferred to the scanner with minimal movement. All participants began the fMRI scan within 30–60 s after cTBS completion.

##### Target Region of Interest localization

The DLPFC region identified from Experiment 1 as key nodes in the executive control network was targeted. The target coordinate [−40, 2, 30] was derived from peak activation of DLPFC ROI in the conjunction analysis of three inhibition strategies > NP contrast. Individual T1‐weighted structural scans from Experiment 1 were co‐registered to MNI space, and the target site was identified on each participant's normalized brain.

##### cTBS Protocol

cTBS was delivered using a figure‐eight coil (70mm) connected to a magnetic stimulator (M‐100 Ultimate; Shenzhen Yingchi Technology Co., Ltd, Shenzhen, China). The stimulation site was accurately localized and continuously monitored using a frameless stereotactic neuronavigation system (Brainsight, Rogue Research, Montreal, Canada), providing real‐time feedback on coil position and orientation relative to the target ROI.

Individual resting motor thresholds (rMT) were determined from the left primary motor cortex (M1) using standard procedures. Surface EMG electrodes were placed over the right first dorsal interosseous muscle to record motor evoked potentials (MEPs). The motor hotspot was identified as the scalp location producing the largest and most consistent MEPs. The rMT was defined as the minimum stimulator intensity producing MEPs ≥ 50 µV in at least 5 of 10 consecutive trials.

The cTBS protocol followed standard parameters.^[^ ^64^, ^65^ ^]^ Stimulation intensity was set to 80% of individual rMT to minimize unintended spread beyond the target cortical site while ensuring effective neuromodulation.^[^ ^66^ ^]^ The protocol delivered 600 pulses in bursts of three pulses at 50 Hz, repeated every 200ms (5 Hz), over a total duration of 40 s. The coil was positioned over left DLPFC and oriented tangentially to the scalp with the handle pointing posteriorly at ≈45° from the midline (see Figure 5A). This unilateral approach simplified the protocol while maintaining effectiveness, as previous studies had demonstrated that unilateral DLPFC stimulation could modulate bilateral inhibitory control networks through transcallosal connections.

### 4.5 Data Recording and Statistical Analysis

Data are presented as mean ± standard deviation (SD) unless otherwise stated. The statistical significance threshold (alpha level) was set at *p* < 0.05 for all analyses. To enhance readability when reporting multiple statistical tests collectively, the notation p‐values were used to refer to plural p‐values. For instance, *p*‐values < 0.05 indicate that the *p*‐values for all tests under discussion were less than 0.05. All p‐values were corrected for multiple comparisons using the FDR method and were denoted with the subscript FDR (*p*(FDR) or *p*(FDR)‐values). Effect sizes are reported where appropriate (Cohen's *d* for t‐tests, η^2^ for ANOVAs). Statistical analyses were performed using R Statistical Software (v4.3.2) with the lme4 and simr packages, Python (v3.10) with the MNE (v1.7.1), scikit‐learn, and ruptures libraries, MATLAB (2020b) with SPM12 and custom scripts, and SPSS with the PROCESS macro.

#### 4.5.1 Behavioral Data

Behavioral data were recorded using E‐prime 3.0 (PST, Pittsburgh, USA). Data were analyzed using R Statistical Software (v4.3.2). Generalized (logistic) linear mixed‐effect models were constructed to examine trial‐by‐trial behavior. In the basic model, the within‐subject predictor “strategy type” had two levels: trials in which participants employed one of the “distancing,” “distraction,” or “direct inhibition” strategies were labeled as inhibition, whereas trials in which participants processed the stimuli naturally were labeled as NP. The NP condition served as the baseline. Cumulative trial number (centered and standardized) was also included to account for the time effect. For each trial, responses to the “sensory,” “understanding,” and accuracy on “objective” questions were coded as zero (negative response—did not hear/see, did not understand, or incorrect) or 1 (positive response—heard/saw, understood, or correct) and used as response variables in separate models. Participants’ responses to the subjective “sensory” question were converted into the sensory rate (*P*_sensed_), representing the probability of having heard/seen the voice/picture stimulus. Similarly, responses to the subjective “understand” question were converted into the understanding rate (*P*_understood_), indicating the probability of having understood the stimulus. Participants’ performance on the objective multiple‐choice quiz was converted into accuracy (*ACC*_objective_), representing the proportion of correct answers. Random effects include a random intercept for each participant, and a random slope of strategy type. Statistical representation of the basic model is as follows:

Response_ij_ = β_0_ +  β_1_ *strategy_ij_* +  β_2_ *trial* *number_ij_* + *u*_0*j*_ +  *u*_1*j*_ *strategy_ij_*,

where:

Response_ij_ is binary response in the i‐th trial of the j‐th individual participant;

β_0_ is the fixed intercept at the reference level, i.e., in NP;

β_1_ is the fixed effect coefficient for strategy, i.e., the difference between inhibition and NP;

β_2_ is the fixed effect coefficient for trial number (time);

*u*_0*j*_ is the subject‐specific random intercept;

*u*_1*j*_ is a subject‐specific random slope for strategy.

The basic model was applied to Experiment 1 and 2. In Experiment 4, building on the basic model, task number (dummy‐coded; 0 = task 1, 1 = task 2) as well as its interaction with strategy type were further included as fixed effect predictors.

Participants who completed the study twice—that is, those who participated first in Experiment 1 and then in Experiment 3—were included to explore factors across different visits. Three models were constructed:

1. The “Concentration” model for auditory experiment followed a 3 (*task condition*: concentration, NP, inhibition) within‐subject design. NP was used as the baseline to examine whether the concentration and the inhibition condition differed from NP when participants were presented with normally ordered stimuli;
2. The “Scramble” model for auditory experiment followed a 2 (*task condition*: NP, inhibition) × 2 (*stimuli type*: normal order, scrambled order) within‐subject design. Here, NP was used as the baseline for *task condition* and normal order as the baseline for *stimuli type* to investigate differences in performance across *task condition* and *stimuli type* combinations. For this model, both *P*_sensed_ and *P*_understood_​ were included as response variables. Additionally, a manipulation check was conducted to verify whether the scrambled stimuli effectively conveyed meaningless information. In this check, *P*_understood_ served as the response variable in a model that included stimulus type (normal order vs scrambled order) as a fixed effect. The stimulus manipulation was considered successful if participants exhibited significantly lower *P*_understood_​ for scrambled voices compared to normal voices;
3. The “Close‐eye” model for visual experiment followed a 3 (*task condition*: close‐eye, NP) within‐subject design. NP was used as the baseline to examine whether the close‐eye condition differed from NP.

#### 4.5.2 EEG data

Continuous EEG data were recorded using a 64‐channel active electrode system with an actiCHamp amplifier (BrainProducts GmbH, Gilching, Germany). The electrodes were mounted in an elastic cap (actiCAP) and arranged according to the international 10–20 system. The recording was conducted in a quiet, dimly lit, shielded chamber. There were no other electronic devices or noise sources in the laboratory that may affect the EEG records.

During the experiment, participants were seated comfortably in an adjustable chair. Visual stimuli (instructional cues and feedback) were presented on a 24‐inch LCD monitor positioned approximately 60 cm in front of the participant at eye level. Auditory stimuli were delivered via insert earphones.

The EEG signal was sampled at 1000 Hz and referenced online to the CPz electrode. Electrode impedances were maintained below 10 kΩ throughout the recording session to ensure high signal quality. Data were recorded without any online filters applied.

##### Preprocessing

All EEG data were preprocessed in Python using the MNE library version 1.7.1 (https://mne.tools/stable/index.html). Bad channels were first manually inspected and interpolated, then data were averaged to a common average and filtered using a 0.1 to 40 Hz bandpass filter (Butterworth order 3) and a 50 Hz notch filter. The data was divided into epochs, and each epoch covered from 200ms before the stimulus appeared to the end of the stimulus. To correct eyeblink artifacts, data were processed through MNE's independent component analysis (ICA). Components with blink artifacts were automatically detected by MNE‐ICALabel toolbox (https://mne.tools/mne‐icalabel/stable/index.html). Then autoreject version 0.4.2 was used, a Python‐based data‐driven method that learns the maximum peak‐to‐peak thresholds based on trials from each subject to interpolate the bad channels and reject epochs (https://autoreject.github.io/stable/index.html). Finally, the data were corrected within a window from −200 to 0 ms. All automated results were manually reviewed. All the automated processing has undergone visual inspection.

##### Traveling Wave Analysis

Traveling wave's propagation along seven midline electrodes was quantified, running from occipital to frontal regions (Oz, POz, Pz, CPz, Cz, FCz, and Fz) and STC to DLPFC axis (left: FT7, F7, AF7, AF3; right: FT8, F8, AF8, AF4). The electrodes selection was directed by Koessler et al.^[^ ^67^ ^]^ he signals from the electrodes on each axis were stacked to create 2D maps, with time and electrodes as axes. From each map, 2D fast Fourier transform (FFT) was computed: importantly, the power in the lower and upper quadrants quantifies the amplitude of FW (forward waves, from occipital to frontal electrodes) and BW (backward waves, from frontal to occipital) waves,^[^ ^68^ ^]^ respectively. For each frequency in the 4‐ to 40‐Hz range, the maximum values in both the upper and lower quadrants were considered, obtaining a spectrum for both BW and FW waves, respectively. A baseline value was obtained using average results of 1D‐FFT(one‐dimensional FFT) and converted FW and BW into dB units based on the baseline value. The baseline was the mean of the 1D‐FFT results. BW was calculated in the upper quadrant, and FW was calculated in the lower quadrant. For the theta (4–8Hz), alpha (8–14Hz), beta (14–30Hz), and gamma (30–40Hz) rhythms contained in 4–40Hz.

##### EEG Source Localization and Analysis

To estimate the cortical sources of the scalp‐recorded EEG activity, a source localization analysis was performed using the MNE‐Python library (https://mne.tools/stable/index.html). As individual structural MRI scans were not available for the participants, the analysis was conducted within a common template space using the fsaverage anatomical template provided by FreeSurfer. A forward solution was constructed using a three‐layer Boundary Element Model (BEM) derived from the fsaverage template and a source space with “oct6” resolution. EEG sensor locations were coregistered to the head model using a standard 10–20 system. For each participant and experimental condition, an inverse solution was computed using the Dynamic Statistical Parametric Mapping (dSPM) method. The inverse operator was regularized with a noise covariance matrix estimated from the pre‐stimulus baseline period. Key parameters for the inverse operator included a loose orientation constraint (loose = 0.2) and depth weighting (depth = 0.8) to reduce bias towards superficial sources. Finally, since individual source estimates were computed in the common fsaverage space, they were averaged across participants to generate grand‐average spatiotemporal maps for group‐level analysis.

To identify brain regions showing differential activation between conditions S and N, the difference in dSPM values (S‐N) was computed at each source location and time point across all participants. This resulted in a difference matrix with dimensions (n_subjects × n_vertices × n_times). An initial statistical threshold of *t* = 2.5 was applied to identify suprathreshold vertices. Adjacent vertices (as defined by the spatial connectivity matrix) exceeding this threshold were grouped into spatiotemporal clusters. The null distribution was generated through Monte Carlo permutation testing with 1000 random permutations of condition labels within each participant, preserving the within‐subject pairing structure. For each permutation, condition labels (IH vs NP) were randomly shuffled, and the same clustering procedure was applied. For each identified cluster, cluster‐level statistics was computed as the sum of *t*‐values within the cluster. The significance of each observed cluster was determined by comparing its cluster statistic against the null distribution of maximum cluster statistics obtained from the permutation procedure. Significant clusters were detected in the predefined ROIs (DLPFC, PPC, STC). Spatial cluster analysis was performed at selected time points (every 10th time point to reduce computational load) using permutation_cluster_1samp_test with spatial connectivity constraints. For vertices where spatial clustering failed, unconstrained vertex‐wise one‐sample t‐tests against zero for the difference scores (IH‐NP) were performed, followed by connected component analysis to identify spatiotemporally contiguous regions of activation. Cluster‐level *p*‐values were computed using the permutation‐based family‐wise error rate (FWER) correction. Clusters with *p* < 0.05 (corrected) were considered statistically significant, indicating reliable differences between conditions IH and NP.

##### Multivariate Pattern Analysis

MVPA on the preprocessed EEG data was applied using the MNE and scikit‐learn (https://scikit‐learn.org/stable). To achieve this, a classification algorithm was used based on SVM. For the decoding, the NP and inhibition labels were used as classes. The analysis tested whether the classifier could learn from distinct EEG patterns following every single trial to distinguish these pairs of conditions and characterize the process of the two classes on 3s dataset, 6s dataset, and 10s dataset. For the MVPA of the same dataset, one analysis was conducted on each of the electrodes selected under the guidance of whole brain electrodes and fMRI. K‐fold cross‐validation was used, whereby each participant's dataset was sorted into 5 folds; the classifier was trained on 4 folds and tested on the remaining one. To avoid selection biases, the entire analysis was repeated five times with random division between training and test data. To keep a balanced number of trials between conditions, trials were randomly selected and discarded when necessary (“undersampling”).^[^ ^69^ ^]^ To measure classification performance, we calculated the area‐under‐the‐curve (AUC) accuracy metric of the receiver operating characteristic (ROC), a measure derived from signal detection theory, insensitive to classifier bias.^[^ ^70^ ^]^

##### Change‐Point Detection

To objectively identify the single most significant shift in decoding performance within a specific temporal window of interest, a changepoint detection analysis was conducted on the grand‐average AUC curve. The Dynamic Programming algorithm, implemented in the ruptures Python library, was employed to locate the optimal breakpoint (https://centre‐borelli.github.io/ruptures‐docs/). This algorithm was configured to partition the signal segment within the time window into exactly two segments by identifying a single changepoint (n_bkps=1). The cost function for the segmentation was based on the L1 norm (model = “l1”), which is robust for detecting shifts in the mean of a time series. The resulting changepoint, therefore, represents the time point that most significantly partitions the data into two distinct periods of decoding accuracy, marking the moment of the most abrupt change in classifier performance within the specified interval.

#### 4.5.3 MRI Data

Brain images were collected using a 3T MR scanner (Siemens Trio). Functional images were collected using an EPI sequence (number of slices, 72; gap, 0.6 mm; slice thickness, 2.0 mm; TR, 1500ms; TE, 30ms; flip angle, 75°; voxel size, 2 mm × 2 mm × 2 mm; FOV, 192 mm × 192 mm). Structural images were acquired through 3D sagittal T1‐weighted MPRAGE (224 slices; TR, 1900ms; TE, 2.23ms; voxel size, 1.1 mm × 1.1 mm × 1.1 mm; flip angle, 8°; inversion time, 904 ms; FOV, 220 mm × 220 mm).

Preprocessing, subject‐level, group‐level, and the temporal analysis of brain activity and RSA were applied to Experiment 1 and 3. The MVPA was applied to Experiment 1. All fMRI analyses were conducted on the pre‐defined ROIs, unless otherwise mentioned.

##### Regions of Interest Definition

Given that inhibition involves top‐down processes wherein higher‐order cognitive regions exert control over sensory regions,^[^ ^11^ ^]^ the focus was placed on the DLPFC and PPC, which together constitute the executive control network.^[^ ^71^ ^]^ Additionally, sensory regions specific to each modality were included—namely, the STC for auditory processing^[^ ^72^, ^73^ ^]^ and the visual cortex (VC, including V1‐V3) for visual processing.^[^ ^74^ ^]^ Increased activation within the executive control network would thus serve as an indicator of the implementation and execution of inhibition strategies. Conversely, a decrease in STC or VC activation would indicate inhibition engagement, based on traditional models and empirical studies of attention, suggesting that activations in stimulus‐specific regions are reduced when participants ignore stimuli.^[^ ^15^, ^75^ ^]^ All ROIs were selected based on the anatomical definitions provided by the Automated Anatomical Labeling atlas 3 (AAL3).^[^ ^76^ ^]^ Specifically, the DLPFC was defined as Brodmann areas (BA) 9 and 46;^[^ ^77^ ^]^ the PPC as BA 5 and 7;^[^ ^78^ ^]^ the STC as BA 22;^[^ ^79^ ^]^ and the VC as BA 17‐19.^[^ ^80^, ^81^ ^]^ These ROIs were defined in MNI space, combined into a unified ROI mask specific to each experiment: for the auditory experiment, the ROIs included the DLPFC, PPC, and STC; for the visual experiment, the ROIs comprised the DLPFC, PPC, and VC. These unified ROI masks were then transformed into individual participant space using the inverse normalization parameters estimated during preprocessing. To investigate DMN, the core regions including mPFC (BA 10, 32), PCC (BA 23, 31), angular gyrus (BA 39), precuneus, and hippocampus were used (both regions were defined using AAL atlas due to that they are medial parts was not properly defined by BA).

##### Preprocessing and Subject‐Level Analyses

Images were preprocessed and analyzed using Matlab (2020b) and Statistical Parametric Mapping (SPM12; Wellcome Trust Centre for Neuroimaging, London, UK). The first ten volumes were discarded to account for signal equilibration and participants’ adaptation to scanning noise. Functional data were first corrected for geometric distortion with the SPM FieldMap toolbox, and slice‐time corrected and realigned for motion correction by registration to the mean image. Artifact detection was conducted using the Artifact Detection Tools (ART) software (https://www.nitrc.org/projects/artifact_detect); global mean intensity (> 2 standard deviations from mean image intensity for the entire scan) and motion (> 2 mm) outliers were identified and entered as regressors of no interest in the first‐level GLM.^[^ ^82^ ^]^ Then, functional images were co‐registered with the T1‐weighted 3D images, normalized to MNI space, and smoothed with an 8‐mm full‐width at half‐maximum isotropic Gaussian kernel. Out six motion estimates (three translations and three rotations) were regressed, one artifact (outlier scans) as identified by ART, and two physiological time series (cerebrospinal fluid and the white matter signals) with global signal regression.

For subject‐level analyses, GLMs were specified with four regressors for the four conditions (NP, direct inhibition, distraction, and distancing). The duration for each regressor was defined as the voice/picture exposure period (6 s) per trial. All regressors were convolved with the canonical hemodynamic response function in SPM12. Each normalized image was then high‐pass filtered using a cutoff time constant of 128 s. For each participant in Experiment 1, seven contrast images:^[^ ^1^ ^]^ NP,^[^ ^2^ ^]^ direct inhibition,^[^ ^3^ ^]^ distraction,^[^ ^4^ ^]^ distancing,^[^ ^5^ ^]^ inhibition,^[^ ^6^ ^]^ inhibition > NP, and^[^ ^7^ ^]^ inhibition < NP were generated. Contrasts 1‐4 were calculated against the implicit baseline as defined by SPM. To identify brain regions commonly activated across all inhibition strategies, a conjunction analysis of direct inhibition, distraction, and distancing conditions, producing the contrast 5 (inhibition) were performed. Contrast 6 represented inhibition‐related activation, obtained by the conjunction analysis of (direct inhibition > NP), (distraction > NP), and (distancing > NP). Conversely, contrast 7 represented inhibition‐related deactivation, derived from a conjunction analysis of (NP > direct inhibition), (NP > distraction), and (NP > distancing). For each conjunction analysis, the resulting statistical map identifies voxels where all included contrasts show an effect in the same direction at a specified threshold. Based on standard neuroimaging practices,^[^ ^83^ ^]^ an initial voxel‐level threshold of *p* < 0.001 (uncorrected) was applied to identify voxels of interest within each individual contrast before assessing the conjunction. The final group‐level activation maps were then corrected for multiple comparisons at the cluster level using a FDR corrected threshold of *p* < 0.05, with a minimum cluster extent of ten contiguous voxels.

For each participant in Experiment 3‐Auditory, besides the first‐visit contrast images (the above seven), ten additional contrast images:^[^ ^1^ ^]^ concentration,^[^ ^2^ ^]^ concentration > NP,^[^ ^3^ ^]^ concentration < NP,^[^ ^4^ ^]^ NP‐scramble,^[^ ^5^ ^]^ direct inhibition‐scramble,^[^ ^6^ ^]^ distraction‐scramble,^[^ ^7^ ^]^ distancing‐scramble,^[^ ^8^ ^]^ inhibition‐scramble,^[^ ^9^ ^]^ inhibition > NP‐scramble, and^[^ ^10^ ^]^ inhibition < NP‐scramble were generated. For each participant in Experiment 3‐Visual, one additional contrast image were generated:^[^ ^1^ ^]^ close‐eye.

These subject‐level contrast images were subsequently used as inputs for second‐level random‐effects analyses to assess group‐level effects. All quantitative analyses were performed on the full anatomical ROI masks.

##### Group‐Level Activity Analysis

In Experiment 1, one‐sample *t*‐tests on the inhibition > NP and inhibition < NP contrasts were performed to detect group‐level inhibition‐related brain activation and deactivation. The test was conducted for both the combined ROI (i.e., DLPFC, PPC, STC/VC) for the main analysis, and additionally, separate analyses were conducted for the sensory regions and DMN to examine whether observed sensory deactivation could be confounded with general DMN suppression during task engagement. Specifically, a Region (sensory cortex vs DMN hubs) × Condition (NP vs inhibition) interaction analysis were conducted to statistically dissociate sensory‐specific deactivation from general DMN suppression. Furthermore, to thoroughly examine both the similarities and differences across individual inhibition strategies, a flexible factorial design was implemented with the factor *strategy* (NP, direct inhibition, distraction, distancing) to assess whether each strategy differs in its pattern of activation and deactivation.

In Experiment 3, two models was developed: the “Concentration model” incorporated the first‐visit conditions (inhibition and NP) and the second‐visit condition (concentration). One‐sample *t*‐tests was conducted on the inhibition > NP, inhibition < NP, concentration > NP, concentration < NP contrasts respectively, to detect group‐level inhibition‐related or concentration‐related brain activation and deactivation, and pair‐sample *t*‐tests to compare the activation and deactivation differences between inhibition and concentration. The “Scramble model” combined the first‐visit conditions (inhibition‐normal and NP‐normal) with the second‐visit conditions (inhibition‐scramble and NP‐scramble). One‐sample *t*‐tests was conducted on the inhibition > NP‐normal, inhibition < NP‐normal, inhibition > NP‐scramble, inhibition < NP‐scramble contrasts respectively, to detect group‐level “active inhibition”‐related or “complete inhibition”‐related brain activation and deactivation, and pair‐sample *t*‐tests to compared the activation and deactivation differences between active inhibition and complete inhibition. In Experiment 4, for both inhibition > NP and NP > inhibition contrasts, one‐sample *t*‐tests was performed on the 0‐3s and 3‐6s respectively, and a pair‐sample *t*‐test to compared the differences between pre‐cTBS and post‐cTBS.

##### Activity Temporal Analysis

To investigate potential temporal dissociations in brain activity as a result of the inhibition manipulation, GLM analyses were conducted to compare neural responses across two time frames for the voice/picture stimuli. Specifically, the models examined brain activity during the 0‐3 s, and 3‐6 s time periods following the onset of the 6‐s voice/picture. For both inhibition > NP and NP > inhibition contrasts, Experiment 1 performed one‐sample *t*‐tests on the 0‐3s and 3‐6s respectively, and a pair‐sample *t*‐test to compare the temporal difference. Similarly, analyses were performed on the combined ROI for the primary analysis, and additionally, separate analyses were conducted for the sensory regions and core DMN hubs to examine whether sensory deactivation was distinct from general DMN suppression during task engagement. Specifically, a Region (sensory cortex vs DMN hubs) × Condition (NP vs inhibition) × Time (first 3s vs last 3s) interaction analysis was implemented to statistically differentiate temporal patterns of sensory‐specific deactivation from DMN‐related suppression. Furthermore, to comprehensively explore both similarities and differences among individual inhibition strategies over time, an additional flexible factorial analysis with factors of Strategy (NP, direct inhibition, distraction, distancing) and Time (first 3s, last 3s) was conducted to identify whether each strategy exhibits distinct temporal dynamics in activation and deactivation patterns.

In Experiment 3, the “concentration model” used a 3 (*task conditions*: concentration, inhibition, and NP) × 2 (*time*: pre‐3s, post‐3s) flexible factorial design to test whether concentration and inhibition may differ in the activation and deactivation across different time periods. The “scramble model” used a 2 (*task conditions*: inhibition, NP) × 2 (*time*: pre‐3s, post‐3s) × 2 (*voice type*: normal, scramble) flexible factorial design to test inhibition‐normal and inhibition‐scramble may differ in the activation and deactivation across different time periods. In Experiment 4, for both inhibition > NP and NP > inhibition contrasts, a 2 (*TMS status*: pre‐cTBS, post‐cTBS) × 2 (*time*: pre‐3s, post‐3s) flexible factorial design was used to test whether pre‐cTBS and post‐cTBS may differ in the activation and deactivation across different time periods.

##### Beta Value Extraction and Visualization

For the brain activity analysis, a voxel‐wise analysis was conducted within the unified ROI, and the surviving voxels (after performing FDR correction across all the voxels within the ROI) are displayed on the brain activation maps. To further illustrate the results, the averaged BOLD signals (parameter estimates) were extracted from all voxels within each individual ROI for each participant using the MarsBaR toolbox (https://marsbar‐toolbox.github.io). These averaged parameter estimates were then subjected to *post‐hoc* statistical tests and visualization.

##### Decoding Analysis

The analysis was conducted using the CoSMoMVPA toolbox (https://www.cosmomvpa.org) and LIBSVM (https://www.csie.ntu.edu.tw/~cjlin/libsvm). The analysis included three components: 1) a unimodal MVPA to assess the discriminability of brain activity patterns between inhibition and NP conditions within the same sensory modality, 2) a feature MVPA analysis to assess how inhibition affects the discriminability of information features (high vs low valence, arousal, information load, and processing difficulty) in neural activity patterns, and 3) a cross‐modal decoding analysis to test whether inhibition‐related neural patterns generalize across auditory and visual modalities. Since the number of IH conditions is greater than that of NP, when conducting MVPA analysis, downsampling was used to randomly discard the excessive conditions to ensure quantitative balance. All MVPA analyses were conducted on all voxels within the predefined ROIs. To rigorously assess classification performance, for each analysis AUC scores were also computed, where an AUC of 0.5 indicates chance‐level performance and 1.0 indicates perfect classification. Statistical significance of AUC values was determined using one‐sample t‐tests against chance level (0.5).^[^ ^36^ ^]^

###### Data Preparation

Following data preprocessing and GLM analysis, beta values were generated corresponding to each condition (inhibition or NP) across three time periods: 0–6, 0–3, and 3–6 s post‐stimulus onset. However, the initially generated beta values were not suitable for MVPA, which requires trial‐specific data. Therefore, beta values were restored for each single trial (https://github.com/ritcheym/fmri_misc/blob/master/generate_spm_singletrial.m). These restored single‐trial beta values were then used in the following MVPA analyses.

###### Unimodal MVPA

The model's ability was evaluated to discriminate between inhibition and NP conditions within each modality (using data from Experiment 1′s auditory and visual tasks). Higher decoding accuracy indicates more distinct neural representations compared to the chance level (0.5). The previously defined ROIs (DLPFC, PPC, STC/VC) were focused and used the beta values across all voxels within each ROI to decode between NP and inhibition. A leave‐one‐run‐out cross‐validation procedure was employed to validate the classifier's ability to distinguish between conditions. This is to determine whether there are stable and decodable neural patterns within an individual to distinguish different conditions. In each classification iteration, the training set consisted of data from all runs except one (40 trials: 20 trials per condition), and the testing set included data from the left‐out run (10 trials: 5 trials per condition). To reduce variability and enhance the reliability of the results, 100 bootstrap iterations were performed with random resampling of trials. This method first achieved stable decoding performance at the individual level.

In the preliminary analyses, classification performance extremes (e.g., accuracies approaching 0% for NP condition and 100% for inhibition condition) were observed, suggesting potential model overfitting to noise or irrelevant patterns in the high‐dimensional data. This phenomenon, well‐documented in neuroimaging literature, often occurs due to the “curse of dimensionality”, where the large number of voxels relative to the limited training samples leads to unstable or unreliable model performance.^[^ ^84^ ^]^ To address this issue and improve generalizability, Principal Component Analysis (PCA) was applied to the training data before fitting the SVM classifier, effectively reducing dimensionality while preserving meaningful signal variation.^[^ ^85^ ^]^

###### Feature MVPA

The model's ability was evaluated to discriminate among the following four conditions: high and low intensity of information features under the inhibition state, and high and low intensity of information features under the NP state (using data from Experiment 1′s Visual task). The analyzed features included valence, arousal, information load, and processing difficulty, which were derived from ratings by an independent, demographically‐matched sample (see “Methods‐Experimental materials” for details). To ensure balanced trial numbers across conditions, features were binarized based on median splits: ratings above the median were classified as high intensity, while those below were classified as low intensity. Higher decoding accuracy indicates more distinct neural representations compared to the chance level (0.25). To facilitate holistic comparisons across different conditions, the combined ROI, which included the DLPFC, PPC, and VC was focused. Beta values from all voxels within this ROI were used to decode among the four conditions. A leave‐one‐run‐out cross‐validation procedure was employed to validate the classifier's ability to distinguish between conditions. In each classification iteration, the training set consisted of data from all runs except one (16 trials: 8 trials per condition), and the testing set included data from the left‐out run (4 trials: 2 trials per condition). The bootstrap iterations and PCA settings were identical to those used in the unimodal MVPA.

###### Cross‐Modal Decoding Analysis

The model's ability was evaluated to generalize the inhibition or NP conditions across modalities (Experiment 1, auditory and visual tasks), with higher accuracy indicating greater generalization of neural representations between modalities. To facilitate cross‐modal analysis, a frontoparietal ROI was defined by combining the regions (DLPFC and PPC) that were consistently involved in both Auditory and Visual tasks.

The cross‐modal decoding analysis was conducted under two configurations: 1) auditory modality as training set, visual modality as testing set, and 2) visual modality as training set, auditory modality as testing set. Unlike the unimodal MVPA using the leave‐one‐run‐out method, for the cross‐modal decoding analysis, the inherent cross‐modal testing procedure served as validation. The selection of visual trials matched the sample sizes of the auditory dataset and ensured a balanced number of trials for both NP and inhibition conditions. The bootstrap iteration settings were identical to those used in the unimodal MVPA. Features extracted via PCA from one modality training data to extract shared, modality‐invariant features, which were then applied to another modality testing data for classification.

###### Statistical Analysis

For both unimodal and cross‐modal decoding analysis, one‐sample *t*‐tests were conducted to determine whether the classification accuracy for each condition significantly exceeded the chance level (0.5). For the feature MVPA, one‐sample t‐tests were conducted to determine whether the classification accuracy for each condition significantly exceeded the chance level (0.25). Paired‐ or two‐sample *t*‐tests were performed to compare the accuracy differences between the inhibition and NP conditions within the same modality (unimodal MVPA) or between the two modalities (cross‐modal decoding analysis). Effect sizes for the *t*‐tests were calculated using *Cohen's d*. For the feature MVPA, a repeated‐measures ANOVA with *task condition* (NP, inhibition) and *feature intensity* (high, low) as within‐subject factors was used to determine whether inhibition affects the discriminability of information features. Effect sizes were calculated using η^2^.

##### Representational Similarity Analysis

RSA enables direct comparisons across different information types (e.g., neural activity, computational models, or behavioral data) by transforming complex, multi‐dimensional data into comparable geometric structures. Specifically, RSA constructs representational distances (e.g., Euclidean distances or correlation‐based measures) to quantify similarity within a representational space.^[^ ^86^ ^]^ This approach makes RSA particularly suitable for the research questions:^[^ ^1^ ^]^ whether neural representations are consistent or distinct across different experimental conditions, and^[^ ^2^ ^]^ whether specific stimulus features are represented in neural activity patterns.

The analysis was conducted using MATLAB, SPM, The Decoding Toolbox (https://sites.google.com/site/tdtdecodingtoolbox), and custom scripts. It included two main components: a feature‐neural RSA, examining how neural activity patterns represent specific stimulus features, and a neural‐neural RSA, assessing the similarity of neural representations across experimental conditions. To obtain a holistic representation and facilitate comparisons, RSA was performed within combined ROIs for each modality (Auditory: DLPFC, PPC, STC; Visual: DLPFC, PPC, VC).

###### Data Preparation

The analysis used the single‐trial beta values corresponding to each condition (e.g., inhibition or NP). Pairwise Pearson correlation coefficients between all single‐trial beta values of the ROI across the relevant conditions were computed, resulting in a representational dissimilarity matrix (RDM) for each participant. It reflects the dissimilarity of neural patterns between trials, where lower correlation indicates greater dissimilarity. To achieve a group‐level representation, we averaged the RDMs across all participants, producing an average RDM. This average RDM was compared with a conceptual model RDM—an idealized binary classification model with values of 0 and 1 representing expected dissimilarities between conditions—using Spearman's rank correlation coefficient. This comparison assessed how well the neural patterns aligned with the predicted patterns based on the conceptual model, thereby evaluating the ROI's ability to distinguish between NP and inhibition conditions. A permutation test with 5000 iterations was conducted to determine the statistical significance of these correlations. Finally, the RDMs were transformed into RSMs by subtracting each value in the RDM from 1 (i.e., RSM = 1 − RDM). This transformation converts dissimilarity measures into similarity measures, facilitating direct comparison of neural patterns.

###### Feature‐Neural RSA

The analysis was only performed on the 0‐6 s time window to shrink the analysis and presentation load. This analysis was conducted in the Auditory and Visual experiments. Neural RDMs were first created separately for the individual inhibition and NP conditions and converted to RSMs. Next, feature RDMs were constructed based on four subjective perceptual dimensions: valence, arousal, information load, and processing difficulty. These perceptual dimensions were derived from ratings provided by an independent sample that was demographically matched to the main study participants (further details can be found in the “Experimental materials” section). For each perceptual dimension, the absolute difference between the ratings of each pair of stimuli (for example, the valence RDM(i,j) = |valence_values(i) − valence_values(j)|) were calculated. This produced a symmetric feature RDM for each dimension, where larger values represent greater dissimilarity between stimuli on that perceptual feature. The created feature RDMs were then converted to RSMs.

It was ensured that the neural RSMs and feature RSMs were properly aligned, so that each element (i, j) in the matrices corresponded to the same pair of stimuli. The Spearman correlation coefficient was then calculated between each feature RSM and the corresponding neural RSM. The Spearman correlation was chosen for its robustness to non‐parametric data and its ability to capture monotonic relationships. This correlation quantified the degree to which neural similarity patterns reflected the similarities in subjective perceptual features, providing insights into how well neural representations align with participants’ perceptual experiences.

To quantify inhibition‐induced decoupling, neural‐feature RSM correlations between inhibition and NP conditions were compared using Steiger's *z*‐tests,^[^ ^87^ ^]^ which account for the dependency between correlations calculated on the same sample.

###### Neural‐Neural RSA

The analysis was performed across three time windows: 0–6, 0–3, and 3–6 s post‐stimulus onset. For the “Concentration” model, RSMs were generated for the concentration versus NP conditions and separately for the inhibition versus NP conditions. This resulted in two RSMs. Pearson correlation coefficients were calculated between these two RSMs. This analysis evaluates whether the neural patterns associated with the concentration versus NP contrast were similar to those associated with the inhibition versus NP contrast.

For the “Scramble” model, RSMs were generated for the inhibition versus NP (normal) conditions and separately for the inhibition versus NP (scramble) conditions. This resulted in two RSMs for each time window. The Pearson correlation coefficients were calculated between these two RSMs. This analysis evaluates whether the neural patterns associated with the inhibition versus NP (normal) contrast were similar to those associated with the inhibition versus NP (scramble) contrast.

For the “Close‐eye” model, RSMs were generated for the close‐eye versus NP conditions and separately for the inhibition versus NP conditions. This resulted in two RSMs for each time window. The Pearson correlation coefficients were calculated between these two RSMs. This analysis evaluates whether the neural patterns associated with the close‐eye versus NP contrast were similar to those associated with the inhibition versus NP contrast.

##### Dynamic Causal Modeling

To investigate the effective connectivity and task‐induced modulation between key brain regions, DCM for fMRI was employed using SPM.^[^ ^88^ ^]^ The analysis was conducted separately for the visual and auditory modality experiments.

###### Model Specification

For each modality (visual and auditory), a network consisting of three regions of interest was defined: the DLPFC, the corresponding primary sensory cortex (VC for visual modality or STC for auditory modality), and the DMN. Within this three‐node architecture, a set of ten competing models was specified to test different hypotheses about how the inhibition task modulated network connections.

###### Endogenous Connectivity (A‐matrix)

All models were specified with full, bidirectional intrinsic (task‐independent) connections between all three nodes (DLPFC↔VC/STC, DLPFC↔DMN, VC/STC↔DMN). **Driving Inputs (C‐matrix)**: The main effect of stimulus presentation was set as a driving input to the respective sensory cortex node (VC for visual modality or STC for auditory modality).

###### Modulatory Effects (B‐matrix, see Figure S4 C, Supporting Information)

The models differed critically in their assumptions about which connections were modulated by the inhibitory condition: M1 (Full connectivity model): All extrinsic connections among DLPFC, sensory cortex, and DMN were subject to task‐induced modulation, excluding self‐connections. M2 (DLPFC‐to‐sensory modulation model): Only the top‐down connection from DLPFC to the sensory cortex was modulated. M3 (DLPFC‐to‐DMN modulation model): Only the top‐down connection from DLPFC to the DMN was modulated. M4 (DLPFC dual‐target modulation model): Both top‐down connections from DLPFC to sensory cortex and from DLPFC to DMN were modulated. M5 (sensory cortex‐to‐DLPFC modulation model): Only the bottom‐up connection from the sensory cortex to DLPFC was modulated. M6 (sensory cortex‐to‐DMN modulation model): Only the bottom‐up connection from the sensory cortex to DMN was modulated. M7 (sensory cortex dual‐target modulation model): Both bottom‐up connections from the sensory cortex to the DLPFC and from the sensory cortex to DMN were modulated. M8 (DMN‐to‐DLPFC modulation model): Only the bottom‐up connection from DMN to DLPFC was modulated. M9 (DMN‐to‐sensory cortex modulation model): Only the bottom‐up connection from DMN to sensory cortex was modulated. M10 (DMN dual‐target modulation model): Both bottom‐up connections from DMN to DLPFC and from DMN to sensory cortex were modulated. In the above models, M1 is a fully connected model. Due to the possible complex overall network changes during the inhibition process.^[^ ^88^, ^89^ ^]^ M2‐M4 is a top‐down model that only includes DLPFC control from top to bottom.^[^ ^90^ ^]^ M5‐M10 only contains bottom‐up connections, only connections from the DMN or sensory areas to other brain regions.^[^ ^91^, ^92^ ^]^

###### Model Estimation and Group‐Level Analysis

After the models were specified and estimated for each individual subject, group‐level analyses were performed. Model comparison was conducted using a random‐effects (RFX) Bayesian Model Selection (BMS) procedure to determine the optimal model for each modality, as indicated by the highest exceedance probability (EP). In addition to EP, each model's free energy, Bayesian Omnibus Risk (BOR), and posterior exceedance probability (PEP;^[^ ^93^ ^]^) were reported.

To assess the strength and direction of specific connections from the winning model, the Parametric Empirical Bayes (PEB) framework was used.^[^ ^94^ ^]^ This allowed for group‐level inference on the parameters for both endogenous (A‐matrix) and modulatory (B‐matrix) connections. A connection was considered statistically significant if its 95% posterior credible interval (CI) did not contain zero.

##### Multiple Comparison Correction

Brain activation was reported for clusters containing more than 10 voxels if they survived an FDR correction at *p* < 0.05. All *p*‐values were corrected for multiple comparisons using the FDR method and are denoted with the subscript FDR (*p*(FDR) or *p*(FDR)‐values).

##### Neural‐Behavioral Correlation Analysis

To investigate the relationship between neural and behavioral effects of inhibition, Pearson correlations were performed. For each participant, inhibition‐specific changes were calculated by taking the difference in behavioral performance (Δ*P*_sensed_, Δ*P*_understood_, and Δ*ACC*_objective_, calculated as IH minus NP) and in fMRI activation (Δ*β*, calculated as *β*_IH_ minus *β*_NP_ for mean beta values within each ROI). This subtraction method was employed to isolate the modulatory effect of inhibition relative to each individual's baseline processing during the NP condition, thereby aiming to account for stable inter‐individual differences in overall task engagement or neural responsiveness observed during the NP state. These inhibition‐related neural (Δ*β*) and behavioral difference scores were then correlated across participants for the 0–6, 0–3, and 3–6s time windows. Statistical significance was set at *p*(FDR) < 0.05.

##### Mediation Analysis

A mediation analysis was conducted using the PROCESS macro (Model 4;^[^ ^95^ ^]^) in SPSS. The effect of cTBS (X, coded 0 for pre‐stimulation, −1 for post‐stimulation) on behavioral inhibitory performance (Y) was tested for mediation by changes in temporal deactivation (M) of sensory cortices. Significance of indirect effects was tested using bias‐corrected bootstrapping with 5000 bootstrap samples and 95% confidence intervals (CI). An indirect effect was considered statistically significant if the 95% CI did not include zero.

## Ethical Statement

Experiments 1‐4 were approved by the Ethical Committee at the School of Psychology, Shenzhen University (Approval No. SZU_PSY_2024_067).

## Conflict of Interest

The authors declare no conflict of interest.

## Author Contributions

Z.H. conceived the study and designed the experiments; Y. Z. conducted the research; Z. H., R. E., Y. D., Z. F., Y. Z. performed data analyses; Z. H. wrote the initial draft of the manuscript; N. M., R. E., and B. S. edited and reviewed the final manuscript. Z. H. acquired funding; B. S. and R. E. supervised the project.

## Supporting information



Supporting Information

## Data Availability

The data that support the findings of this study are available from the corresponding author upon reasonable request.
