# Supplementary material for: Dynamic Neural Deactivation Bridges Direct and Competitive Inhibition Processes
Source: Adv Sci (Weinh). 2025 Aug 26;12(43):e06833. doi: 10.1002/advs.202506833 (PMC12631827; doi:10.1002/advs.202506833)
Supplement: Supplementary file 1 — Supporting Information [file ADVS-12-e06833-s001.docx]

**Supporting Information**

**Dynamic neural deactivation bridges direct and competitive inhibition processes**

*Zhenhong He^1^, Yifan Du^4^, Ziqi Fu^5^, Youcun Zheng^6^, Nils Muhlert^2^, Barbara Sahakian^3^, Rebecca Elliott^2^*

**Supporting Information Text**

Text 1. Inhibition strategy instructions

**Concentration:** Voice/Picture will be presented shortly. Please concentration carefully and attentively to the sounds/pictures that follow.

**Natural processing** (*1*): Voice/Picture will be presented shortly. Please relax and respond naturally.

**Direct inhibition** (*2*): Voice/Picture will be presented shortly. Please do not think about any thoughts, feelings, or sensations you might have about this voice/picture. You should not think about any experiences mentioned in the voice/picture. Try to avoid thinking about or feeling anything to do with this voice/picture or the entire process. Even if you start to notice the voice/picture, exclude those thoughts from your mind and stop thinking about them. Not thinking about the voice/picture is your primary task. It is important that you do not think about the voice/picture, or anything related to this task.

**Distraction** (*2*): Voice/Picture will be presented shortly. Your task is to distract your attention away from any thoughts, feelings, or sensations about the voice/picture. You should do this by thinking about the different rooms in your home. Imagine your home, room by room, as much as you can. Picture the colors of the walls, furniture, photos on the wall, making each room as vivid as possible. Imagine yourself being there, occupy your thoughts, and imagine as many images, scenes, sounds, and activities as you can. As vividly and in as much detail as possible. Even if your thoughts drift elsewhere, bring them immediately back to thoughts about your home. Forming a mental image of your home is your primary task. It is important that you continue imagining your home in vivid detail.

**Distancing** (*3*): Voice/Picture will be presented shortly. Imagine your soul leaving your body and observing everything objectively. Please take a third-person perspective of standing aloof from this matter, and observe it like a bystander.

Text 2. Validation of experimental materials

To facilitate direct comparisons between the ratings of stimuli across the four task conditions—in terms of valence, arousal, processing difficulty, and information load—we conducted mixed-effects ANOVAs, treating participants as random effects. For the auditory experiment, the assigned task condition (NP, direct inhibition, distraction, distancing) and voice duration (3seconds, 6 seconds, and 10 seconds) were included as within-subject factors. For the visual experiment, the assigned task condition was included as the within-subject factor.

For the auditory experiment, an independent sample of 28 college students (16 females; 20.04 ± 1.77, *M* ± *SD*), demographically matched to the main study participants, rated the stimuli using nine-point scales. Valence was rated from 1 (most negative) to 9 (most positive); arousal from 1 (least arousing) to 9 (most arousing); information load from 1 (lowest) to 9 (highest); and processing difficulty from 1 (least difficult) to 9 (most difficult). Results revealed no significant effects of task condition on any dimension for either 3-second [valence: *F*(3, 81) = 0.65, *p* = 0.588, *η²* = 0.018; arousal: *F*(3, 81) = 0.31, *p* = 0.818, *η²* = 0.009; information load: *F*(3, 81) = 0.04, p = 0.988, *η²* = 0.001; processing difficulty: *F*(3, 81) = 0.26, *p* = 0.857, *η²* = 0.007], 6-second [valence: *F*(3, 81) = 0.55, *p* = 0.647, *η²* = 0.015; arousal: *F*(3, 81) = 1.24, *p* = 0.298, *η²* = 0.034; information load: *F*(3, 81) = 0.46, *p* = 0.708, *η²* = 0.013; processing difficulty: *F*(3, 81) = 1.02, *p* = 0.388, *η²* = 0.028], or 10-second [valence: *F*(3, 81) = 0.36, *p* = 0.781, *η²* = 0.010; arousal: *F*(3, 81) = 1.53, p = 0.210, *η²* = 0.041; information load: *F*(3, 81) = 0.03, *p* = 0.994, *η²* = 0.001; processing difficulty: *F*(3, 81) = 1.59, *p* = 0.197, *η²* = 0.043] clips. Nevertheless, a main difference in all dimension ratings was found across the 3-, 6-, and 10- second duration stimulus (valence: 3-second < 6-second < 10-second, *F*(2, 297) = 68.258, *p* < 0.001, *η²* = 0.31; arousal: 3-second > 6-second > 10-second, *F*(2, 297) = 68.26, *p* < 0.001, *η²* = 0.27; information load: 3-second < 6-second < 10-second, *F*(2, 297) = 66.57, *p* < 0.001, *η²* = 0.31; processing difficulty: 3-second < 6-second < 10-second, *F*(2, 297) = 29.94, *p* < 0.001, *η²* = 0.17).

For the Visual experiment, an independent sample of 30 college students (15 females; 21.77 ± 2.01), demographically matched to the main study participants, rated the stimuli using nine-point scales (including valence, arousal, information load, and processing difficulty). Results revealed no significant effects of task condition on any dimension [valence: *F*(3, 87) = 1.51, *p* = 0.219, *η²* = 0.050; arousal: *F*(3, 87) = 0.44, *p* = 0.728, *η²* = 0.010; information load: *F*(3, 87) = 0.053, *p* = 0.984, *η²* = 0.001; processing difficulty: *F*(3, 87) = 0.217, *p* = 0.884, *η²* = 0.010]

All word texts of the video clips, the ID of the IAPS pictures, quizzes, descriptive statistics for average ratings as well as average rating for each individual stimulus can be found in **Data S1**.

**Data S1. (separate file) All word texts of the video clips, the IDs of the IAPS pictures, quizzes, and the average rating for each individual stimulus.**

Text 3. Detailed behavioral results

Descriptive statistics (*M ± SD*) are shown in **Table S1**. Demographic information of participants are shown in**Table S21**.

**Experiment 1**

**Auditory:** Compared to NP, inhibition did not produce in different *P*_sensed_ compared to NP [*log-odds* = -1.2, *z* = -1.65, *p* = 0.099, *d* = -0.66, 95% *CI* = (-2.64, 0.23)], but resulted in lower *P*_understood_ [*log-odds* = -1.23, *z* = -4.15*, p* < 0.001*, d* = -0.68, 95% *CI* = (-1.81, -0.65)] and lower *ACC*_objective_ [*log-odds* = -0.17, *z* = -2.1, *p =* 0.036, *d* = -0.09, 95% *CI* = (-0.33, -0.01); **Figure 2B**].

**Visual:** Compared to NP, inhibition resulted in lower *P*_sensed_ [*log-odds* = -0.88*, z* = -2.38*, p* = 0.017*, d* = -0.48, 95% *CI* = (-1.59, -0.16)], lower *P*_understood_ [*log-odds* = -1.48, *z* = -7.73, *p* < 0.001, *d* = -0.82, 95% *CI* = (-1.86, -1.11)] and lower *ACC*_objective_ [*log-odds* = -0.67, *z* = -7.58, *p* < 0.001, *d* = -0.37, 95% *CI =* (-0.84, -0.49); **Figure 2B**].

**Experiment 2**

**EXP 2.1 (6-second duration stimuli):** Compared to NP, inhibition did not significantly affect the *P*_sensed_ [*log-odds* = 0.57, *z* = 1.73, *p* = 0.084, *d* = 0.32, 95% *CI* = (-0.08, 1.23)], *P*_understood_ [*log-odds* = -0.24, *z* = -0.59, *p* = 0.554, *d* = -0.13, 95% *CI* = (-1.04, 0.56)], and reduced the *ACC*_objective_ [*log-odds* = -0.18 , *z* = -2.15, *p* = 0.031 , *d =* -0.1, 95% *CI* = (-0.35, -0.02); **Figure S1B**].

**EXP 2.2 (3-second duration stimuli):** Compared to NP, inhibition did not significantly affect *P*_sensed_ [*log-odds* = 0.38, *z* = 0.6, *p* = 0.547, *d* = 0.21, *95% CI* = (-0.85, 1.6)], *P*_understood_ [*log-odds =* -0.79*, z =* -1.44, *p* = 0.149, *d* = -0.43, 95% *CI* = (-1.85, 0.28)], but decreased *ACC*_objective_ [*log-odds* = -0.23, *z* = -3.35, *p* < 0.001, *d* = -0.13, 95% *CI* = (-0.36, -0.09); **Figure S1B**].

**EXP 2.3 (10-second duration stimuli):** Compared to NP, inhibition did not significantly affect *P*_sensed_ [*log-odds* = -0.57, *z* = -0.98, *p* = 0.329, *d* = -0.32, 95% *CI* = (-1.72, -0.58)], lower *P*_understood_ [*log-odds* = -1.4, *z* = -5.5, *p* < 0.001, *d* = -0.77, 95% *CI* = (-1.89, -0.9)], as well as lower *ACC*_objective_ [*log-odds* = -0.6, *z* = -4.47, *p* < 0.001, *d* = -0.33, 95% *CI* = (-0.86, -0.34); **Figure S1B**].

**Experiment 3**

**“Concentration” model for Auditory:** Compared to NP, neither inhibition [*log-odds* = 3.53, *z* = 1.15, *p* = 0.251, *d* = 1.95, 95% *CI* = (-2.5, 9.56)] or concentration [*log-odds* = 2.53, *z* = 0.69, *p* = 0.489, *d* = 1.4, 95% *CI* = (-4.65, 9.72)] differed in *P*_sensed_. Concentration did not yield a significant difference in *P*_understood_ compared to NP [*log-odds* = 0.55, *z* = 0.88, *p* = 0.378, *d* = 0.3, 95% *CI* = (-0.67, 1.76)], while inhibition significantly reduced *P*_understood_ [*log-odds* = -0.99, *z* = -2.01, *p* = 0.045, *d* = -0.55, 95% *CI* = (-1.96, -0.03)]. The difference in *ACC*_objective_ between concentration and NP condition was not significant [*log-odds* = 0.21, *z* = 0.31, *p* = 0.401, *d* = 0.17, 95% *CI* = (-0.42, 1.05)], while inhibition showed significantly lower *ACC*_objective_ compared to NP [*log-odds* = -0.68, *z* = -3.16, *p* = 0.002, *d* = -0.37, 95% *CI* = (-1.1, -0.26); **Figure S7A**].

**“Scramble” model for Auditory:** Compared to normal voice, scramble voice did not influence *P*_sensed_ [*log-odds* = -0.27, *z =* -0.29*,* p = 0.769, *d* = -0.15, 95% *CI* = (-2.08, 1.54)], but it resulted in lower *P*_understood_ [*log-odds* = -4.49, *z =* -11.4*, p* < 0.001, *d* = -2.47, 95% CI = (-5.26, -3.71)], demonstrating that the scrambled stimuli conveyed meaningless information (**Figure S7A**).

In normal voices, inhibition did not entail different *P*_sensed_ [*log-odds* = 0.85, *z =* 0.83*,* p = 0.405, *d* = 0.47, 95% *CI* = (-1.15, 2.84), but it did result in lower *P*_understood_ [*log-odds* = -1.16, *z =* -3.57*,* p < 0.001, *d* = -0.64, 95% *CI* = (-1.8, -0.52)] as compared to NP*.* In scrambled voices, inhibition did not produce significant differences in *P*_sensed_ [*log-odds* = -0.17, *z* = -0.17*, p* = 0.864, *d* = -0.09, 95% *CI* = (-2.07, 1.74)], but higher *P*_understood_ [*log-odds* = 0.92, *z* = 2.21*, p* = 0.027, *d* = 0.51, 95% CI = (0.11, 1.74)] compared to NP. It should be noted that the effect of inhibition on *P*_understood_ in the scrambled voice condition is not interpretable, as the scrambled stimuli lack semantic content. These findings suggest that when information cannot be received—such as with scrambled voices devoid of meaningful content—active inhibition is ineffective (**Figure S7A**).

**“Close-eye” model for Visual:** Compared to NP, close-eye led to a decrease in all of *P*_sensed_ (*log-odds* = -3.63, *z* = -6.57*, p* < 0.001*, d* = -2, *95% CI* = [-4.71, -2.55]), *P*_understood_ (*log-odds* = -1.78, *z* = -6.64*, p* < 0.001*, d* = -0.98, *95% CI* = [-2.3, -1.25]), and *ACC*_objective_ (*log-odds* = -1.32, *z* = -4.72*, p* < 0.001*, d* = -0.73, *95% CI* = [-1.87, -0.77]), demonstrating that the “close-eye” instruction was effective (**Figure S7A**).

**Experiment 4**

In NP, post-cTBS session and pre-cTBS session did not differ in *P*_sensed_ [*log-odds* = -1.12, *z* = -1.37, *p* = 0.172, *d* = -0.62, 95% *CI* = (-2.73, 0.49)], *P*_understood_ [*log-odds* = -0.26, *z* = -0.86, *p* = 0.391, d = -0.14, 95% CI = (-0.84, 0.33)], or *ACC*_objective_ in Task 1 [*log-odds* = 0.24, *z* = 1.83, *p* = 0.067, *d* = 0.13, *95% CI* = (-0.02, 0.5)]. Compare to NP, inhibition did not influence *P*_sensed_ [*log-odds* = -1.71, *z* = -1.39, *p* = 0.165, *d* = -0.94, 95% *CI* = (-4.11, 0.7)], impaired *P*_understood_ [*log-odds* = -1.55, *z* = -4.6, *p* < 0.001, *d* = -0.85, 95% *CI* = (-2.21, -0.89)], and impaired *ACC*_objective_ in pre-cTBS session [*log-odds* = -0.61, *z* = -5.42, *p* < 0.001, *d* = -0.34, 95% *CI* = (-0.83, -0.39)]. Nevertheless, inhibition had larger negative impact on *P*_understood_ in pre-cTBS session compared to post-cTBS session [*log-odds* = 0.66, *z* = 2.09, *p* = 0.037, *d* = 0.36, 95% *CI* = (0.04, 1.28)**; Figure 5C**].

Text 4. Detailed brain activity in single strategy results of Experiment 1

Post-hoc statistical analyses of the beta values are shown in **Figure S2** and **Table S2**.

**Auditory**

Analysis of the STC: For the significant main effect of strategy on the STC, *post-hoc* tests revealed the following differences in inhibition-related deactivation (IH vs. NP contrast):

The deactivation was significantly stronger in the distraction condition compared to the distancing condition (*t*(50) = 2.28, *p*(FDR) = 0.040, *d* = 0.40). The deactivation in the direct inhibition condition was significantly stronger than in the distancing condition (*t*(50) = 2.52, *p*(FDR) = 0.040, *d* = 0.40).

**Visual**

Analysis of the PPC: For the significant main effect of strategy on the PPC, post-hoc tests revealed that activation in the distraction condition was significantly smaller than in the distancing condition (*t*(76) = -2.95, *p*(FDR) = 0.013, *d* = -0.38). The comparisons between direct inhibition and the other two strategies showed non-significant trends (*p*-values > 0.08).

Analysis of the VC: For the significant main effect of strategy on the VC, post-hoc tests revealed the following differences in inhibition-related deactivation: The deactivation was significantly stronger in the distraction condition compared to the distancing condition (*t*(76) = 5.00, *p*(FDR) < 0.001, *d* = 0.50). The deactivation in the direct inhibition condition was significantly weaker than in the distancing condition (*t*(76) = -2.65, *p*(FDR) = 0.010, *d* = -0.28). The deactivation in the direct inhibition condition was also significantly weaker than in the distraction condition (*t*(76) = -5.85, *p*(FDR) < 0.001, *d* = -0.78).

Text 5. Detailed brain activity in conjunction results of Experiment 1

Post-hoc statistical analyses of the beta values are shown in **Table S3**, and information of each significant activation clusters are shown in **Table S4** (excluding sensory area and DMN comparisons).

**Auditory:** During the 0-6 second period, compared to NP, inhibition induced greater activation in DLPFC [post-hoc *t*(50) = 9.60, *p*(FDR) < 0.001, *d* = 1.34] and PPC [post-hoc *t*(50) = 4.97, *p*(FDR) < 0.001, *d* = 0.70], and greater deactivation in STC [post-hoc *t*(50) = -7.53, *p*(FDR) < 0.001, *d* = -1.06; **Figure 2D**].

During the 0-3 and 3-6 second periods, similar patterns (DLPFC and PPC activation, and STC deactivation) were observed. When comparing the inhibition-related activation or deactivation across the two periods, it was found that the inhibition-related increase in activity in the DLPFC and PPC were less pronounced in the post-3s period compared to the pre-3s period, though this difference did not reach significance. However, the inhibition-related decrease in activity in the STC became more pronounced in the post-3s period and was statistically significant [post-hoc *t*(50) = -6.96, *p*(FDR) < 0.001, *d* = -0.98; **Figure 2D**].

To distinguish the general deactivation of the STC from that of the DMN, we analyzed their activity across different time windows. For the entire 0-6s period, the overall magnitude of deactivation in the STC was significantly greater than that in the DMN (post-hoc *t*(50) = −6.09, *p*(FDR) < 0.001, *d* = −0.85; **Figure S4D, Table S6**). When analyzing the 0-3 s and 3-6 s time windows, a 2×2 ANOVA revealed significant main effects of both Time (*F*(1,50) = 31.77, *p* < 0.001, *η^2^* = 0.39) and ROI (*F*(1,50) = 51.98, *p* < 0.001, *η^2^* = 0.51). Critically, these effects were qualified by a significant Time × ROI interaction (*F*(1,50) = 32.97, *p* < 0.001, *η^2^* = 0.40), indicating the temporal deactivation pattern differed between regions. Post-hoc tests showed that the STC underwent a significant increase in deactivation from the early to late period (post-hoc *t*(50) = −6.96, *p*(FDR) < 0.001, *d* = −0.93), whereas the DMN’s deactivation did not significantly change over time (post-hoc *t*(50) = −0.53, *p*(FDR) = 0.601, *d* = −0.05; **Figure S4D, Table S6**).

**Visual:** During the 0-6 second period, compared to NP, inhibition induced greater activation in DLPFC [post-hoc *t*(76) = 8.49, *p*(FDR) < 0.001, *d* = 0.97] and PPC [post-hoc *t*(76) = 6.14, *p*(FDR) < 0.001, *d* = 0.70], and greater deactivation in VC [post-hoc *t*(76) = -5.38, *p*(FDR) < 0.001, *d* = -0.61; **Figure 2D**].

During the 0-3 and 3-6 second periods, similar patterns (DLPFC and PPC activation, and VC deactivation) were observed. Notably significant activation of the DLPFC and PPC was not observed, but prominent deactivation in these regions was evident. When comparing the inhibition-related activation or deactivation across the two periods, it was found that the inhibition-related increase in activity in the PPC were less pronounced in the post-3s period compared to the pre-3s period [post-hoc *t*(76) = 2.56, *p*(FDR) = 0.018, *d* = 0.29]. In contrast, the inhibition-related decrease in activity in the VC became more pronounced in the post-3s period compared to the pre-3s period [post-hoc *t*(76) = -4.25, *p*(FDR) < 0.001, *d* = -0.49; **Figure 2D**].

To distinguish the general deactivation of the VC from that of the DMN, we analyzed their activity across different time windows. For the entire 0-6s period, the overall magnitude of deactivation in the VC was significantly greater than that in the DMN (post-hoc *t*(76) = -2.45, *p*(FDR) = 0.017, *d* = -0.28; **Figure S4E, Table S6**). When analyzing the 0-3s and 3-6s time windows, we found that while there was no significant difference in the overall magnitude between the VC and the DMN (*F*(1, 76) = 0.87, *p* = 0.355, *η^2^ =* 0.01), both networks exhibited temporal deactivation (*F*(1, 76) = 25.04, *p* < 0.001, *η^2^ =* 0.25; 0-3 > 3-6: *t*(76) = , *p* < 0.001, *d* = 0.30). Critically, these effects were qualified by a significant Time × ROI interaction (*F*(1, 76) = 5.80, *p* = 0.018, *η^2^ =* 0.07), indicating the temporal deactivation pattern differed between regions. This pattern was significantly more pronounced in the VC compared to the DMN (VC: *t*(76) = -4.25, *p*(FDR) < 0.001, *d* = -0.43, DMN: *t*(76) = -3.33, *p*(FDR) = 0.001, *d* = -0.17; **Figure S4E, Table S6**).

Text 6. Detailed results of dynamic causal modeling

To investigate the effective connectivity patterns within the brain network during auditory task processing, we constructed a model space comprising 10 theory-driven models (see **Figure S4** for details). The detailed modulatory connections (B-matrix) and Bayesian model selection (BMS) for each model are presented in **SI-Table 7.**

**Auditory:** The results from the BMS analysis clearly indicated that the fully connected model (M1) provided the optimal explanation for the data. This model had the highest evidence (summed free energy *F* = -4942.4) and demonstrated a decisive superiority over the competing models. Specifically, M1 yielded an exceedance probability (EP) of 0.996, which represents a 99.6% posterior probability of this model being more prevalent than any other model in the space. We further calculated the protected exceedance probability (PEP), which was also 0.996, confirming that this finding remains robust even when accounting for the possibility of population heterogeneity. Furthermore, the Bayesian Omnibus Risk (BOR) was less than 0.001, indicating that the observed differences between the models are highly unlikely to be due to chance, providing high confidence in the outcome of our model selection.

**Visual:** The results from the BMS analysis unequivocally indicated that the fully connected model (M1) provided the optimal explanation for the data. This model had the highest evidence, as reflected by its summed free energy (*F* = -5936.45), and demonstrated decisive superiority over all competing models. Specifically, M1 yielded an EP of 1.0, representing overwhelming evidence that it was the most prevalent model in the population. We further calculated the PEP, which was also 1.0, confirming that this result is exceptionally robust, even when accounting for the possibility of population heterogeneity. Furthermore, the BOR was less than 0.001, indicating that the observed differences between the models are highly unlikely to be due to chance and providing absolute confidence in the outcome of our model selection.

Text 7. Detailed results of neural-behavioral correlations

All correlation results were shown in **Table S5 and Table S10**.

**Auditory:** Two significant correlations were observed in the overall analysis (**Table S5**). The inhibition-induced decrease in *P*_understood_ was negatively correlated with the inhibition-induced increase in DLPFC activity (*r* = -0.40, *p*(FDR) = 0.014) and positively correlated with the inhibition-induced increase in STC activity (*r* = 0.604, *p*(FDR) < 0.001). This indicates that participants who experienced a greater inhibition-induced decrease in *P*_understood_ (indicating more effective inhibition) also exhibited a greater inhibition-induced activation in DLPFC and inhibition-induced deactivation STC activity (**Figure S2 C**).

In the analysis of temporal dynamics (Table S10), we correlated behavioral changes with the post-3s vs. pre-3s difference in brain activity. The inhibition-induced decrease in *P*_understood_ was positively correlated with the post-3s vs. pre-3s difference in inhibition-induced STC deactivation (*r* = 0.45, *p*(FDR) < 0.001). This indicates that participants who experienced a greater inhibition-induced decrease in *P*_understood_ exhibited a more prominent STC low-to-high deactivation pattern (**Figure S6A**).

**Visual:** Three significant correlations were observed in the overall analysis (**Table S5**). The inhibition-induced decrease in *P*_understood_ was negatively correlated with the inhibition-induced increase in DLPFC activity (*r* = -0.289, *p*(FDR) = 0.036) but positively correlated with the inhibition-induced increase in VC activity (*r* = 0.310, *p*(FDR) = 0.036). In addition, the inhibition-induced decrease in *ACC*_objective_ was negatively correlated with the inhibition-induced increase in PPC activity (*r* = -0.284, *p*(FDR) = 0.036). This indicates that participants with a greater inhibition-induced decrease in performance also exhibited greater inhibition-induced modulation of frontoparietal and sensory cortex activity (**Figure S2 D**).

In the analysis of temporal dynamics (**Table S10**), the inhibition-induced decrease in Punderstood was positively correlated with the post-3s vs. pre-3s difference in inhibition-induced VC deactivation (*r* = 0.40, *p*(FDR) < 0.001). This indicates that participants who experienced a greater inhibition-induced decrease in Punderstood exhibited a more prominent VC low-to-high deactivation pattern (**Figure S6B**).

Text 8. Detailed results of traveling wave analysis

Post-hoc statistical analyses of the travelling wave are shown in **Figure S5B-C**. The results of occipital-frontal axis are shown in the **Figure S5B**. The results of temporal-frontal axis are shown in **Figure S5C**.

In the theta band, we observed a significant enhancement of power occurring in both forward and backward directions. For FW, theta power during inhibition was significantly greater along both the occipital-frontal axis (*t*(39) = 4.33, *p*(FDR) < 0.001, *d* = 0.69) and the temporal-frontal axis (*t*(39) = 3.06, *p*(FDR) = 0.008, *d* = 0.48). For BW, a similar significant enhancement of theta power was observed along both the occipital-frontal axis (*t*(39) = 4.10, *p*(FDR) < 0.001, *d* = 0.65) and the temporal-frontal axis (*t*(39) = 4.17, *p*(FDR) < 0.001, *d* = 0.66).

In the alpha band, we observed a direction-specific effect. Power was significantly greater during inhibition compared to NP for BW along both the occipital-frontal axis axis (*t*(39) = 7.04, *p*(FDR) < 0.001, *d* = 1.11) and the temporal-frontal axis (*t*(39) = 2.88, *p*(FDR) = 0.008, *d* = 0.46). However, no significant differences were found for FW along either the occipital-frontal axis (*t*(39) = −0.03, *p*(FDR) = 0.977, *d* = −0.01) or the temporal-frontal axis (*t*(39) = 0.22, *p*(FDR) = 0.825, *d* = 0.04).

In the beta band, we observed significant power reduction during inhibition compared to NP for both forward and backward waves across both axes. Power was significantly lower during inhibition for FW along the occipital-frontal axis (*t*(39) = −2.88, *p*(FDR) = 0.012, *d* = −0.46) and the temporal-frontal axis (*t*(39) = −3.15, *p*(FDR) = 0.008, *d* = −0.50). A similar significant reduction in power was observed for BW along the occipital-frontal axis (*t*(39) = −3.87, *p*(FDR) < 0.001, *d* = −0.61) and the temporal-frontal axis (*t*(39) = −3.01, *p*(FDR) = 0.008, *d* = −0.48).

No significant differences in traveling wave power were found in the gamma frequency band across any pathway or direction. This was consistent for FW along the occipital-frontal axis (*t*(39) = −1.94, *p*(FDR) = 0.080, *d* = −0.31) and temporal-frontal axis axes (*t*(39) = −1.71, *p*(FDR) = 0.128, *d* = −0.27), and for BW along the occipital-frontal axis (*t*(39) = −1.93, *p*(FDR) = 0.061, *d* = −0.31) and temporal-frontal axis axes (*t*(39) = −1.56, *p*(FDR) = 0.126, *d* = −0.25).

Text 9. Detailed results of EEG MVPA & Change-point detection

For the EEG MVPA, we performed two separate analyses: one using the full electrode montage and another using a subset of electrodes corresponding to the fMRI ROIs identified in the present study. The resulting decoding AUC curves for all analyses are presented in Supplementary **Figure S5E-J**. **For the 6s dataset,** the analysis with the full electrode set revealed a significant changepoint at **3.05 s**, while the ROI-based analysis identified a changepoint at **2.72 s**. **For the 10s dataset,** a significant changepoint was detected at **3.25 s** using all electrodes, and at **3.12 s** using the ROI-defined electrode subset.

Text 10. Detailed results of cross-modal decoding analysis

Descriptive statistics and statistical tests against chance level (0.5) using one-sample *t*-tests for the decoding accuracy of each condition are provided in **Table S10, 11**.

**Auditory modality as training set, visual modality as testing set:** During the 0-6 second period, the inhibition condition was modality-generalizable [*t*(26) = 4.25, *p*(FDR) < 0.001, *d* = 0.82], whereas the NP condition was not. Additionally, inhibition exhibited better modality-generalization compared to NP [*t*(26) = 3.11, *p*(FDR) = 0.008, *d* = 0.60; **Figure 3A**].

During the 0-3 second period, similar patterns were observed, the inhibition condition was modality-generalizable [*t*(26) = 4.36, *p*(FDR) < 0.001, *d* = 0.84], whereas the NP condition was not. During the 3-6 second period, the inhibition condition remained modality-generalizable [*t*(26) = 6.51, *p*(FDR) < 0.001, *d* = 1.25], whereas the NP condition continued to lack modality-generalizability. When comparing the differences in generalization accuracy between inhibition and NP across time periods, it was found that the inhibition-related increase in generalization accuracy was more pronounced in the post-3 s period compared to the pre-3 s period [*t*(26) = 2.61, *p*(FDR) = 0.030, *d* = 0.50; **Figure 3A**]. This suggest that, compare to the early 3-second period, inhibition demonstrated greater modality-generalizability than NP during the late 3-second period.

**Visual modality as training set, auditory modality as testing set:** During the 0-6 second period, the inhibition condition demonstrated cross-modal generalizability [*t*(31) = 2.12, *p* = 0.042], though this effect did not survive FDR correction [*p*(FDR) = 0.085, *d* = 0.085; **Figure 3A**], whereas the NP condition did not show cross-modal generalizability. Additionally, there was no significant difference in generalization accuracy between the two conditions.

During the 0-3 second period, the inhibition condition was modality-generalizable [*t*(31) = 3.57, *p*(FDR) = 0.005, *d* = 0.63], whereas the NP condition was not. However, during the 3-6 second period, neither the NP nor inhibition condition exhibited modality-generalizability. When comparing the differences in generalization accuracy between inhibition and NP across time periods, it was observed that the inhibition-related increase in generalization accuracy was more pronounced in the post-3 s period compared to the pre-3 s period, although this effect only reached marginal significance [*t*(31) = 1.97, *p*(FDR) = 0.057, *d* = 0.35; **Figure 3A**].

Text 11. Detailed results of feature-neural RSA

To quantify inhibition-induced decoupling, we compared neural-feature RSM correlations between inhibition and NP conditions using Steiger’s *z*-tests.

(1) Valence: correlation reduction during inhibition vs. NP (Auditory: *Δr* = -0.05, Steiger’s *z* = -0.32, *p*(FDR) = 0.983, **Figure 3B**; Visual: *Δr* = 0.26, Steiger’s *z* = -3.17, *p*(FDR) = 0.003, **Figure 3C**);

(2) Arousal: correlation reduction during inhibition vs. NP (Auditory: *Δr* = -0.44, Steiger’s *z* = -2.81, *p*(FDR) = 0.020, **Figure 3B**; Visual: *Δr* = -0.36, Steiger’s *z* = -0.61, *p*(FDR) = 0.072, **Figure 3C**);

(3) Information load: correlation reduction during inhibition vs. NP (Auditory: *Δr* = 0.03, Steiger’s *z* = 0.19, *p*(FDR) = 0.983, **Figure 3B**; Visual: *Δr* = -0.07, Steiger’s *z* = -3.17, *p*(FDR) = 0.003, **Figure 3C**);

(4) Processing difficulty: correlation reduction during inhibition vs. NP (Auditory: *Δr* = -0.004, Steiger’s *z* = -0.02, *p*(FDR) = 0.983, **Figure 3B**; Visual: *Δr* = -0.22, Steiger’s *z* = -3.17, *p*(FDR) = 0.003, **Figure 3C**);

Text 12. Detailed results of unimodal MVPA analysis

Descriptive statistics and statistical tests against chance level (0.5) using one-sample *t*-tests for the decoding accuracy of each condition are provided in **Tables S12-13**.

**Auditory:** During the 0-6 second period, both NP and inhibition conditions were decodable in each of the three ROIs [all *t*(31) > 2.55, *p*(FDR) < 0.019, *d* > 0.64], with the exception of NP in the DLPFC, which was not decodable. The decoding had acceptable effect sizes (AUC range: 0.65-0.66). Notably, inhibition was better decoded than NP in the DLPFC [*t*(31) = 2.30, *p*(FDR) = 0.042, *d* = 0.41] while NP was better decoded than inhibition in the STC [*t*(31) = -2.66, *p*(FDR) = 0.036, *d* = -0.47; **Figure 4B**]. This suggests that compared to NP, inhibition increased decoding accuracy in the DLPFC and PPC but decreased it in the STC.

During the 0-3 second period, similar patterns were observed, with both NP and inhibition conditions being decodable in all three ROIs [all *t*(31) > 3.31, *p*(FDR) < 0.005, *d* > 0.83; **Figure 4D**], except for NP in the DLPFC. The decoding had acceptable effect sizes (AUC range: 0.60-0.68). The same trend persisted in the 3-6 second period [all *t*(31) > 2.19, *p*(FDR) < 0.048, *d* > 0.55; **Figure 4D**], except for NP in the DLPFC and inhibition in the STC. The decoding in the 3-6 second had acceptable effect sizes (AUC range: 0.60-0.63).When comparing the decoding accuracy differences between inhibition and NP across time periods, it was found that the inhibition-related increase in decoding accuracy in the DLPFC and PPC was less pronounced in the post-3 s period compared to the pre-3 s period, though this difference did not reach significance. However, the inhibition-related decrease in decoding accuracy in the STC became more pronounced in the post-3 s period compared to the pre-3 s period, and was statistically significant [*t*(31) = -3.32, *p*(FDR) = 0.006, *d* = -0.59; **Figure 4D**].

**Visual:** During the 0-6 second period, both NP and inhibition conditions were decodable in each of the three ROIs, with the exception of NP in the DLPFC [all *t*(26) > 5.74, *p*(FDR) < 0.001, *d* > 1.07] with moderate effect sizes (AUC range: 0.67-0.70). Notably, inhibition was better decoded than NP in the DLPFC [*t*(26) = 2.31, *p*(FDR) = 0.029, *d* = 0.44] and PPC[*t*(26) = 2.34, *p*(FDR) = 0.029, *d* = 0.45] while NH was better decoded than inhibition in the VC [*t*(26) = -2.53, *p*(FDR) = 0.029, *d* = -0.49; **Figure 4C**]. This suggests that compared to NP, inhibition increased decoding accuracy in the DLPFC and PPC but decreased it in the VC.

During the 0-3 second period, similar patterns were observed, with both NP and inhibition conditions being decodable in all three ROIs [all *t*(26) > 3.76, *p*(FDR) < 0.001, *d* > 0.72; **Figure 4D**] with great effect sizes (AUC range: 0.77-0.85). The same trend persisted in the 3-6 second period [all *t*(26) > 4.25, *p*(FDR) < 0.001, *d* > 0.81; **Figure 4D**] with great effect sizes (AUC range: 0.81-0.87). When comparing the decoding accuracy differences between inhibition and NP across time periods, it was found that the inhibition-related increase in decoding accuracy in the all three ROIs was less pronounced in the post-3 s period compared to the pre-3 s period [DLPFC: *t*(26) = -2.37, *p*(FDR) = 0.043, *d* = -0.46; PPC: *t*(26) = -2.13, *p*(FDR) = 0.043, *d* = -0.41; **Figure 4D**]. In contrast, the inhibition-related decrease in decoding accuracy in the VC became more pronounced in the post-3 s period compared to the pre-3 s period [*t*(26) = -2.19, *p*(FDR) = 0.043, *d* = -0.42; **Figure 4D**].

To ensure that data smoothing did not impact the MVPA results in this study, we performed a validation analysis using the 0-6s visual data. For this, the data were re-preprocessed using an identical pipeline to the smoothed data but omitting the smoothing step (see “**Experimental section**”, “**Preprocessing and subject-level analyses**”), after which the analysis was run directly. The results revealed that with the unsmoothed data, both the NP and inhibition conditions were decodable in all three ROIs [all *t*(26) > 4.07, *p*(FDR) < 0.001, *d* > 0.81]. Furthermore, these results did not differ significantly from those obtained using the smoothed data [all *t*(26) < 1.67, *p*(FDR) > 0.385, *d* < 0.32].

Text 13. Detailed results of feature MVPA

Descriptive statistics and statistical tests against chance level (0.25) using one-sample *t*-tests for the decoding accuracy of each condition are provided in **Table S14**.

**Valence:** The accuracy of all conditions was greater than the chance level of 0.25, which indicates that all conditions are decodable [all *t*(26) > 4.70, *p*(FDR) < 0.001, *d* > 0.90] with acceptable effect sizes (AUC range: 0.62-0.64). There was a significant interaction (task condition*feature intensity) on decoding accuracy [*F*(1, 26) = 6.06, *p* = 0.021, *η^2^* = 0.003]. A simple effects analysis indicated that, high and low valence can be distinguished in NP [*F*(1, 26) = 6.06, *p* = 0.021, *η²* = 0.28], but not in inhibition [*F*(1,26) = 0.08, *p* = 0.786, *η²* = 0.003; **Figure 4E**].

**Arousal:** The accuracy of all conditions was greater than the chance level of 0.25, which indicates that all conditions are decodable [all *t*(26) > 5.85, *p*(FDR) < 0.001, *d* > 1.13] with acceptable effect sizes (AUC range: 0.60-0.66). The interaction (task condition*feature intensity) on decoding accuracy was not significant [*F*(1, 26) = 3.07, *p* = 0.091, *η²* = 0.11; **Figure 4E**].

**Information load:** The accuracy of all conditions was greater than the chance level of 0.25, which indicates that all conditions are decodable [all *t*(26) > 4.35, *p*(FDR) < 0.001, *d* > 0.84] with acceptable effect sizes (AUC range: 0.60-0.72). There was a significant interaction (task condition*feature intensity) on decoding accuracy [*F*(1, 26) = 9.73, *p* = 0.004, *η²* = 0.27]. A simple effects analysis indicated that, high and low arousal can be distinguished in NP [*F*(1, 26) = 7.74, *p* = 0.010, *η²* = 0.23], but not in inhibition [*F*(1, 26) = 3.50, *p* = 0.073, *η²* = 0.12; **Figure 4E**].

**Processing difficulty:** The accuracy of all conditions was greater than the chance level of 0.25, which indicates that all conditions are decodable [all *t*(26) > 3.49, *p*(FDR) < 0.002, *d* > 0.67] with acceptable effect sizes (AUC range: 0.57-0.63). There was a significant interaction (task condition*feature intensity) on decoding accuracy [*F*(1, 26) = 9.55, *p* = 0.005, *η²* = 0.27]. A simple effects analysis indicated that, high and low arousal can be distinguished in NP [*F*(1,26) = 27.26, *p* < 0.001, *η²* = 0.51], but not in inhibition [*F*(1, 26) = 0.94, *p* = 0.341, *η²* = 0.06; **Figure 4E**].

Text 14. Detailed brain activity results of Experiment 4

Post-hoc statistical analyses of the beta values were shown in **Table S15**. Information of each significant activation clusters were shown in **Table S16**.

During the 0-6 second periods, the inhibition > NP, NP > inhibition contrasts in both the pre-cTBS and post-cTBS conditions observed similar brain activity patterns (higher DLPFC and PPC activation, and STC deactivation; **Figure 5D**). When comparing the two TMS conditions, it was found that cTBS induced lower DLPFC and PPC activation [DLPFC: *t*(35) = 3.77, *p*(FDR) < 0.001, *d* = 0.63; PPC: *t*(35) = 3.90, *p*(FDR) < 0.001, *d* = 0.65], and STC deactivation [*t*(35) = -2.40, *p*(FDR) = 0.022, *d* = -0.40; **Figure 5D**]*.*

During the 0-3 and 3-6 second periods, the flexible factorial analysis demonstrated significant interaction effects between TMS *status* × *time* in the DLPFC and STC (**Figure 5E**). According to the *post-hoc* ANOVA of mean parameter estimates for each ROI, these interactions were explained by the fact that, the temporal deactivation pattern in DLPFC and STC (as calculated by post3 s – pre3 s difference) in post-cTBS condition was less prominent compared to the pre-cTBS condition [DLPFC: *t*(35) = --2.27, *p*(FDR) = 0.044, *d* = -0.36; STC: *t*(35) = -2.69, *p*(FDR) = 0.033, *d* = -0.45]*.*

Text 15. Detailed results of mediation analysis

We conducted a mediation analysis to test whether temporal deactivation mediated the effect of TMS stimulation on inhibitory performance (**Figure 5F**).

The **total effect** of TMS on inhibitory performance was not significant (*c* = -0.04, SE = 0.03, *t* = -1.29, *p* = 0.203, 95% CI [-0.10, 0.02]). The **direct effect** of TMS stimulation on inhibitory performance, controlling for temporal deactivation, was also not significant (*c’* = -0.02, SE = 0.03, *t* = -0.69, *p* = 0.492, 95% CI [-0.08, 0.04]).

TMS stimulation significantly predicted reduced temporal deactivation (**a-path**: *a* = -0.40, SE = 0.16, *t* = -2.43, *p* = 0.018, 95% CI [-0.73, -0.07]).

Temporal deactivation significantly predicted inhibitory performance (**b-path**: *b* = 0.04, SE = 0.02, *t* = 2.05, *p* = 0.045, 95% CI [0.001, 0.08]).

Critically, a significant indirect effect was observed from TMS stimulation to inhibitory performance via temporal deactivation (*a × b* = -0.02, Boot SE = 0.01, Boot 95% CI [-0.04, -0.0009]), indicating full mediation.

The finding supports our hypothesis that the disruptive effects of TMS on inhibitory performance were mediated entirely through the reduction of temporal deactivation.

Text 16. Detailed brain activity results of Experiment 3--”Concentration” model

Post-hoc statistical analyses of the beta values were shown in **Table S17**. Information of each significant activation clusters were shown in **Table S18**.

During the 0-6 second periods, inhibition vs. NP contrast observed similar brain activity patterns (higher DLPFC and PPC activation, and STC deactivation). The concentration vs. NP contrast only observed higher DLPFC and PPC activation (Figure S3B). When comparing the two contrasts, it was found that inhibition vs. NP induced higher DLPFC and PPC activation [post-hoc DLPFC: *t*(14) = -3.96, *p*(FDR) = 0.003, *d* = -1.02; PPC: *t*(14) = -3.26, *p*(FDR) = 0.008, *d* = -0.84], and STC deactivation [post-hoc *t*(31) = 3.11, *p*(FDR) = 0.008, *d* = 0.80; **Figure S7B**]*.*

During the 0-3 and 3-6 second periods, the flexible factorial analysis demonstrated significant interaction effects between task condition × time in the DLPFC and STC (Figure 6A). According to the *post-hoc* ANOVA of mean parameter estimates for each ROI, these interactions were explained by the fact that, the post3 s – pre3 s difference in DLPFC and STC activation in inhibition was prominent compared to the concentration condition [post-hoc DLPFC: *t*(14) = 2.38, *p*(FDR) = 0.048, *d* = 0.62; STC: *t*(14) = 4.60, *p*(FDR) = 0.003, *d* = 1.19; **Figure 6A**]*.*

Text 17. Detailed brain activity results of Experiment 3--”Scramble” model

Post-hoc statistical analyses of the beta values were shown in **Table S19**. Information of each significant activation clusters were shown in **Table S20**.

During the 0-6 second periods, both inhibition vs. NP normal, and inhibition vs. NP scramble contrasts observed similar brain activity patterns (higher DLPFC and PPC activation, and STC deactivation; Figure S3B). When comparing the two contrasts, it was found that inhibition vs. NP normal induced higher DLPFC activation [post-hoc *t*(14) = -2.85, *p*(FDR) = 0.039, *d* = -0.74; **Figure S7B**].

During the 0-3 and 3-6 second periods, the flexible factorial analysis did not reveal significant interaction effects between task condition × time × voice type in all ROIs*.*

Text 18. Detailed results of neural-neural RSA

**“Concentration” model for auditory experiment:** During the 0-6 second period, the similarity between the RSMs for concentration vs. NP and inhibition vs. NP was relatively low (*r* = 0.06, *p*(FDR) = 0.496; Figure 6B). During the 0-3 second period, the similarity between the RSMs for concentration vs. NP and inhibition vs. NP remained low (*r* = 0.03, *p*(FDR) = 0.627; Figure 6B). During the 3-6 second period, the similarity between the RSMs for concentration vs. NP and inhibition vs. NP was low (*r* = 0.09, *p*(FDR) = 0.350; **Figure 6B**).

**“Scramble” model for auditory experiment:** During the 0-6 second period, the similarity between the RSMs for inhibition vs. NP normal and inhibition vs. NP scramble was relatively high (*r* = 0.12, *p*(FDR) < 0.044; Figure 6B). During the 0-3 second period, the similarity between the RSMs for inhibition vs. NP normal and inhibition vs. NP scramble remained high (*r* = 0.21, *p*(FDR) < 0.001; Figure 6B). During the 3-6 second period, the similarity between the RSMs for inhibition vs. NP normal and inhibition vs. NP scramble decreased, though it was still significant (*r* = 0.35, *p*(FDR) < 0.001; **Figure 6B**).

**“Close-eye” model for visual experiment:** During the 0-6 second period, the similarity between the RSMs for inhibition vs. NP and close-eye vs. NP scramble was relatively high (*r* = 0.92, *p*(FDR) < 0.001; Figure S3C). During the 0-3 second period, the similarity between the RSMs for inhibition vs. NP normal and inhibition vs. NP scramble remained high (*r* = 0.87, *p*(FDR) < 0.001; **Figure S7C**). During the 3-6 second period, the similarity between the RSMs for inhibition vs. NP normal and inhibition vs. NP scramble decreased, though it was still significant (*r* = 0.90, *p*(FDR) < 0.001; **Figure S7C**).


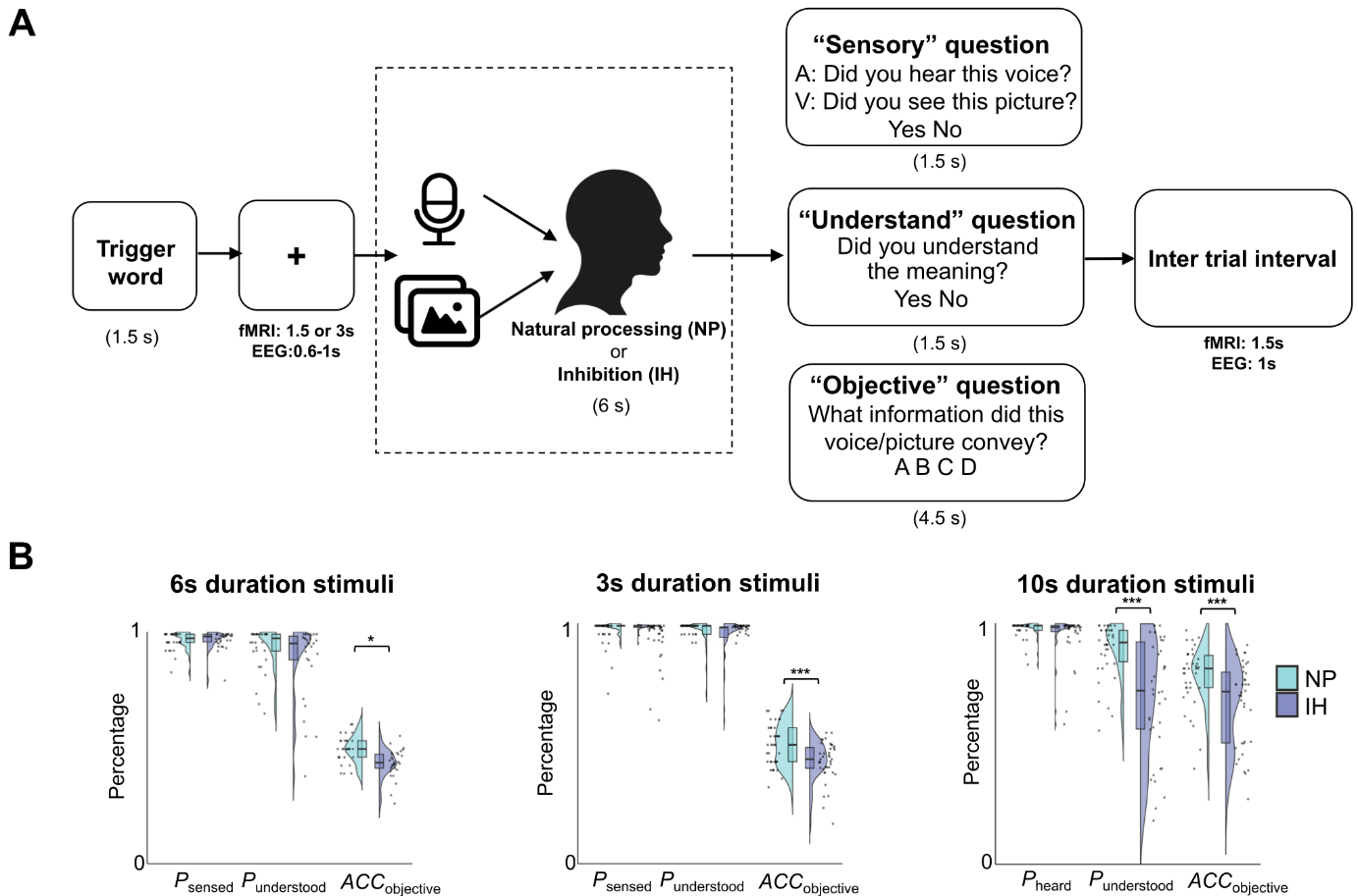


**Figure S1. Schematic of the experimental paradigm and behavioral results of Experiment 2. A.** The procedure of a single trial in the auditory and visual inhibition experiments. Each trial began with the presentation of a trigger word (e.g., “Natural processing,” “Direct inhibition”) for 1.5 s, informing the participant of the cognitive strategy to be adopted. This was followed by a fixation cross (+), with a duration of 1.5 or 3 s in the fMRI experiment and 0.6-1 s in the EEG experiment. Subsequently, a 6-s stimulus (voice clip or picture) was presented, during which participants performed either the “natural processing” (NP) or “inhibition” (IH) task according to the preceding instruction. After the stimulus presentation, participants were required to answer three consecutive questions: a “Sensory question” (“Did you hear/see this stimulus?”; 1.5 s), an “Understand question” (“Did you understand its meaning?”; 1.5 s), and a four-alternative forced-choice “Objective question” (4.5 s). Each trial concluded with an Inter trial interval, which lasted 1.5 s in the fMRI experiment and 1 s in the EEG experiment. **B.** Behavioral performance of Experiment 2 across 6-second (left panel), 3-second (middle panel), and 10-second (right panel) of voice stimuli. Raincloud plots represent the distribution of responses for *P*_sensed_, *P*_understood_ and *ACC*_objective_, comparing NP with inhibition. The box plots embedded in the raincloud plots represent the median (center line), interquartile range (boxes; 25–75th percentiles), and whiskers extending to the most extreme data points within 1.5 times the interquartile range. Data beyond this range are considered outliers (same definition applies below). Data were onbtained from Experiment 2, involving independent participant samples (6s: n = 40; 3s: n = 30, 10s: n = 38). Each data point represents an individual participant (biological replicate). Statistical comparisons (inhibition vs. NP condition) were performed using paired *t*-tests on the mean values of each condition. **p*(FDR) < 0.05, ***p*(FDR) < 0.01, ****p*(FDR) < 0.001, (two-sided).


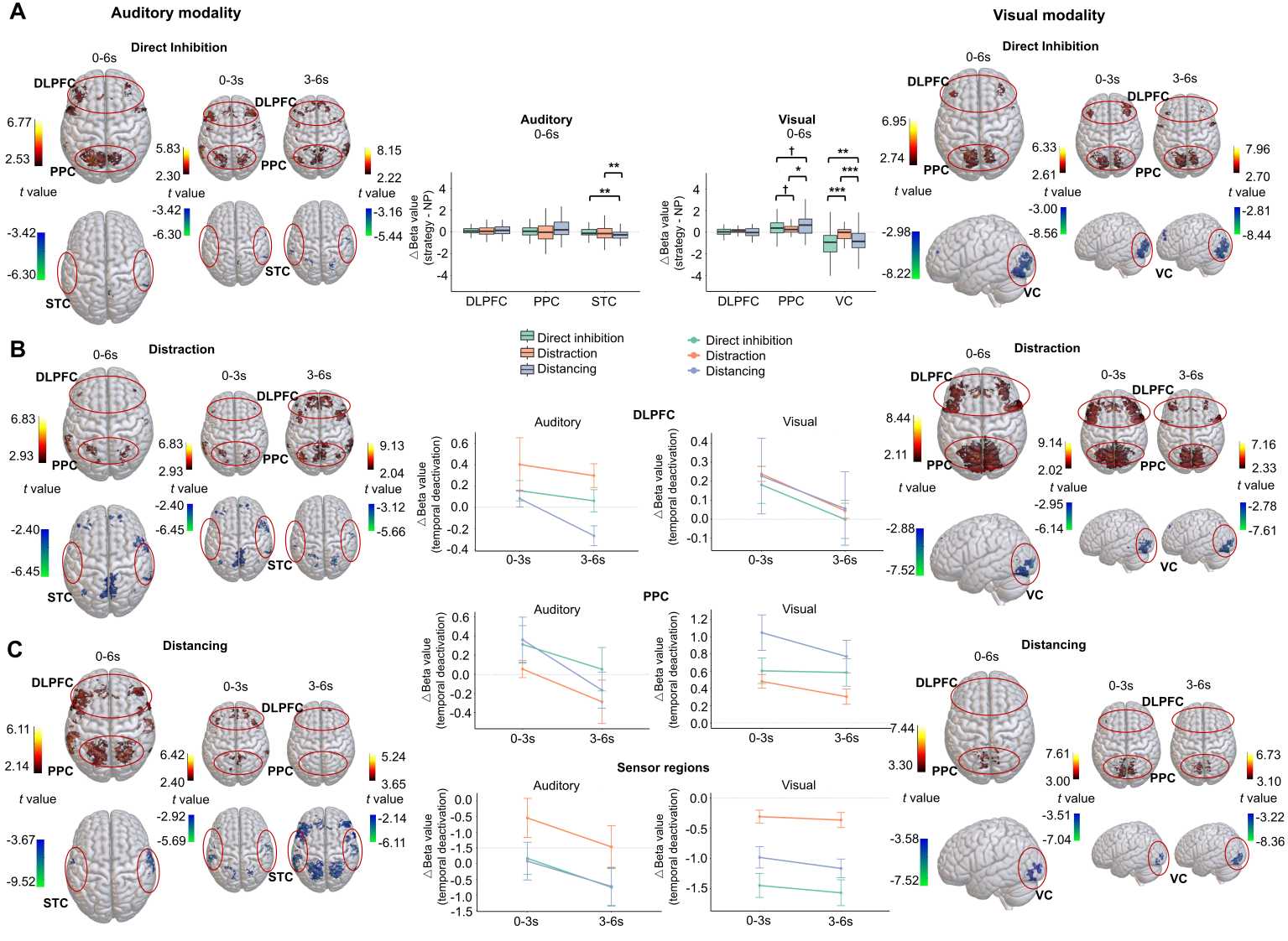


**Figure S2. Convergent deactivation dynamics across three inhibitory strategies in auditory and visual modalities.** **A. *Direct inhibition.*** Surface‐rendered MRI maps show voxels where the contrast IH > NP yields significant activation (warm colors) or deactivation (cool colors) within the DLPFC, PPC, and modality-specific sensory cortex—STC for the auditory task (left) and VC for the visual task (right). Whole-trial (0–6 s) maps are accompanied by early (0–3 s) and late (3–6 s) windows. Central Line charts depict IH–NP *β*-value differences (*Δβ*) in each ROI **B. *Distraction.*** Layout as in A, but for the distraction strategy. Line charts (center) display mean ± the standard error of the mean (SEM) *Δβ* values across the two temporal windows. **C. *Distancing.*** Same layout as B, but for the distancing strategy. Across all strategies and sensory modalities, inhibition consistently produced (i) decreasing frontoparietal activation and (ii) increasing sensory-cortex deactivation over time, supporting a modality-invariant feed-forward signal-attenuation mechanism for sustained inhibition. Data in this figure are from Experiment 1, involving independent participant samples (Auditory: n = 51; Visual: n = 77). Each data point represents an individual participant (biological replicate). Statistical comparisons (inhibition vs. NP conditions, pre-3s vs. post-3s periods) were performed using paired t-tests on the mean values of each condition. **p*(FDR) < 0.05, ***p*(FDR) < 0.01, ****p*(FDR) < 0.001). Line charts (center) display mean ± SEM. All quantitative analyses were conducted using the full anatomical ROI masks, with colored voxels within each mask denoting the regions of maximal statistical significance.


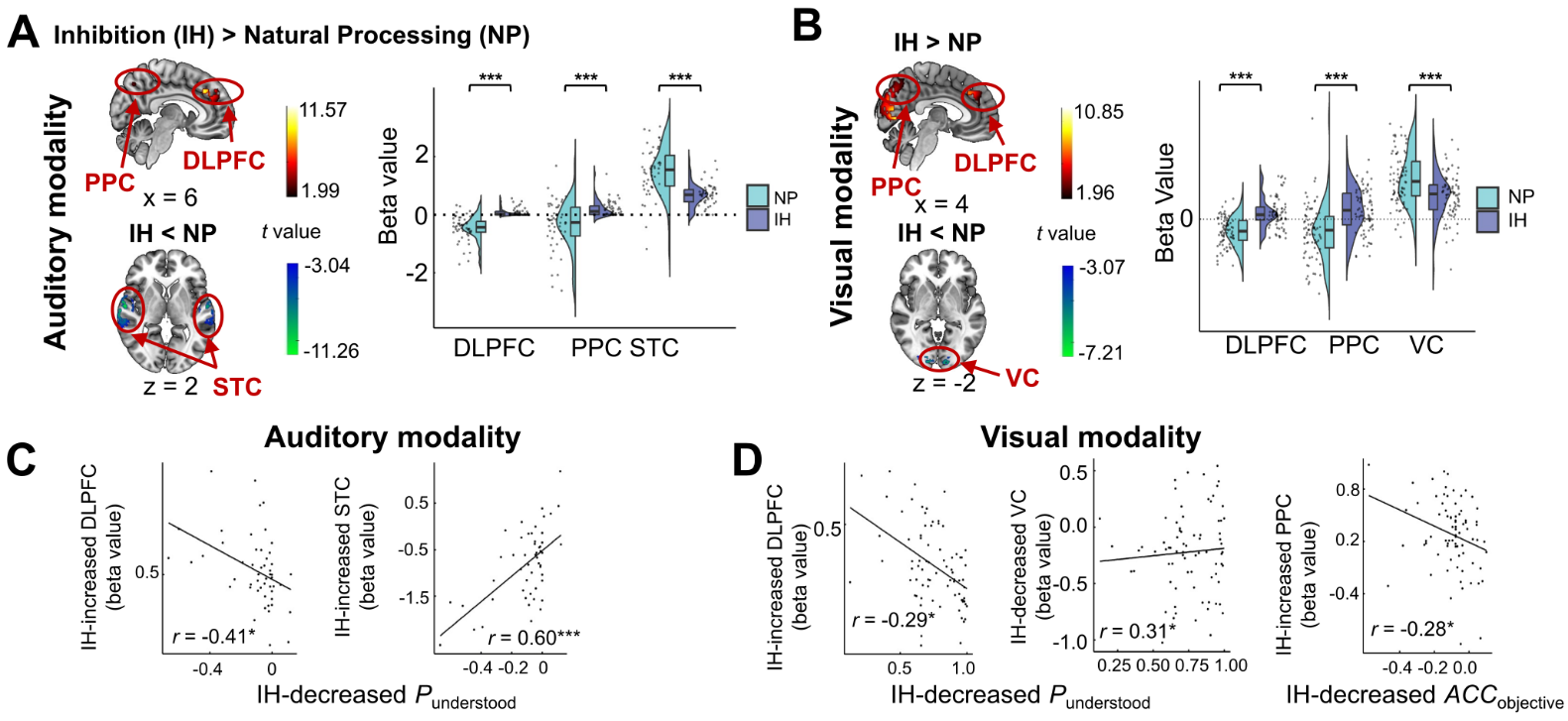


**Figure S3. Additional results of brain responses during inhibition. A.** fMRI results of the Auditory experiment. Brain activation and deactivation maps show the differences between “natural processing” (NP) and “inhibition” (IH) conditions in the predefined ROIs—the DLPFC, PPC, and STC. Raincloud plots represent the distribution of beta values. **B.** fMRI results of the Visual experiment. Brain activation and deactivation maps show the differences between IH and NP conditions in the predefined ROIs—the DLPFC, PPC, and VC. Raincloud plots represent the distribution of beta values. The box plots embedded in the raincloud plots represent the median (center line), interquartile range (boxes; 25th–75th percentiles), and whiskers extending to the most extreme data points within 1.5 × the interquartile range; data beyond this range are considered outliers. All the voxels presented are statistically significant. **C & D.** Correlations between inhibition-related changes in behavioral performance and fMRI activation (beta values) in the Auditory experiment **C** and Visual experiment **D**. Data in this figure are from Experiment 1, involving independent participant samples (Auditory: n = 51; Visual: n = 77). Each data point represents an individual participant (biological replicate). Statistical comparisons between IH and NP conditions were performed using paired t-tests. Correlation significance was assessed using Pearson correlation coefficients. **p*(FDR) < 0.05, ***p*(FDR) < 0.01, ****p*(FDR) < 0.001 (two-sided). All quantitative analyses were conducted using the full anatomical ROI masks, with colored voxels within each mask denoting the regions of maximal statistical significance.


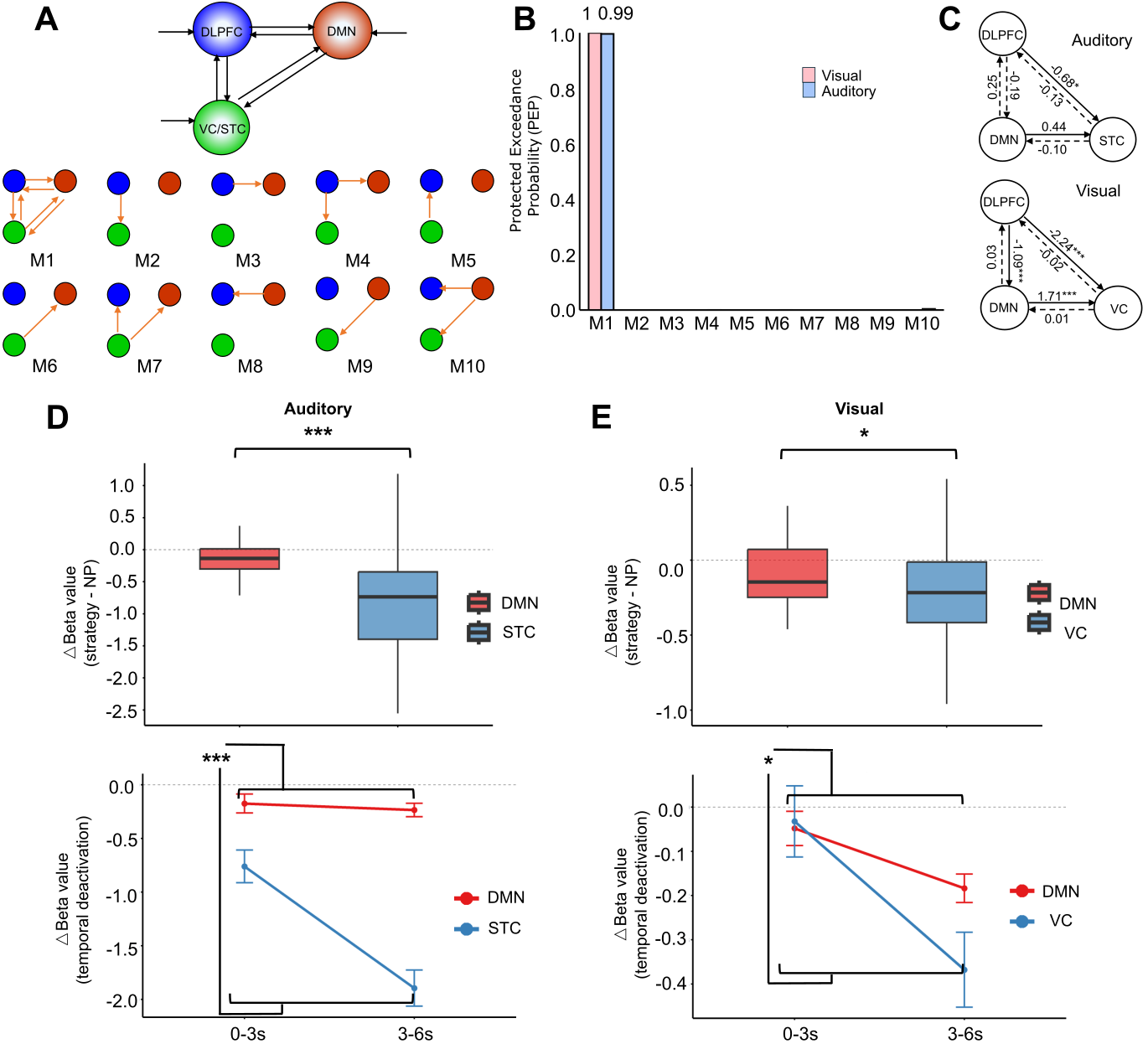


**Figure S4. Functional dissociation between sensory cortex and DMN, and effective connectivity during inhibition.** **A.** Schematic of the ten competing DCM models tested to investigate the effective connectivity between the DLPFC, the DMN, and the sensory cortex (VC/STC). **B.** Model comparison results. Bar plots show the EP for each of the four models in the visual (blue) and auditory (pink) experiments, indicating that the fully-connected model (M1) was the winning model in both modalities. **C.** Parameter estimates of the task-modulated connections (inhibition condition) from the winning DCM model for the visual (top) and auditory (bottom) experiments. **D.** Comparison of inhibition-related activity between the VC and the DMN of Visual in Experiment 1. **E.** Comparison of inhibition-related activity between the STC and the DMN of Visual in Experiment 1. Top panels: Box plots showing the overall deactivation (Δ Beta value for Strategy - NP). Bottom panels: Line plots showing the magnitude of temporal deactivation (the change in beta values from the early 0-3s phase to the late 3-6s phase). Error bars represent one SEM. Line charts (center) display mean ± SEM. The values represent the strength of the modulatory influence in Hz. Solid lines represent significant pathways. **p*(FDR) < 0.05, ****p*(FDR) < 0.001.


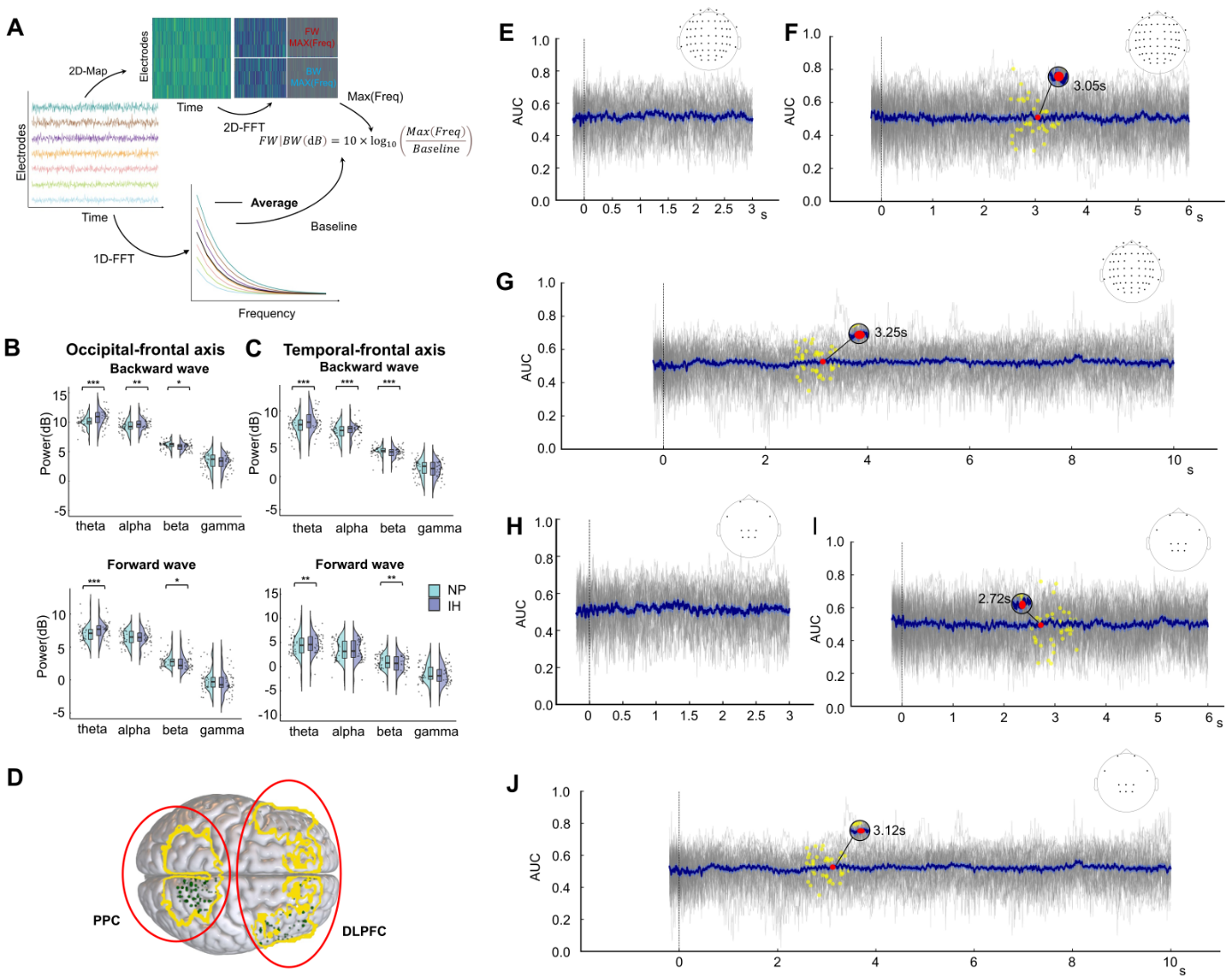


**Figure S5. The EEG traveling wave analysis, source localization, and decoding-based change point analysis.** **A.** Schematic of the traveling wave analysis pipeline. For a set of electrodes, the time-series data is converted into a 2D map (electrodes × time). A 2D-FFT is applied to this map. The power of the FW and BW is extracted and normalized in dB relative to a baseline, which is calculated from the average 1D-FFT across the selected electrodes. **B.** Traveling wave power along the Occipital-frontal axis. Raincloud plots show the power (dB) for backward waves (top) and forward waves (bottom) across the theta, alpha, beta, and gamma frequency bands for both the “natural processing” (NP) and “inhibition” (IH) conditions. **C.** Traveling wave power along the Temporal-frontal axis. Raincloud plots show the power (dB) for backward waves (top) and forward waves (bottom) across the four frequency bands for both the NP and IH conditions. **D.** The EEG source localization results show that the yellow Contours represent the predefined ROI boundaries, and the green dots are the clusters where significant differences in IH and NP were found in the traceability analysis. In panels **E-J**, the blue curve indicates the average decoding AUC, with the blue shaded area representing the ± SEM confidence interval. The red circle marks the change point for the average curve. Gray curves show individual participant decoding AUCs, and yellow circles mark their respective change points. **E-G.** Temporal decoding accuracy curves from a MVPA using the full electrode montage for 3s (E), 6s (F, min individual change point: 2.54s, max individual change point: 3.44s, average curve change point: 3.05s), and 10s (G, min individual change point: 2.72s, max individual change point: 3.22s, average curve change point: 3.25s) duration stimuli. **H-J.** Temporal decoding accuracy curves from an fMRI-guided MVPA, using a subset of electrodes corresponding to the fMRI defined ROIs. The analysis was performed on 3s (H), 6s (I, min individual change point: 2.54s, max individual change point: 3.42s, average curve change point: 2.72s), and 10s (J, min individual change point: 2.60s, max individual change point: 3.45s, average curve change point: 3.12s) duration stimuli. Data are from Experiment 2, involving independent participant samples (6s stimuli: n = 40; 3s stimuli: n = 30; 10s stimuli: n = 38). Each data point represents an individual participant. Statistical comparisons between IH and NP conditions were performed using paired t-tests. **p*(FDR) < 0.05, ***p*(FDR) < 0.01, ****p*(FDR) < 0.001.


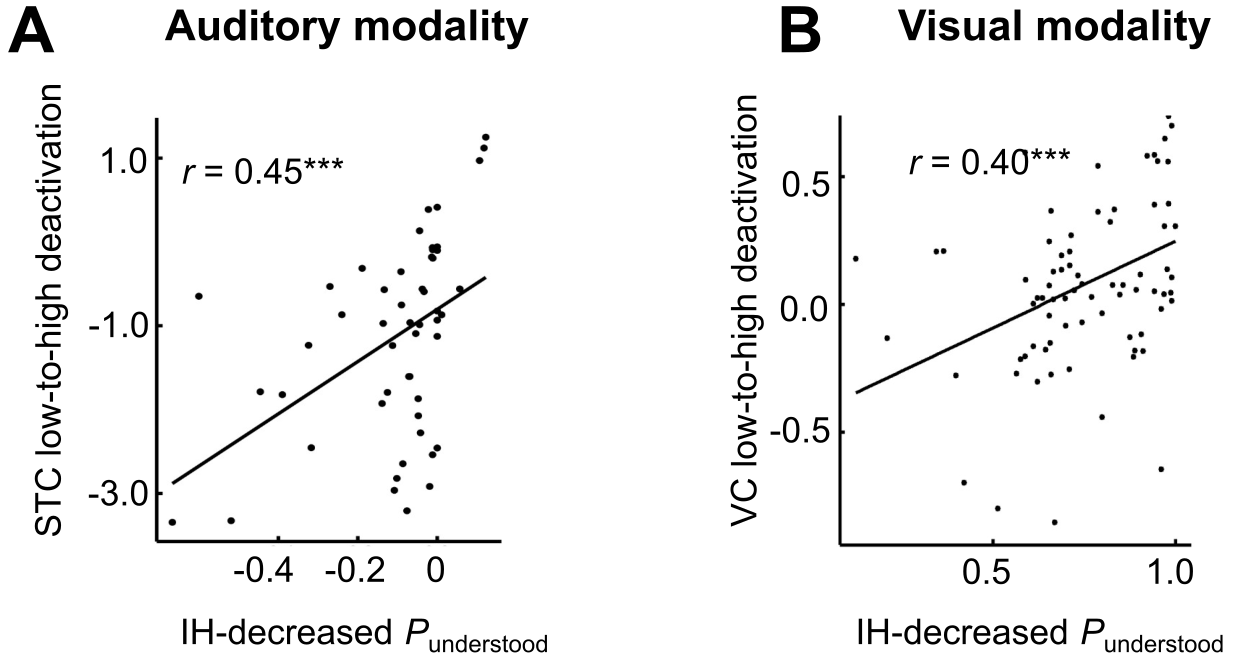


**Figure S6.** Correlations between inhibition-related changes in behavioral performance and fMRI temporal deactivation (pre-3 s vs. post-3 s periods) in the Auditory experiment **A** and Visual experiment **B**. Data are from Experiment 1, involving independent participant samples. Each data point in the scatter plots represents an individual participant. Correlation significance was assessed using Pearson correlation coefficients. Asterisks denote significance levels: **p*(FDR) < 0.05, ****p*(FDR) < 0.001.


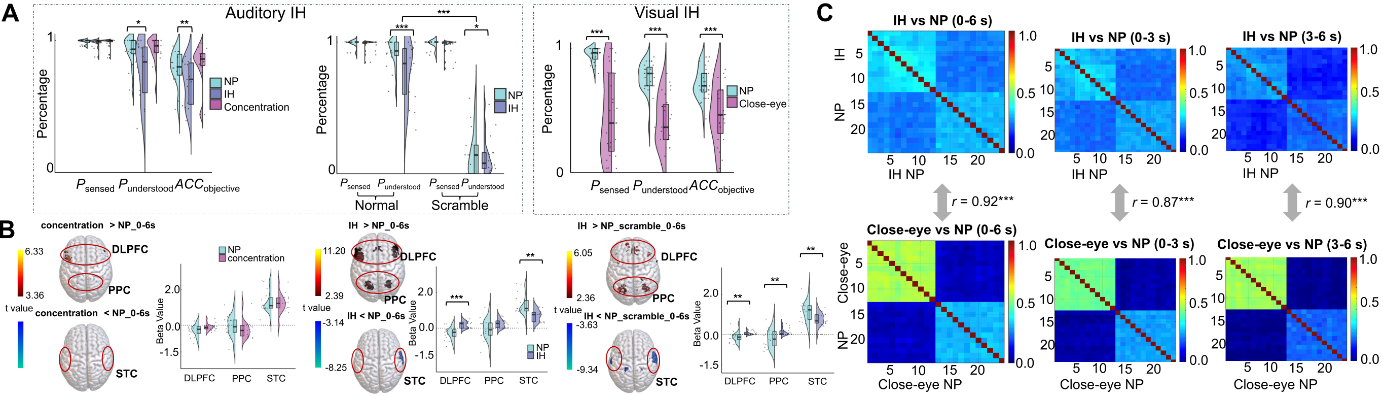


**Figure S7. Additional results of the link between inhibition and brain deactivation patterns. A.** Behavioral results of the “Concentration” model for Auditory (left panel), the “Scramble” model for Auditory (middle panel), and “Close-eye” model for Visual (right panel). **B.** Brain activation and deactivation differences for the following comparisons during the whole 0-6s periods: concentration vs. NP (left panel), inhibition vs. NP (middle panel), inhibition vs. NP-scramble (right panel). Brain maps display activation and deactivation, and raincloud plots depict beta values, segmented into early and late periods. All the voxels presented are statistically significant. **C.** Correlations between neural RSMs for the following contrasts across different time windows (0-6 s, 0-3 s, 3-6 s) in Visual experiment: inhibition vs. NP and close-eye vs. NP. Data are from Experiment 3, involving independent participant samples (Auditory: n = 15; Visual: n = 27). Each data point represents an individual participant (biological replicate). Statistical comparisons (inhibition vs. NP, concentration vs. NP, close-eye vs. NP, pre-3 s vs. post-3 s periods) were performed using paired *t*-tests. The significance of correlations between neural RSMs was determined using Pearson correlation coefficients. **p*(FDR) < 0.05, ***p*(FDR) < 0.01, ****p*(FDR) < 0.001 (two-sided). All quantitative analyses were conducted using the full anatomical ROI masks, with colored voxels within each mask denoting the regions of maximal statistical significance.

**Table S1. Descriptive statistics (M ± SD) of each type of responses under different conditions across all participants in Experiments 1, 2 (a), Experiment 3 (b), and Experiment 4 (c).** All rates were taken as the average positive response under each condition. Note: Response rate data of NP and inhibition under normal semantic order condition in Experiment 3 was extracted from the same group of participants’ first visit in Experiment 1.

**a.**

| Positive response rate | Task condition | Experiment 1 | | Experiment 2 | | |
| --- | --- | --- | --- | --- | --- | --- |
|  |  | Auditory (n = 51) | Visual (n = 77) | 2.1 (n=30) | 2.2 (n=40) | 2.3 (n=38) |
| *P*_sensed_ | NP | 0.98 ± 0.06 | 0.96 ± 0.1 | 0.97 ± 0.04 | 0.99 ± 0.02 | 0.99 ± 0.03 |
|  | IH | 0.97 ± 0.05 | 0.93 ± 0.14 | 0.97 ± 0.04 | 0.99 ± 0.02 | 0.96 ± 0.09 |
| *P*_understood_ | NP | 0.92 ± 0.12 | 0.89 ± 0.15 | 0.94 ± 0.10 | 0.98 ± 0.06 | 0.90 ± 0.11 |
|  | IH | 0.81 ± 0.21 | 0.76 ± 0.19 | 0.89 ± 0.17 | 0.95 ± 0.09 | 0.70 ± 0.24 |
| *ACC*_objective_ | NP | 0.92 ± 0.16 | 0.82 ± 0.15 | 0.50 ± 0.06 | 0.50 ± 0.09 | 0.79 ± 0.13 |
|  | IH | 0.82 ± 0.16 | 0.72 ± 0.16 | 0.44 ± 0.06 | 0.43 ± 0.08 | 0.67 ± 0.18 |

NP, natural processing, IH, inhibition

**b.**

| Positive Response Rate | Task Condition | Experiment 3 | | |
| --- | --- | --- | --- | --- |
|  |  | Auditory-normal order (n=15, revisited participants from Experiment 1-Auditory) | Auditory-scrambled order (n=15, revisited participants from Experiment 1-Auditory) | Visual (n=27, revisited participants from Experiment 1-Visual) |
| *P*_sensed_ | NP | 0.99 ± 0.03 | 0.97 ± 0.03 | 0.95 ± 0.06 |
|  | IH | 0.98 ± 0.04 | 0.96 ± 0.07 | 0.93 ± 0.09 |
|  | Concentration | 0.99 ± 0.05 |  |  |
|  | Close-eye |  |  | 0.47 ± 0.35 |
| *P*_understood_ | NP | 0.94 ± 0.11 | 0.15 ± 0.20 | 0.77 ± 0.12 |
|  | IH | 0.79 ± 0.20 | 0.12 ± 0.16 | 0.66 ± 0.14 |
|  | Concentration | 0.91 ± 0.13 |  |  |
|  | Close-eye |  |  | 0.41 ± 0.22 |
| *ACC*_objective_ | NP | 0.75 ± 0.22 |  | 0.72 ± 0.13 |
|  | IH | 0.66 ± 0.17 |  | 0.67 ± 0.14 |
|  | Concentration | 0.82 ± 0.19 |  |  |
|  | Close-eye |  |  | 0.47 ± 0.25 |

NP, natural processing, IH, inhibition

**c.**

| Positive response rate | Task condition | Experiment 4 | |  |
| --- | --- | --- | --- | --- |
|  |  | Task 1 (*N* = 36) | Task 2 (*N* = 36) |  |
|  |  |  |  |  |
| *P*_sensed_ | NP | 1.00 ± 0.01 | 0.99 ± 0.02 |  |
|  | IH | 0.99 ± 0.02 | 0.99 ± 0.01 |  |
| *P*_understood_ | NP | 0.97 ± 0.05 | 0.97 ± 0.06 |  |
|  | IH | 0.87 ± 0.14 | 0.90 ± 0.15 |  |
| *ACC*_objective_ | NP | 0.97 ± 0.11 | 0.97 ± 0.11 |  |
|  | IH | 0.87 ± 0.14 | 0.90 ± 0.13 |  |

**Table S2. Post-hoc beta values (*M ± SD*) of each individual strategy for comparisons in Experiment 1: Analysis of stimulus exposure periods (a) 0-6 seconds, and (b) difference of post-3s vs pre-3s period.**

**a**

| Items | Distancing | Distraction | Direct Inhibition | *F* (degrees of freedom) | *p* | *p*(FDR)^a^ | *η²* |
| --- | --- | --- | --- | --- | --- | --- | --- |
| **Auditory** | | | | | | | |
| DLPFC | 0.23 ± 0.68 | 0.06 ± 0.74 | 0.09 ± 0.45 | 1.13 (2,100) | 0.327 | 0.491 | 0.02 |
| PPC | 0.25 ± 1.33 | 0.02 ± 1.44 | 0.11 ± 0.93 | 0.67 (2,100) | 0.512 | 0.512 | 0.01 |
| STC | -0.39 ± 0.81 | -0.07 ± 0.73 | -0.07 ± 0.51 | 5.32 (2,100) | 0.006 | 0.018* | 0.10 |
| **Visual** | | | | | | | |
| DLPFC | 0.15 ± 1.65 | 0.10 ± 0.22 | 0.08 ± 0.70 | 0.16 (2,152) | 0.857 | 0.857 | 0.01 |
| PPC | 0.70 ± 1.30 | 0.28 ± 0.45 | 0.46 ± 0.91 | 5.50 (2,152) | 0.005 | 0.008** | 0.07 |
| VC | -0.79 ± 0.99 | -0.19 ± 0.64 | -1.12 ± 1.29 | -24.15 (2,152) | <0.001 | <0.001*** | 0.24 |

^a^ Corrected using false discovery rate correction method; NP, natural processing, IH, inhibition, **p*(FDR) < 0.05, ***p*(FDR) < 0.01, ****p*(FDR) < 0.001.

**b**

| Items | Distancing  (Post-3s - Pre-3s) | Distraction  (Post-3s - Pre-3s) | Direct Inhibition  (Post-3s - Pre-3s) | *F* (degrees of freedom) | *p* | *p*(FDR)^a^ | *η²* |
| --- | --- | --- | --- | --- | --- | --- | --- |
| **Auditory** | | | | | | | |
| DLPFC | -0.35 ± 0.98 | -0.10 ± 1.77 | -0.09 ± 0.67 | 0.73 (2,100) | 0.483 | 0.989 | 0.01 |
| PPC | -0.53± 2.40 | -0.34 ± 2.07 | -0.26 ± 1.57 | 0.40 (2,100) | 0.669 | 0.989 | 0.01 |
| STC | -0.16 ± 1.18 | -0.18 ± 0.94 | -0.19 ± 0.71 | 0.01 (2,100) | 0.989 | 0.989 | -0.45 |
| **Visual** | | | | | | | |
| DLPFC | 0.17 ± 0.57 | 0.19 ± 0.32 | 0.18 ± 0.67 | 0.06 (2,100) | 0.946 | 0.946 | 0.001 |
| PPC | 0.28 ± 1.49 | 0.18 ± 0.70 | 0.02 ± 1.32 | 1.63 (2,100) | 0.200 | 0.300 | 0.02 |
| VC | 0.18 ± 1.36 | 0.06 ± 0.78 | 0.12 ± 1.40 | 1.82 (2,100) | 0.166 | 0.300 | 0.02 |

**Table S3. Post-hoc beta values (*M ± SD*) of inhibition conjunction analysis** **for comparisons in Experiment 1: Analysis of stimulus exposure periods (a) 0-6 seconds, and (b)** **difference of post-3s vs pre-3s period.**

**a**

| Items | IH | NP | *t* (degrees of freedom) | *p* | *p*(FDR)^a^ | *Cohen’s d* |
| --- | --- | --- | --- | --- | --- | --- |
| **Auditory** | | | | | | |
| DLPFC | 0.08 ± 0.14 | -0.47 ± 0.42 | 9.60 (50) | < 0.001 | < 0.001*** | 1.34 |
| PPC | 0.20 ± 0.26 | -0.36 ± 0.84 | 4.97 (50) | < 0.001 | < 0.001*** | 0.70 |
| STC | 0.67 ± 0.39 | 1.48 ± 0.83 | -7.53 (50) | < 0.001 | < 0.001*** | -1.06 |
| **Visual** | | | | | | |
| DLPFC | 0.08 ± 0.19 | -0.16 ± 0.19 | 8.49 (76) | <0.001 | <0.001*** | 0.97 |
| PPC | 0.11 ± 0.28 | -0.17 ± 0.36 | 6.14 (76) | <0.001 | <0.001*** | 0.70 |
| VC | 0.30 ± 0.28 | 0.52 ± 0.33 | -5.38 (76) | <0.001 | <0.001*** | -0.61 |

^a^ Corrected using false discovery rate correction method; NP, natural processing, IH, inhibition, ****p*(FDR) < 0.001.

**b**

| Items | 0-3s | 3-6s | *t* (degrees of freedom) | *p* | *p*(FDR)^a^ | *Cohen’s d* |
| --- | --- | --- | --- | --- | --- | --- |
| **Auditory** | | | | | | |
| IH-increased DLPFC | 0.68 ± 0.66 | 0.65 ± 0.59 | 0.28 (50) | 0.784 | 0. 784 | 0.04 |
| IH-increased PPC | 0.76 ± 1.26 | 0.46 ± 1.12 | 2.11 (50) | 0.040 | 0.060 | 0.30 |
| IH-decreased STC | -0.76 ± 1.11 | -1.90 ± 1.23 | -6.96 (50) | <0.001 | <0.001*** | -0.98 |
| **Visual** | | | | | | |
| IH-increased DLPFC | 0.27 ± 0.40 | 0.21 ± 0.33 | 1.47 (76) | 0.147 | 0.147 | 0.17 |
| IH-increased PPC | 0.43 ± 0.65 | 0.22 ± 0.65 | 2.56 (76) | 0.012 | 0.018* | 0.29 |
| IH-decreased VC | -0.03 ± 0.71 | -0.37 ± 0.75 | -4.25 (76) | <0.001 | <0.001*** | -0.49 |

^a^ Corrected using false discovery rate correction method; NP, natural processing, IH, inhibition, **p*(FDR) < 0.05, ****p*(FDR) < 0.001..

**Table S4.** **Significant activation clusters of inhibition conjunction analysis within the combined ROI (DLPFC, PPC, STC/VC) in Experiment 1 (including the Auditory and Visual parts): Analysis of stimulus exposure periods (a) 0-6 seconds, and (b) difference of post-3s vs pre-3s period.**

**a.**

| Region | Laterality | Cluster size  (Voxels) | *T* | *Z* | *p*(FDR) | Peak MNI coordinates | | |
| --- | --- | --- | --- | --- | --- | --- | --- | --- |
|  |  |  |  |  |  | x | y | z |
| **Auditory, Contrast: IH > NP** | | | | | | | | |
| **DLPFC** | L | 775 | 11.57 | Inf | < 0.001 | -40 | 2 | 30 |
|  | R | 251 | 10.89 | 7.75 | < 0.001 | 8 | 30 | 38 |
|  | L | 340 | 10.06 | 7.40 | < 0.001 | -6 | 28 | 36 |
|  | R | 782 | 8.92 | 6.87 | < 0.001 | 46 | 30 | 20 |
| **PPC** | L | 943 | 10.25 | 7.49 | < 0.001 | -26 | -58 | 44 |
|  | R | 892 | 8.55 | 6.68 | < 0.001 | 28 | -58 | 44 |
|  | L | 17 | 6.20 | 5.32 | < 0.001 | -26 | -70 | 36 |
| **STC** | L | 12 | 2.88 | 2.76 | 0.008 | -44 | -58 | 14 |
| **Auditory, Contrast: IH < NP** | | | | | | | | |
| **PPC** | R | 32 | 5.20 | 4.63 | < 0.001 | 6 | -82 | 42 |
|  | R | 13 | 3.62 | 3.39 | 0.004 | 4 | -60 | 30 |
| **STC** | R | 482 | 11.26 | Inf | < 0.001 | 60 | -6 | 2 |
|  | L | 314 | 8.51 | 6.66 | < 0.001 | -62 | -12 | 4 |
| **Visual, Contrast: IH > NP** | | | | | | | | |
| **DLPFC** | L | 229 | 8.26 | 6.96 | < 0.001 | -8 | 26 | 36 |
|  | R | 198 | 8.10 | 6.86 | < 0.001 | 8 | 28 | 36 |
|  | L | 860 | 7.85 | 6.70 | < 0.001 | -42 | 2 | 32 |
|  | R | 942 | 6.96 | 6.10 | < 0.001 | 52 | 10 | 36 |
| **PPC** | L | 4033 | 10.85 | Inf | < 0.001 | -26 | -52 | 44 |
|  | L | 17 | 4.55 | 4.26 | < 0.001 | -26 | -70 | 36 |
| **Visual, Contrast: IH < NP** | | | | | | | | |
| **VC** | R | 48 | 7.21 | 6.28 | < 0.001 | 14 | -94 | -8 |
|  | L | 79 | 6.55 | 5.81 | < 0.001 | -14 | -98 | 0 |
|  | R | 202 | 6.54 | 5.81 | < 0.001 | 16 | -84 | -14 |
|  | R | 18 | 6.48 | 5.76 | < 0.001 | 16 | -90 | -2 |
|  | L | 51 | 6.30 | 5.64 | < 0.001 | -18 | -98 | 4 |
|  | R | 54 | 5.87 | 5.31 | < 0.001 | 24 | -92 | 2 |
|  | L | 113 | 5.63 | 5.13 | < 0.001 | -18 | -80 | -12 |
|  | R | 15 | 4.29 | 4.05 | 0.001 | 20 | -96 | 14 |
|  | R | 11 | 3.93 | 3.74 | 0.003 | 52 | -60 | -10 |

Data are thresholded at *p*(FDR) < 0.05, with a minimum cluster size of 10 voxels (For all analyses involving multiple comparisons, the *p*-values were further adjusted using an additional FDR correction). NP, natural processing, IH, inhibition, R: right. L: left.

**b.**

| Region | Laterality | Cluster size  (Voxels) | *T* | *Z* | *p*(FDR) | Peak MNI coordinates | | |
| --- | --- | --- | --- | --- | --- | --- | --- | --- |
|  |  |  |  |  |  | x | y | z |
| **Auditory, Contrast: IH > NP 0-3s** | | | | | | | | |
| **DLPFC** | L | 729 | 12.23 | Inf | < 0.001 | -44 | 12 | 30 |
|  | L | 256 | 10.33 | 7.52 | < 0.001 | -4 | 32 | 36 |
|  | R | 233 | 10.29 | 7.50 | < 0.001 | 4 | 30 | 38 |
|  | R | 801 | 9.38 | 7.09 | < 0.001 | 48 | 32 | 22 |
|  | L | 15 | 2.92 | 2.79 | 0.007 | -28 | 36 | 30 |
| **PPC** | L | 1062 | 8.52 | 6.66 | < 0.001 | -26 | -56 | 44 |
|  | R | 910 | 7.62 | 6.18 | < 0.001 | 28 | -58 | 44 |
|  | L | 17 | 6.08 | 5.24 | < 0.001 | -26 | -70 | 36 |
|  | R | 12 | 2.83 | 2.71 | 0.009 | 22 | -66 | 32 |
| **Auditory, Contrast: IH > NP 3-6s** | | | | | | | | |
| **DLPFC** | L | 648 | 10.18 | 7.46 | < 0.001 | -40 | 2 | 32 |
|  | R | 199 | 8.71 | 6.76 | < 0.001 | 8 | 30 | 38 |
|  | L | 372 | 8.39 | 6.60 | < 0.001 | -8 | 26 | 36 |
|  | R | 646 | 6.90 | 5.75 | < 0.001 | 46 | 30 | 24 |
|  | L | 13 | 5.67 | 4.96 | < 0.001 | -50 | 38 | 2 |
| **PPC** | L | 680 | 9.74 | 7.26 | < 0.001 | -26 | -60 | 46 |
|  | R | 685 | 7.88 | 6.33 | < 0.001 | 28 | -62 | 44 |
|  | L | 15 | 5.61 | 4.92 | < 0.001 | -26 | -70 | 36 |
| **Auditory, Contrast: Post 3s (IH > NP) – Pre 3s (IH > NP)** | | | | | | | | |
| No clusters survived | | | | | | | | |
| **Auditory, Contrast: IH < NP 0-3s** | | | | | | | | |
| **STC** | R | 319 | 8.63 | 6.72 | < 0.001 | 62 | -2 | -2 |
|  | L | 268 | 7.23 | 5.95 | < 0.001 | -62 | -8 | 0 |
| **Auditory, Contrast: IH < NP 3-6s** | | | | | | | | |
| **DLPFC** | R | 12 | 3.77 | 3.52 | 0.001 | 52 | 42 | 8 |
|  | R | 16 | 3.25 | 3.08 | 0.006 | 24 | 38 | 40 |
| **PPC** | L | 145 | 6.32 | 5.39 | < 0.001 | -4 | -78 | 34 |
|  | R | 262 | 6.25 | 5.35 | < 0.001 | 18 | -84 | 34 |
|  | R | 84 | 4.48 | 4.09 | < 0.001 | 6 | -48 | 60 |
|  | L | 55 | 4.46 | 4.07 | < 0.001 | -20 | -40 | 48 |
| **STC** | R | 680 | 12.76 | Inf | < 0.001 | 64 | -18 | 0 |
|  | L | 513 | 11.67 | Inf | < 0.001 | -48 | -18 | 6 |
|  | R | 15 | 5.17 | 4.60 | < 0.001 | 54 | -60 | 14 |
|  | R | 10 | 3.87 | 3.60 | 0.001 | 42 | -58 | 14 |
| **Auditory, Contrast: Post 3s (IH < NP) – Pre 3s (IH < NP)** | | | | | | | | |
| **DLPFC** | R | 42 | 4.20 | 4.11 | < 0.001 | 46 | 30 | 12 |
|  | R | 189 | 4.09 | 4.01 | 0.001 | 46 | 24 | 42 |
|  | R | 38 | 3.80 | 3.73 | 0.001 | 52 | 42 | 6 |
|  | R | 37 | 3.67 | 3.60 | 0.002 | 20 | 40 | 36 |
|  | L | 12 | 3.48 | 3.43 | 0.003 | -48 | 40 | 16 |
|  | L | 15 | 3.39 | 3.34 | 0.004 | -22 | 38 | 38 |
|  | L | 21 | 3.10 | 3.06 | 0.008 | -30 | 22 | 40 |
|  | L | 15 | 2.94 | 2.90 | 0.012 | -2 | 34 | 34 |
|  | R | 10 | 2.90 | 2.86 | 0.013 | 4 | 38 | 30 |
| **PPC** | R | 279 | 4.80 | 4.66 | < 0.001 | 18 | -82 | 36 |
|  | L | 170 | 4.49 | 4.37 | < 0.001 | -16 | -82 | 36 |
|  | L | 296 | 4.17 | 4.08 | < 0.001 | -16 | -42 | 54 |
|  | R | 116 | 4.09 | 4.01 | 0.001 | 10 | -52 | 48 |
|  | R | 22 | 3.67 | 3.60 | 0.002 | 42 | -64 | 52 |
| **STC** | R | 602 | 8.61 | Inf | < 0.001 | 52 | -12 | 4 |
|  | L | 317 | 8.11 | 7.53 | < 0.001 | -48 | -18 | 6 |
| **Visual, Contrast: IH > NP 0-3s** | | | | | | | | |
| **DLPFC** | R | 239 | 9.24 | 7.54 | < 0.001 | 8 | 30 | 38 |
|  | L | 908 | 9.13 | 7.48 | < 0.001 | -48 | 16 | 28 |
|  | R | 1116 | 8.93 | 7.36 | < 0.001 | 36 | 6 | 40 |
|  | L | 256 | 8.38 | 7.03 | < 0.001 | -6 | 28 | 36 |
|  | L | 20 | 3.23 | 3.12 | 0.002 | -18 | 38 | 36 |
| **PPC** | L | 4466 | 11.21 | Inf | < 0.001 | -26 | -54 | 44 |
|  | L | 17 | 5.35 | 4.92 | < 0.001 | -26 | -70 | 36 |
|  | L | 76 | 4.28 | 4.04 | < 0.001 | -36 | -88 | -14 |
|  | R | 54 | 2.47 | 2.42 | 0.015 | 4 | -40 | 52 |
| **VC** | L | 13 | 3.56 | 3.41 | 0.001 | -48 | -66 | 20 |
| **Visual, Contrast: IH > NP 3-6s** | | | | | | | | |
| **DLPFC** | R | 429 | 6.27 | 5.61 | < 0.001 | 52 | 8 | 38 |
|  | R | 69 | 6.07 | 5.47 | < 0.001 | 10 | 28 | 34 |
|  | L | 568 | 5.92 | 5.35 | < 0.001 | -42 | 2 | 32 |
|  | L | 60 | 5.47 | 5.01 | < 0.001 | -8 | 26 | 36 |
|  | R | 31 | 3.17 | 3.06 | 0.007 | 12 | 40 | 20 |
|  | L | 10 | 2.83 | 2.75 | 0.015 | -42 | 42 | 10 |
|  | L | 11 | 2.46 | 2.41 | 0.032 | -30 | 42 | 30 |
| **PPC** | L | 743 | 6.49 | 5.77 | < 0.001 | -8 | -68 | 48 |
|  | R | 323 | 4.96 | 4.60 | < 0.001 | 12 | -66 | 46 |
|  | R | 36 | 2.91 | 2.83 | 0.012 | 22 | -54 | 44 |
| **VC** | R | 1186 | 9.56 | 7.72 | < 0.001 | -4 | -74 | 2 |
| **Visual, Contrast: Post 3s (IH > NP) – Pre 3s (IH > NP)** | | | | | | | | |
| **DLPFC** | L | 53 | 7.42 | 7.11 | < 0.001 | -54 | 8 | 40 |
|  | L | 19 | 6.12 | 5.94 | < 0.001 | -10 | 60 | 36 |
|  | R | 11 | 5.91 | 5.75 | < 0.001 | 62 | 14 | 26 |
|  | L | 14 | 4.36 | 4.29 | 0.001 | -42 | 16 | 34 |
| **PPC** | L | 18 | 6.85 | 6.60 | < 0.001 | -24 | -56 | 46 |
| **VC** | R | 18 | 6.22 | 6.03 | < 0.001 | 6 | -74 | 0 |
|  | L | 17 | 4.86 | 4.77 | < 0.001 | -6 | -74 | -4 |
|  | R | 10 | 3.39 | 3.35 | 0.032 | 6 | -84 | 10 |
| **Visual, Contrast: IH < NP 0-3s** | | | | | | | | |
| **DLPFC** | L | 14 | 4.17 | 3.94 | 0.007 | -44 | 18 | 28 |
|  | L | 25 | 3.82 | 3.64 | 0.016 | -2 | 56 | 40 |
|  | L | 11 | 3.33 | 3.21 | 0.049 | -58 | 32 | 12 |
| **PPC** | L | 28 | 3.82 | 3.64 | 0.016 | -24 | -44 | 70 |
| **VC** | L | 119 | 5.80 | 5.26 | < 0.001 | -36 | -88 | -14 |
|  | L | 13 | 3.98 | 3.78 | 0.011 | -32 | -94 | -4 |
| **Visual, Contrast: IH < NP 3-6s** | | | | | | | | |
| **VC** | L | 430 | 7.52 | 6.48 | < 0.001 | -16 | -96 | 0 |
|  | L | 203 | 7.50 | 6.47 | < 0.001 | -16 | -100 | 2 |
|  | R | 473 | 7.00 | 6.13 | < 0.001 | 12 | -94 | -8 |
|  | R | 66 | 6.87 | 6.04 | < 0.001 | 28 | -94 | 4 |
|  | R | 15 | 6.40 | 5.71 | < 0.001 | 20 | -88 | -2 |
|  | R | 24 | 5.14 | 4.75 | < 0.001 | 52 | -66 | 12 |
|  | R | 37 | 4.72 | 4.41 | < 0.001 | 24 | -96 | 14 |
|  | R | 16 | 4.44 | 4.17 | < 0.001 | 54 | -66 | -6 |
|  | R | 13 | 3.86 | 3.68 | 0.002 | 46 | -80 | 10 |
| **VIC, Contrast: Post 3s (IH < NP) – Pre 3s (IH < NP)** | | | | | | | | |
| **PPC** | L | 53 | 6.14 | 5.96 | < 0.001 | -22 | -86 | 28 |
|  | R | 49 | 5.70 | 5.56 | < 0.001 | 20 | -78 | 38 |
|  | R | 10 | 5.39 | 5.26 | < 0.001 | 30 | -84 | 26 |
|  | L | 13 | 5.10 | 4.99 | < 0.001 | -28 | -88 | 20 |
|  | L | 14 | 4.89 | 4.79 | < 0.001 | -28 | -94 | 2 |
|  | R | 11 | 4.21 | 4.14 | 0.001 | 20 | -66 | 32 |
| **VC** | L | 58 | 7.41 | 7.10 | < 0.001 | -14 | -84 | -16 |
|  | R | 113 | 7.07 | 6.80 | < 0.001 | 22 | -74 | -12 |
|  | L | 13 | 6.34 | 6.14 | < 0.001 | -26 | -54 | -8 |
|  | R | 10 | 5.92 | 5.75 | < 0.001 | 24 | -56 | -8 |

Data are thresholded at *p*(FDR) < 0.05, with a minimum cluster size of 10 voxels (For all analyses involving multiple comparisons, the *p*-values were further adjusted using an additional FDR correction). NP, natural processing, IH, inhibition, R: right. L: left.

**Table S5. Correlations between participants’ inhibition-related behavioral efficacy and inhibition-related brain activation or deactivation (from Experiment 1).**

| Items | *r* | *p* | *p*(FDR)^a^ |
| --- | --- | --- | --- |
| **Auditory** | | | |
| IH-decreased *P*_sensed_ & IH-increased DLPFC activity | -0.261 | 0.064 | 0.115 |
| IH-decreased *P*_sensed_ & IH-increased PPC activity | -0.016 | 0.732 | 0.732 |
| IH-decreased *P*_sensed_ & IH-decreased STC activity | 0.285 | 0.043 | 0.104 |
| IH-decreased *P*_understood_ & IH-increased DLPFC activity | -0.409 | 0.003 | 0.014* |
| IH-decreased *P*_understood_ & IH-increased PPC activity | -0.184 | 0.197 | 0.296 |
| IH-decreased *P*_understood_ & IH-decreased STC activity | 0.604 | <0.001 | <0.001*** |
| IH-decreased *ACC*_objective_ & IH-increased DLPFC activity | -0.107 | 0.456 | 0.586 |
| IH-decreased *ACC*_objective_ & IH-increased PPC activity | 0.091 | 0.528 | 0.594 |
| IH-decreased *ACC*_objective_ & IH-decreased STC activity | 0.280 | 0.046 | 0.104 |
| **Visual** | | | |
| IH-decreased *P*_sensed_ & IH-increased DLPFC activity | -0.206 | 0.073 | 0.164 |
| IH-decreased *P*_sensed_ & IH-increased PPC activity | -0.002 | 0.986 | 0.986 |
| IH-decreased *P*_sensed_ & IH-decreased VC activity | 0.057 | 0.622 | 0.800 |
| IH-decreased *P*_understood_ & IH-increased DLPFC activity | -0.289 | 0.011 | 0.036* |
| IH-decreased *P*_understood_ & IH-increased PPC activity | -0.024 | 0.834 | 0.938 |
| IH-decreased *P*_understood_ & IH-decreased VC activity | 0.310 | 0.006 | 0.036* |
| IH-decreased *ACC*_objective_ & IH-increased DLPFC activity | -0.140 | 0.224 | 0.336 |
| IH-decreased *ACC*_objective_ & IH-increased PPC activity | -0.284 | 0.012 | 0.036* |
| IH-decreased *ACC*_objective_ & IH-decreased VC activity | 0.163 | 0.157 | 0.282 |

^a^ Corrected using false discovery rate correction method; NP, natural processing, IH, inhibition, **p*(FDR) < 0.05, ****p*(FDR) < 0.001.

**Table S6. Post-hoc beta values (*M ± SD*) for comparisons between sensory areas and DMN in Experiment 1: Analysis of stimulus exposure periods (a) 0-6 seconds, and (b) difference of post-3s vs pre-3s period.**

**a**

| Items | STC/VC | DMN | *t* (degrees of freedom) | *p* | *p*(FDR)^a^ | *Cohens’ d* |
| --- | --- | --- | --- | --- | --- | --- |
| Auditory | -0.81 ± 0.76 | -0.14 ± 0.35 | -6.09 (50) | <0.001 | < 0.001*** | -0.85 |
| Visual | -0.22 ± 0.36 | -0.10 ± 0.19 | -2.45 (76) | 0.017 | 0.017* | -0.28 |

^a^ Corrected using false discovery rate correction method; **p*(FDR) < 0.05, ****p*(FDR) < 0.001.

**b**

| Items | 0-3 | 3-6 | *t* (degrees of freedom) | *p* | *p*(FDR)^a^ | *Cohens’ d* |
| --- | --- | --- | --- | --- | --- | --- |
| **Auditory** | | | | | | |
| DMN | -0.17 ± 0.64 | -0.24 ± 0.4466 | -0.53 (50) | 0.601 | 0.601 | -0.05 |
| STC | -0.76 ± 1.11 | -1.90 ± 1.23 | -6.96 (50) | <0.001 | < 0.001*** | -0.93 |
| **Visual** | | | | | | |
| DMN | -0.05 ± 0.34 | -0.18 ± 0.28 | -3.33 (76) | 0.001 | 0.001** | -0.17 |
| VC | -0.03 ± 0.71 | -0.37 ± 0.75 | -4.25 (76) | <0.001 | < 0.001*** | -0.43 |

**Table S7.** DCM Results. Table a shows the summary of Bayesian model selection. Table b shows the results from the auditory experiment, and Table c shows the results from the visual experiment.

**a**

| Model | Model Evidence (Summed Free Energy, F) | Expected Probability (EP) | Protected Exceedance Probability (PEP) | Bayesian Omnibus Risk (BOR) |
| --- | --- | --- | --- | --- |
| **Auditory** | | | | |
| **M1** | -4942.4 | 0.54 | 0.9961 | < 0.001 |
| **M2** | -4966.8 | 0.03 | 5.32E-10 |  |
| **M3** | -4969.68 | 0.03 | 5.32E-10 |  |
| **M4** | -4965.85 | 0.06 | 0.0001 |  |
| **M5** | -4971.36 | 0.03 | 5.32E-10 |  |
| **M6** | -4968.51 | 0.06 | 0.0001 |  |
| **M7** | -4968.5 | 0.03 | 5.32E-10 |  |
| **M8** | -4967.95 | 0.04 | 5.32E-10 |  |
| **M9** | -4968.74 | 0.04 | 5.32E-10 |  |
| **M10** | -4962.05 | 0.15 | 0.0037 |  |
| **Visual** | | | | |
| **M1** | -5936.45 | 0.63 | 1 | < 0.001 |
| **M2** | -5988.42 | 0.03 | 6.41E-13 |  |
| **M3** | -5992.58 | 0.03 | 6.41E-13 |  |
| **M4** | -5973.48 | 0.06 | 6.41E-13 |  |
| **M5** | -5993.26 | 0.03 | 6.41E-13 |  |
| **M6** | -5991.06 | 0.03 | 6.41E-13 |  |
| **M7** | -5993.45 | 0.03 | 6.41E-13 |  |
| **M8** | -5993.08 | 0.03 | 6.41E-13 |  |
| **M9** | -5989.28 | 0.05 | 6.41E-13 |  |
| **M10** | -5977.29 | 0.08 | 6.41E-13 |  |

**b**

| Items | Auditory (N = 32) | | |  |
| --- | --- | --- | --- | --- |
|  | Mean ± SE (Hz) | *t* | *p(FDR)* |  |
| **Intrinsic connectivity** |  |  |  |  |
| DLPFC to DLPFC | -0.08 ± 0.02 | -3.45 | 0.005** |  |
| DLPFC to STC | 0.04 ± 0.04 | 1.08 | 0.595 |  |
| DLPFC to DMN | 0.03 ± 0.05 | 0.64 | 0.682 |  |
| STC to DLPFC | 0.02 ± 0.03 | 0.50 | 0.698 |  |
| STC to STC | -0.12 ± 0.03 | -4.75 | < 0.001*** |  |
| STC to DMN | 0.04 ± 0.04 | 0.99 | 0.595 |  |
| DMN to DLPFC | 0.01 ± 0.03 | 0.30 | 0.763 |  |
| DMN to STC | 0.03 ± 0.04 | 0.70 | 0.682 |  |
| DMN to DMN | -0.12 ± 0.02 | -5.00 | < 0.001*** |  |
| **Modulation by IH** |  |  |  |  |
| DLPFC to STC | -0.68 ± 0.20 | -3.34 | 0.013* |  |
| DLPFC to DMN | -0.19 ± 0.13 | −1.46 | 0.235 |  |
| STC to DLPFC | -0.12 ± 0.11 | −1.20 | 0.269 |  |
| STC to DMN | -0.10 ± 0.09 | -1.13 | 0.269 |  |
| DMN to DLPFC | 0.25 ± 0.17 | 1.45 | 0.235 |  |
| DMN to STC | 0.44 ± 0.22 | 1.99 | 0.167 |  |
| **Driving input** |  |  |  |  |
| to DLPFC | 0.01 ± 0.04 | 0.15 | 0.880 |  |
| to STC | 0.07 ± 0.05 | 1.50 | 0.216 |  |
| to DMN | -0.01 ± 0.03 | -0.36 | 0.863 |  |

^a^ Corrected using false discovery rate correction method; NP, natural processing, IH, inhibition, **p*(FDR) < 0.05, ***p*(FDR) < 0.01, ****p*(FDR) < 0.001.

**c**

| Items | Visual (N = 25) | | |  |
| --- | --- | --- | --- | --- |
|  | Mean ± SE (Hz) | *t* | *p(FDR)* |  |
| **Intrinsic connectivity** | | | | |
| DLPFC to DLPFC | -0.17 ± 0.05 | -3.83 | 0.003** |  |
| DLPFC to VC | 0.13 ± 0.04 | 3.19 | 0.009** |  |
| DLPFC to DMN | 0.09 ± 0.06 | 1.48 | 0.171 |  |
| VC to DLPFC | 0.07 ± 0.03 | 2.30 | 0.046* |  |
| VC to VC | -0.17 ± 0.04 | -3.79 | 0.003** |  |
| VC to DMN | -0.02 ± 0.04 | -0.36 | 0.719 |  |
| DMN to DLPFC | 0.16 ± 0.07 | 2.44 | 0.040* |  |
| DMN to VC | -0.12 ± 0.08 | -1.64 | 0.148 |  |
| DMN to DMN | -0.13 ± 0.02 | -5.06 | < 0.001 |  |
| **Modulation by IH** | | | |  |
| DLPFC to VC | -2.24 ± 0.29 | -7.64 | < 0.001*** |  |
| DLPFC to DMN | -1.09 ± 0.14 | -7.92 | < 0.001*** |  |
| VC to DLPFC | -0.02 ± 0.07 | −0.24 | 0.940 |  |
| VC to DMN | 0.01 ± 0.09 | 0.08 | 0.940 |  |
| DMN to DLPFC | 0.03 ± 0.13 | 0.21 | 0.940 |  |
| DMN to VC | 1.71 ± 0.25 | 6.94 | < 0.001*** |  |
| **Driving input** | | | | |
| to DLPFC | 0.07 ± 0.04 | 1.78 | 0.105 |  |
| to VC | 0.32 ± 0.10 | 3.11 | 0.010* |  |
| to DMN | 0.03 ± 0.06 | 0.41 | 0.682 |  |

^a^ Corrected using false discovery rate correction method; NP, natural processing, IH, inhibition, **p*(FDR) < 0.05, ***p*(FDR) < 0.01, ****p*(FDR) < 0.001.

**Table S8.** Results of EEG source localization. Clusters with significant activation differences between NP and IH in ROIs.

| Region | Laterality | Significant duration | Cluster size  (Voxels) | *p*(FDR) | Centeral MNI coordinates | | |
| --- | --- | --- | --- | --- | --- | --- | --- |
|  |  |  |  |  | x | y | z |
| DLPFC | L | 6 | 28 | <0.001 | -42 | 26 | 32 |
| DLPFC | L | 6 | 33 | <0.001 | -37 | 26 | 34 |
| DLPFC | L | 6 | 10 | <0.001 | -36 | 30 | 34 |
| DLPFC | R | 6 | 26 | <0.001 | 35 | 32 | 30 |
| DLPFC | R | 6 | 23 | <0.001 | 39 | 28 | 27 |
| DLPFC | R | 6 | 13 | <0.001 | 37 | 32 | 30 |
| PPC | L | 6 | 29 | < 0.001 | -20 | -54 | 59 |
| PPC | L | 6 | 26 | < 0.001 | -16 | -56 | 58 |
| PPC | R | 6 | 29 | < 0.001 | 20 | -51 | 59 |
| PPC | R | 6 | 21 | < 0.001 | 17 | -53 | 59 |
| PPC | R | 6 | 12 | < 0.001 | 18 | -52 | 59 |
| PPC | R | 6 | 17 | < 0.001 | 19 | -56 | 59 |

**Table S9. Correlations between participants’ inhibition-related behavioral efficacy and the post-3s vs. pre-3s differences of inhibition-related activation or deactivation (from Experiment 1).**

| Items | *r* | *p* | *p*(FDR)^a^ |
| --- | --- | --- | --- |
| **Auditory** | | | |
| IH-decreased *P*_sensed_ & DLPFC high-to-low activation | -0.030 | 0.835 | 0.979 |
| IH-decreased *P*_sensed_ & PPC high-to-low activation | -0.025 | 0.860 | 0.979 |
| IH-decreased *P*_sensed_ & STC low-to-high deactivation | 0.191 | 0.179 | 0.806 |
| IH-decreased *P*_understood_ & DLPFC high-to-low activation | 0.035 | 0.806 | 0.979 |
| IH-decreased *P*_understood_ & PPC high-to-low activation | -0.033 | 0.817 | 0.979 |
| IH-decreased *P*_understood_ & STC low-to-high deactivation | 0.454 | <0.001 | <0.001*** |
| IH-decreased *ACC*_objective_ & DLPFC high-to-low activation | 0.004 | 0.979 | 0.979 |
| IH-decreased *ACC*_objective_ & PPC high-to-low activation | -0.108 | 0.451 | 0.979 |
| IH-decreased *ACC*_objective_ & STC low-to-high deactivation | 0.011 | 0.938 | 0.979 |
| **Visual** | | | |
| IH-decreased *P*_sensed_ & DLPFC high-to-low activation | -0.071 | 0.540 | 0.673 |
| IH-decreased *P*_sensed_ & PPC high-to-low activation | 0.093 | 0.419 | 0.628 |
| IH-decreased *P*_sensed_ & VC low-to-high deactivation | -0.101 | 0.382 | 0.628 |
| IH-decreased *P*_understood_ & DLPFC high-to-low activation | -0.061 | 0.599 | 0.975 |
| IH-decreased *P*_understood_ & PPC high-to-low activation | 0.106 | 0.359 | 0.673 |
| IH-decreased *P*_understood_ & VC low-to-high deactivation | 0.404 | <0.001 | <0.001*** |
| IH-decreased *ACC*_objective_ & DLPFC high-to-low activation | -0.170 | 0.139 | 0.417 |
| IH-decreased *ACC*_objective_ & PPC high-to-low activation | 0.046 | 0.692 | 0.692 |
| IH-decreased *ACC*_objective_ & VC low-to-high deactivation | 0.225 | 0.049 | 0.221 |

^a^ Corrected using false discovery rate correction method; NP, natural processing, IH, inhibition, **p*(FDR) < 0.05.

**Table S10. Descriptive statistics of the cross-modal (auditory and visual) decoding analysis for the decoding accuracy (*M ± SD*) of each condition across all participants in Experiments 1.** The decoding accuracy for each condition was statistically tested against chance level (0.5) using one-sample *t*-test.

| Brain region | 0-6 s | | | 0-3 s | | | 3-6 s | | |
| --- | --- | --- | --- | --- | --- | --- | --- | --- | --- |
|  | NP | IH | AUC | NP | IH | AUC | NP | IH | AUC |
| **Auditory modality as training set, visual modality as testing set** | | | | | | | | | |
| Frontoparietal region | 0.45 ± 0.16 | 0.65 ± 0.18*** | 0.69 ± 0.02*** | 0.49 ± 0.12 | 0.62 ± 0.14*** | 0.67 ± 0.02*** | 0.42 ± 0.12 | 0.67 ± 0.13*** | 0.70 ± 0.02*** |
| **Visual modality as training set, auditory modality as testing set** | | | | | | | | | |
| Frontoparietal region | 0.49 ± 0.14 | 0.56 ± 0.16^†^ | 0.61 ± 0.02*** | 0.53 ± 0.12 | 0.54 ± 0.11 | 0.60 ± 0.02*** | 0.49 ± 0.11 | 0.58 ± 0.13** | 0.64 ± 0.02*** |

^†^*p*(FDR) marginally significant, NP, natural processing, IH, inhibition, ***p*(FDR) < 0.01, ****p*(FDR) < 0.001.

**Table S11. Descriptive statistics of cross-modal decoding analysis decoding accuracies (*M* ± *SD*) in Experiment 1: Analysis of stimuli exposure periods (a) 0-6 seconds, and (b) difference of post-3s vs. pre-3s period.**

| Items | IH | NP | *t* (degrees of freedom) | *p* | *p*(FDR)^a^ | Cohen’s *d* |
| --- | --- | --- | --- | --- | --- | --- |
| **Auditory modality as training set, visual modality as testing set** | | | | | | |
| Frontoparietal region | 0.65 ± 0.18 | 0.45 ± 0.16 | 3.11 (26) | 0.004 | 0.008** | 0.60 |
| **Visual modality as training set, auditory modality as testing set** | | | | | | |
| Frontoparietal region | 0.56 ± 0.16 | 0.49 ± 0.14 | 1.32 (31) | 0.197 | 0.197 | 0.23 |

**a**

^a^ Corrected using false discovery rate correction method; NP, natural processing, IH, inhibition, ***p*(FDR) < 0.01.

**b**

| Items | 0-3s | 3-6s | *t* (degrees of freedom) | *p* | *p*(FDR)^a^ | Cohen’s *d* |
| --- | --- | --- | --- | --- | --- | --- |
| **Auditory modality as training set, visual modality as testing set** | | | | | | |
| Frontoparietal region (IH>NP) | 0.13 ± 0.23 | 0.25 ± 0.23 | 2.61 (26) | 0.015 | 0.030* | 0.50 |
| **Visual modality as training set, auditory modality as testing set** | | | | | | |
| Frontoparietal region (IH>NP) | 0.01 ± 0.21 | 0.10 ± 0.21 | 1.97 (31) | 0.057 | 0.057^†^ | 0.35 |

^a^ Corrected using false discovery rate correction method; NP, natural processing, IH, inhibition, ^†^p(FDR) marginally significant, **p*(FDR) < 0.05.

**Table S12. Descriptive statistics of the unimodal MVPA for the decoding accuracy (*M ± SD*) of each condition across all participants in Experiments 1.** **(a)** The decoding accuracy using smoothed data for each condition was statistically tested against chance level (0.5) using one-sample *t*-test. **(b)** The decoding accuracy using unsmoothed visual data for each condition was statistically tested against chance level (0.5) using one-sample *t*-test.

**a**

| Brain region | 0-6 s | | | 0-3 s | | | 3-6 s | | |
| --- | --- | --- | --- | --- | --- | --- | --- | --- | --- |
|  | NP | IH | AUC | NP | IH | AUC | NP | IH | AUC |
| **Auditory** | | | | | | | | | |
| DLPFC | 0.54 ± 0.13 | 0.61 ± 0.16** | 0.61 ± 0.03** | 0.52 ± 0.14 | 0.64 ± 0.14*** | 0.68 ±0.04*** | 0.56 ± 0.17 | 0.58 ± 0.16* | 0.63 ± 0.03*** |
| PPC | 0.57 ± 0.15* | 0.58 ± 0.14** | 0.65 ± 0.04*** | 0.58 ± 0.13** | 0.6 ± 0.16** | 0.63 ± 0.04*** | 0.56 ± 0.17* | 0.57 ± 0.15* | 0.67 ± 0.04*** |
| STC | 0.62 ± 0.13*** | 0.57 ± 0.12** | 0.66 ± 0.04*** | 0.58 ± 0.12** | 0.58 ± 0.13** | 0.60 ± 0.04** | 0.66 ± 0.16*** | 0.55 ± 0.16 | 0.60 ± 0.04** |
| **Visual** | | | | | | | | | |
| DLPFC | 0.66 ± 0.14*** | 0.70 ± 0.11*** | 0.83 ± 0.04*** | 0.63 ± 0.18*** | 0.70 ± 0.16*** | 0.77 ± 0.04*** | 0.63 ± 0.18*** | 0.70 ± 0.16*** | 0.81 ± 0.03*** |
| PPC | 0.66 ± 0.15*** | 0.70 ± 0.11*** | 0.84 ± 0.03*** | 0.66 ± 0.17*** | 0.69 ± 0.16*** | 0.85 ± 0.03*** | 0.69 ± 0.17*** | 0.66 ± 0.14*** | 0.87 ± 0.04*** |
| VC | 0.70 ± 0.16*** | 0.67 ± 0.16*** | 0.88 ± 0.04*** | 0.68 ± 0.18*** | 0.67 ± 0.19*** | 0.81 ± 0.04*** | 0.74 ± 0.17*** | 0.66 ± 0.16*** | 0.87 ± 0.04*** |

NP, natural processing, IH, inhibition, **p*(FDR) < 0.05, ***p*

**b**

| Brain region | 0-6 s | |
| --- | --- | --- |
|  | NP | IH |
| DLPFC | 0.65 ± 0.19*** | 0.71 ± 0.15*** |
| PPC | 0.68 ± 0.21*** | 0.66 ± 0.20*** |
| VC | 0.68 ± 0.20*** | 0.71 ± 0.15*** |

**Table S13. Results of unimodal MVPA decoding accuracies (*M ± SD*) in Experiment 1: Analysis of stimulus exposure periods (a) 0-6 seconds** **using smoothed data; (b) a comparison between smoothed and unsmoothed visual data for the 0-6 second period on visual data and (c) difference of post-3s vs. pre-3s period using smoothed data.**

**a**

| Items | IH | NP | *t* (degrees of freedom) | *p* | *p*(FDR)^a^ | *Cohen’s d* |
| --- | --- | --- | --- | --- | --- | --- |
| **Auditory** | | | | | | |
| DLPFC | 0.61 ± 0.16 | 0.54 ± 0.13 | 2.30 (31) | 0.028 | 0.042* | 0.41 |
| PPC | 0.58 ± 0.14 | 0.57 ± 0.15 | 0.57 (31) | 0.572 | 0.572 | 0.10 |
| STC | 0.57 ± 0.12 | 0.62 ± 0.13 | -2.66 (31) | 0.012 | 0.036* | -0.47 |
| **Visual** | | | | | | |
| DLPFC | 0.70 ± 0.11 | 0.66 ± 0.14 | 2.31 (26) | 0.029 | 0.029* | 0.44 |
| PPC | 0.70 ± 0.11 | 0.66 ± 0.15 | 2.34 (26) | 0.027 | 0.029* | 0.45 |
| VC | 0.67 ± 0.15 | 0.71 ± 0.16 | -2.53 (26) | 0.018 | 0.029* | -0.49 |

^a^ Corrected using false discovery rate correction method; NP, natural processing, IH, inhibition, **p*(FDR) < 0.05.

**b**

| Items | Smooth | Unsmooth | *t* (degrees of freedom) | *p* | *p*(FDR)^a^ | *Cohen’s d* |
| --- | --- | --- | --- | --- | --- | --- |
| NP | | | | | | |
| DLPFC | 0.66 ± 0.14 | 0.65 ± 0.19 | 0.24 (26) | 0.810 | 0.879 | 0.05 |
| PPC | 0.66 ± 0.15 | 0.68 ± 0.21 | -0.84 (26) | 0.410 | 0.616 | -0.16 |
| VC | 0.71 ± 0.16 | 0.68 ± 0.20 | 1.34 (26) | 0.192 | 0.385 | 0.26 |
| IH | | | | | | |
| DLPFC | 0.70 ± 0.11 | 0.71 ± 0.15 | 0.15 (26) | 0.879 | 0.879 | 0.03 |
| PPC | 0.70 ± 0.11 | 0.66 ± 0.20 | 1.55 (26) | 0.134 | 0.385 | 0.30 |
| VC | 0.67 ± 0.15 | 0.71 ± 0.15 | -1.67 (26) | 0.106 | 0.385 | -0.32 |

**c**

| Items | 0-3s | 3-6s | *t* (degrees of freedom) | *p* | *p*(FDR)^a^ | *Cohen’s d* |
| --- | --- | --- | --- | --- | --- | --- |
| **Auditory** | | | | | | |
| IH-increased DLPFC | 0.10 ± 0.13 | 0.02 ± 0.20 | -2.12 (31) | 0.043 | 0.065^†^ | -0.37 |
| IH-increased PPC | 0.02 ± 0.17 | 0.01 ± 0.19 | -0.42 (31) | 0.676 | 0.676 | -0.07 |
| IH-decreased STC | -0.0007 ± 0.13 | -0.11 ± 0.17 | -3.32 (31) | 0.002 | 0.006** | -0.59 |
| **Visual** | | | | | | |
| IH-increased DLPFC | 0.07 ± 0.10 | -0.002 ± 0.14 | -2.37 (26) | 0.026 | 0.043* | -0.46 |
| IH-increased PPC | 0.03 ± 0.11 | -0.03 ± 0.10 | -2.13 (26) | 0.043 | 0.043* | -0.41 |
| IH-decreased VC | -0.01 ± 0.11 | -0.08 ± 0.11 | -2.19 (26) | 0.038 | 0.043* | -0.42 |

^a^ Corrected using false discovery rate correction method; NP, natural processing, IH, inhibition, ^†^*p*(FDR) marginally significant, **p*(FDR) < 0.05, ***p*(FDR) < 0.01. (FDR) < 0.01, ****p*(FDR) < 0.00.

**Table S14. Descriptive statistics of feature MVPA decoding accuracies (*M ± SD*) in Experiment 1.** The decoding accuracy for each condition was statistically tested against chance level (0.25) using one-sample *t*-test.

| Items | NP | | | | IH | | | |
| --- | --- | --- | --- | --- | --- | --- | --- | --- |
|  | high | high AUC | low | low AUC | high | high AUC | low | low AUC |
| Valence | 0.41 ± 0.11*** | 0.64 ± 0.13*** | 0.36 ± 0.12*** | 0.64 ± 0.12*** | 0.34 ± 0.08*** | 0.62 ± 0.09*** | 0.34 ± 0.09*** | 0.62 ± 0.08*** |
| Arousal | 0.41 ± 0.08*** | 0.65 ± 0.11*** | 0.42 ± 0.11*** | 0.66 ± 0.11*** | 0.34 ± 0.08*** | 0.62 ± 0.09*** | 0.31 ± 0.07*** | 0.60 ± 0.09*** |
| Information load | 0.35 ± 0.08*** | 0.69 ± 0.13*** | 0.31 ± 0.07*** | 0.72 ± 0.11*** | 0.44 ± 0.13*** | 0.63 ± 0.09*** | 0.47 ± 0.14*** | 0.60 ± 0.10*** |
| Difficulty | 0.36 ± 0.09*** | 0.57 ± 0.09** | 0.30 ± 0.08** | 0.63 ± 0.10*** | 0.33 ± 0.09*** | 0.63 ± 0.09*** | 0.35 ± 0.10*** | 0.60 ± 0.10*** |

NP, natural processing, IH, inhibition, ***p*(FDR) < 0.01, ****p*(FDR) < 0.001

**Table S15. Post-hoc beta values (*M ± SD*) for inhibition vs. NP pre-cTBS and inhibition vs. NP post-cTBS conditions for comparisons in Experiment 4: Analysis of stimulus exposure periods (a) 0-6 seconds, and (b) difference of post-3s vs. pre-3s period.**

**a**

| Items | IH vs. NP pre-cTBS | IH vs. NP post-cTBS | *t* (degrees of freedom) | *p* | *p*(FDR)^a^ | *Cohen’s d* |
| --- | --- | --- | --- | --- | --- | --- |
| DLPFC | 0.58 ± 0.29 | 0.37 ± 0.32 | 3.77 (35) | <0.001 | <0.001*** | 0.63 |
| PPC | 0.71 ± 0.49 | 0.27 ± 0.60 | 3.90 (35) | <0.001 | <0.001*** | 0.65 |
| STC | -0.56 ± 0.49 | -0.38 ± 0.51 | -2.40 (35) | 0.022 | 0.022* | -0.40 |

^a^ Corrected using false discovery rate correction method; NP, natural processing, IH, inhibition, **p*(FDR) < 0.05, ****p*(FDR) < 0.001.

**b**

| Items | Post-3s(IH vs. NP pre-cTBS) -  Pre-3s(IH vs. NP pre-cTBS) | Post-3s(IH vs. NP post-cTBS) -  Pre-3s(IH vs. NP post -cTBS) | *t* (degrees of freedom) | *p* | *p*(FDR)^a^ | *Cohen’s d* |
| --- | --- | --- | --- | --- | --- | --- |
| DLPFC | -0.27 ± 0.43 | -0.09 ± 0.43 | -2.27 (35) | 0.029 | 0.044* | -0.36 |
| PPC | -0.11 ± 0.89 | 0.004 ± 0.99 | -0.43 (35) | 0.674 | 0.674 | -0.07 |
| STC | -1.21 ± 0.75 | -0.81 ± 0.64 | -2.69 (35) | 0.011 | 0.033* | -0.45 |

^a^ Corrected using false discovery rate correction method; NP, natural processing, IH, inhibition, **p*(FDR) < 0.05.

**Table S16. Significant activation clusters within the combined ROI (DLPFC, PPC, VC) in Experiment 4: Analysis of stimulus exposure periods (a) 0-6 seconds, and (b) difference of post-3s vs. pre-3s period. (IH, inhibition)**

a.

| Region | Laterality | Cluster size  (Voxels) | *T* | *Z* | *p*(FDR) | Peak MNI coordinates | | |
| --- | --- | --- | --- | --- | --- | --- | --- | --- |
|  |  |  |  |  |  | x | y | z |
| **Pre-cTBS, Contrast: IH > NP** | | | | | | | | |
| **DLPFC** | L | 978 | 11.67 | 7.41 | < 0.001 | -50 | 10 | 30 |
|  | R | 872 | 9.49 | 6.63 | < 0.001 | 46 | 10 | 30 |
|  | R | 194 | 8.18 | 6.08 | < 0.001 | 10 | 26 | 36 |
|  | L | 260 | 5.68 | 4.75 | < 0.001 | -2 | 32 | 38 |
| **PPC** | L | 1083 | 11.83 | 7.46 | < 0.001 | -26 | -58 | 44 |
|  | R | 1085 | 9.23 | 6.53 | < 0.001 | 26 | -62 | 44 |
|  | L | 17 | 8.57 | 6.25 | < 0.001 | -26 | -70 | 34 |
|  | L | 13 | 3.63 | 3.32 | 0.001 | -38 | -56 | 20 |
|  | L | 10 | 2.75 | 2.60 | 0.011 | -12 | -72 | 36 |
|  | R | 14 | 2.42 | 2.31 | 0.023 | 4 | -42 | 52 |
| **STC** | L | 138 | 5.01 | 4.32 | < 0.001 | -60 | -54 | 8 |
|  | L | 37 | 4.54 | 4.00 | < 0.001 | -40 | -24 | 4 |
|  | L | 21 | 2.63 | 2.50 | 0.014 | -48 | 12 | -6 |
| **Pre-cTBS, Contrast: IH < NP** | | | | | | | | |
| **DLPFC** | R | 11 | 5.67 | 4.74 | < 0.001 | 12 | 54 | 42 |
| **PPC** | R | 23 | 5.42 | 4.59 | < 0.001 | 2 | -38 | 56 |
|  | L | 13 | 5.16 | 4.42 | < 0.001 | -2 | -34 | 50 |
|  | R | 30 | 5.02 | 4.33 | < 0.001 | 16 | -42 | 52 |
|  | R | 18 | 4.32 | 3.84 | 0.002 | 8 | -68 | 30 |
|  | L | 29 | 4.25 | 3.79 | 0.002 | -2 | -66 | 32 |
|  | R | 28 | 4.22 | 3.77 | 0.002 | 10 | -82 | 46 |
| **STC** | R | 427 | 11.88 | 7.47 | < 0.001 | 60 | -4 | 0 |
|  | L | 61 | 6.28 | 5.11 | < 0.001 | -64 | -12 | 4 |
|  | R | 10 | 3.62 | 3.31 | 0.009 | 46 | -58 | 14 |
| **Post-cTBS, Contrast: IH > NP** | | | | | | | | |
| **DLPFC** | L | 711 | 10.94 | 7.17 | < 0.001 | -44 | 12 | 30 |
|  | R | 498 | 9.03 | 6.44 | < 0.001 | 46 | 12 | 32 |
|  | R | 83 | 5.88 | 4.87 | < 0.001 | 10 | 26 | 36 |
|  | L | 63 | 5.13 | 4.40 | < 0.001 | -2 | 32 | 38 |
|  | L | 12 | 3.37 | 3.11 | 0.005 | -42 | 46 | 10 |
| **PPC** | L | 658 | 10.84 | 7.13 | < 0.001 | -24 | -56 | 44 |
|  | R | 530 | 8.01 | 6.00 | < 0.001 | 28 | -54 | 44 |
|  | L | 15 | 6.77 | 5.38 | < 0.001 | -26 | -70 | 36 |
| **STC** | L | 12 | 3.76 | 3.42 | 0.002 | -42 | -24 | 4 |
| **Post-cTBS, Contrast: IH < NP** | | | | | | | | |
| **STC** | R | 356 | 9.32 | 6.57 | < 0.001 | 66 | -16 | 4 |
|  | L | 32 | 6.11 | 5.00 | < 0.001 | -64 | -12 | 4 |

b.

| **Pre-cTBS, Contrast: IH > NP 0-3s** | | | | | | | | |
| --- | --- | --- | --- | --- | --- | --- | --- | --- |
| **DLPFC** | L | 784 | 10.29 | 6.94 | < 0.001 | -42 | 10 | 30 |
|  | R | 687 | 8.54 | 6.24 | < 0.001 | 48 | 10 | 30 |
|  | R | 131 | 7.14 | 5.57 | < 0.001 | 10 | 26 | 36 |
|  | L | 239 | 4.65 | 4.08 | < 0.001 | -8 | 52 | 30 |
| **PPC** | L | 860 | 11.56 | 7.37 | < 0.001 | -24 | -56 | 44 |
|  | R | 870 | 9.46 | 6.62 | < 0.001 | 28 | -54 | 44 |
|  | L | 17 | 7.91 | 5.95 | < 0.001 | -26 | -70 | 34 |
| **STC** | L | 34 | 3.42 | 3.15 | 0.003 | -60 | -56 | 18 |
|  | L | 18 | 2.86 | 2.69 | 0.011 | -56 | -52 | 10 |
| **Pre-cTBS, Contrast: IH < NP 0-3s** | | | | | | | | |
| **DLPFC** | R | 25 | 4.14 | 3.71 | 0.006 | 8 | 54 | 42 |
|  | L | 56 | 4.14 | 3.71 | 0.006 | -4 | 58 | 34 |
| **PPC** | L | 47 | 5.02 | 4.32 | 0.002 | -4 | -62 | 30 |
|  | R | 29 | 4.85 | 4.21 | 0.002 | 2 | -32 | 52 |
|  | R | 45 | 4.46 | 3.94 | 0.003 | 4 | -54 | 34 |
|  | L | 15 | 3.97 | 3.58 | 0.007 | -2 | -74 | 32 |
|  | L | 15 | 3.92 | 3.54 | 0.008 | -2 | -36 | 54 |
| **STC** | R | 309 | 7.85 | 5.92 | < 0.001 | 58 | 0 | -6 |
|  | L | 156 | 5.36 | 4.55 | 0.001 | -62 | -2 | 2 |
|  | R | 47 | 4.12 | 3.70 | 0.006 | 62 | -58 | 16 |
| **Pre-cTBS, Contrast: IH > NP 3-6s** | | | | | | | | |
| **DLPFC** | L | 594 | 11.15 | 7.24 | < 0.001 | -44 | 12 | 30 |
|  | R | 301 | 9.68 | 6.71 | < 0.001 | 46 | 12 | 32 |
|  | R | 48 | 5.30 | 4.51 | < 0.001 | 10 | 26 | 34 |
|  | R | 67 | 4.44 | 3.93 | < 0.001 | 46 | 32 | 22 |
|  | L | 40 | 3.96 | 3.58 | 0.001 | -8 | 26 | 36 |
| **PPC** | L | 540 | 11.34 | 7.30 | < 0.001 | -24 | -56 | 44 |
|  | R | 424 | 9.14 | 6.49 | < 0.001 | 28 | -54 | 44 |
|  | L | 15 | 6.97 | 5.48 | < 0.001 | -26 | -70 | 34 |
| **Pre-cTBS, Contrast: IH < NP 3-6s** | | | | | | | | |
| **DLPFC** | R | 22 | 6.03 | 4.96 | < 0.001 | 24 | 44 | 42 |
|  | L | 33 | 5.94 | 4.91 | < 0.001 | -22 | 42 | 42 |
|  | R | 60 | 5.00 | 4.32 | < 0.001 | 16 | 54 | 40 |
| **PPC** | R | 78 | 6.57 | 5.27 | < 0.001 | 18 | -42 | 50 |
|  | R | 217 | 6.13 | 5.02 | < 0.001 | 4 | -54 | 34 |
|  | L | 97 | 5.88 | 4.87 | < 0.001 | -2 | -72 | 30 |
|  | R | 51 | 5.74 | 4.78 | < 0.001 | 4 | -38 | 54 |
|  | L | 132 | 5.04 | 4.34 | < 0.001 | -2 | -38 | 56 |
| **STC** | R | 741 | 13.52 | Inf | < 0.001 | 64 | -20 | 0 |
|  | L | 453 | 12.74 | 7.73 | < 0.001 | -52 | -12 | 4 |
|  | R | 17 | 5.19 | 4.44 | < 0.001 | 54 | -60 | 12 |
| **Post-cTBS, Contrast: IH > NP 0-3s** | | | | | | | | |
| **DLPFC** | L | 719 | 10.59 | 7.05 | < 0.001 | -46 | 30 | 12 |
|  | R | 758 | 8.36 | 6.17 | < 0.001 | 48 | 16 | 28 |
|  | R | 175 | 6.94 | 5.46 | < 0.001 | 6 | 32 | 38 |
|  | L | 130 | 6.19 | 5.06 | < 0.001 | -2 | 32 | 38 |
|  | L | 10 | 3.26 | 3.03 | 0.004 | -42 | 42 | 10 |
| **PPC** | L | 1178 | 8.73 | 6.32 | < 0.001 | -20 | -70 | 56 |
|  | R | 1216 | 7.99 | 5.99 | < 0.001 | 32 | -64 | 46 |
|  | L | 17 | 6.00 | 4.94 | < 0.001 | -26 | -70 | 36 |
|  | R | 12 | 4.18 | 3.74 | < 0.001 | 22 | -68 | 36 |
| **STC** | L | 42 | 4.98 | 4.30 | < 0.001 | -56 | -42 | 4 |
|  | L | 62 | 4.86 | 4.22 | < 0.001 | -60 | -54 | 10 |
| **Post-cTBS, Contrast: IH< NP 0-3s** | | | | | | | | |
| **DLPFC** | R | 29 | 5.57 | 4.68 | 0.011 | 12 | 56 | 40 |
| **STC** | R | 40 | 5.06 | 4.35 | 0.011 | 64 | -12 | 4 |
| **Post-cTBS, Contrast: IH > NP 3-6s** | | | | | | | | |
| **DLPFC** | L | 573 | 8.26 | 6.11 | < 0.001 | -46 | 18 | 26 |
|  | R | 458 | 5.86 | 4.86 | < 0.001 | 42 | 14 | 34 |
|  | L | 54 | 5.04 | 4.34 | < 0.001 | -4 | 30 | 36 |
|  | R | 78 | 4.77 | 4.16 | < 0.001 | 10 | 26 | 34 |
| **PPC** | L | 672 | 6.44 | 5.20 | < 0.001 | -32 | -60 | 58 |
|  | R | 409 | 4.84 | 4.20 | < 0.001 | 32 | -58 | 50 |
|  | L | 10 | 3.64 | 3.33 | 0.003 | -26 | -70 | 36 |
| **Post-cTBS, Contrast: IH < NP 3-6s** | | | | | | | | |
| **DLPFC** | R | 37 | 5.83 | 4.84 | < 0.001 | 22 | 42 | 40 |
|  | R | 15 | 3.37 | 3.12 | 0.008 | 20 | 52 | 38 |
| **PPC** | R | 144 | 6.76 | 5.37 | < 0.001 | 4 | -56 | 34 |
|  | R | 10 | 5.01 | 4.32 | < 0.001 | 2 | -34 | 50 |
|  | L | 72 | 4.68 | 4.10 | < 0.001 | -4 | -76 | 30 |
| **STC** | R | 631 | 12.57 | 7.68 | < 0.001 | 64 | -20 | 0 |
|  | L | 320 | 10.77 | 7.11 | < 0.001 | -62 | -12 | 4 |
|  | R | 26 | 4.31 | 3.84 | 0.001 | 60 | -60 | 16 |
| **Contrast: Pre-cTBS (IH > NP) –Post-cTBS (IH > NP)** | | | | | | | | |
| **DLPFC** | L | 111 | 4.77 | 4.58 | 0.010 | -60 | 8 | 28 |
|  | R | 29 | 3.79 | 3.69 | 0.010 | 58 | 14 | 30 |
|  | R | 26 | 3.48 | 3.40 | 0.013 | 8 | 44 | 20 |
|  | L | 41 | 3.29 | 3.22 | 0.015 | -8 | 52 | 28 |
|  | R | 16 | 3.21 | 3.15 | 0.016 | 54 | 4 | 28 |
|  | L | 12 | 3.03 | 2.98 | 0.020 | -46 | 32 | 20 |
| **PPC** | R | 660 | 4.44 | 4.29 | 0.010 | 34 | -52 | 60 |
|  | L | 272 | 3.58 | 3.50 | 0.012 | -24 | -42 | 66 |
|  | R | 16 | 3.29 | 3.22 | 0.015 | 2 | -38 | 54 |
|  | L | 11 | 2.80 | 2.76 | 0.029 | -10 | -66 | 46 |
| **STC** | L | 20 | 3.39 | 3.32 | 0.014 | -56 | -62 | 14 |
| **Contrast: Pre-cTBS (IH < NP) –Post-cTBS (IH < NP)** | | | | | | | | |
| No clusters survived | | | | | | | | |
| **Contrast: Pre-cTBS [(IH < NP) 36 - (IH < NP) 03] –Post-cTBS [(IH < NP) 36 - (IH < NP) 03]** | | | | | | | | |
| **DLPFC** | L | 29 | 5.05 | 4.94 | 0.001 | -12 | 56 | 40 |
| **STC** | R | 117 | 5.74 | 5.58 | < 0.001 | 62 | 2 | 0 |

Data are thresholded at *p*(FDR) < 0.05, with a minimum cluster size of 10 voxels (For all analyses involving multiple comparisons, the *p*-values were further adjusted using an additional FDR correction). NP, natural processing, IH, inhibition, R: right. L: left.

**Table S17. Post-hoc beta values (*M ± SD*) for concentration vs. NP and inhibition vs. NP conditions for comparisons in the “Concentration” model of Experiment 3: Analysis of stimulus exposure periods (a) 0-6 seconds, and (b) difference of post-3s vs. pre-3s period.**

**a**

| Items | Concentration vs. NP | IH vs. NP | *t* (degrees of freedom) | *p* | *p*(FDR)^a^ | *Cohen’s d* |
| --- | --- | --- | --- | --- | --- | --- |
| DLPFC | 0.18 ± 0.41 | 0.48 ± 0.34 | -3.96 (14) | 0.001 | 0.003** | -1.02 |
| PPC | -0.18 ± 0.84 | 0.31 ± 0.64 | -3.26 (14) | 0.006 | 0.008** | -0.84 |
| STC | -0.08 ± 1.01 | -0.60 ± 0.56 | 3.11 (14) | 0.008 | 0.008** | 0.80 |

^a^ Corrected using false discovery rate correction method; NP, natural processing, IH, inhibition, ***p* < 0.01.

**b**

| Items | Post-3s(concentration vs. NP) - Pre-3s(concentration vs. NP) | Post-3s(IH vs. NP) -  Pre-3s(IH vs. NP) | *t* (degrees of freedom) | *p* | *p*(FDR)^a^ | *Cohen’s d* |
| --- | --- | --- | --- | --- | --- | --- |
| DLPFC | 0.33 ± 0.51 | -0.24 ± 0.49 | 2.38(14) | 0.032 | 0.048* | 0.62 |
| PPC | 0.14 ± 1.16 | -0.09 ± 0.85 | 0.46(14) | 0.652 | 0.652 | 0.12 |
| STC | 0.96 ± 1.17 | -1.24 ± 0.81 | 4.60(14) | 0.001 | 0.003** | 1.19 |

^a^ Corrected using false discovery rate correction method; NP, natural processing, IH, inhibition, **p*(FDR) < 0.05, ***p*(FDR) < 0.01.

**Table S18. Significant activation clusters within the combined ROI (DLPFC, PPC, STC) in the “Concentration model” of Experiment 3: Analysis of stimulus exposure periods (a) 0-6 seconds, and (b) difference of post-3s vs. pre-3s period.**

a.

| Region | Laterality | Cluster size  (Voxels) | *T* | *Z* | *p*(FDR) | Peak MNI coordinates | | |
| --- | --- | --- | --- | --- | --- | --- | --- | --- |
|  |  |  |  |  |  | x | y | z |
| **Contrast: concentration > NP** | | | | | | | | |
| **DLPFC** | L | 243 | 6.33 | 4.94 | 0.001 | −48 | 14 | 38 |
|  | L | 65 | 5.18 | 4.30 | 0.002 | -46 | 30 | 14 |
|  | R | 42 | 4.35 | 3.77 | 0.005 | 50 | 34 | 34 |
| **PPC** | L | 24 | 4.12 | 3.61 | 0.008 | -24 | -56 | 44 |
| **Contrast: concentration < NP** | | | | | | | | |
| No clusters survived | | | | | | | | |
| **Contrast: IH > NP** | | | | | | | | |
| **DLPFC** | L | 528 | 8.41 | 5.89 | < 0.001 | -42 | 10 | 32 |
|  | R | 22 | 5.73 | 4.62 | < 0.001 | 44 | 10 | 32 |
|  | R | 229 | 5.42 | 4.45 | < 0.001 | 50 | 34 | 34 |
|  | R | 53 | 5.15 | 4.29 | < 0.001 | 8 | 30 | 38 |
|  | R | 17 | 4.34 | 3.76 | 0.001 | 42 | 2 | 30 |
|  | L | 31 | 4.04 | 3.55 | 0.002 | -8 | 26 | 36 |
|  | R | 49 | 3.97 | 3.50 | 0.003 | 46 | 32 | 24 |
| **PPC** | L | 297 | 7.49 | 5.51 | < 0.001 | -26 | -56 | 46 |
|  | R | 208 | 6.05 | 4.80 | < 0.001 | 28 | -58 | 46 |
| **Contrast: IH < NP** | | | | | | | | |
| **STC** | R | 136 | 6.45 | 5.01 | 0.001 | 60 | -4 | 2 |
|  | R | 18 | 5.28 | 4.36 | 0.002 | 60 | -8 | 6 |
| **Contrast: IH > concentration** | | | | | | | | |
| **DLPFC** | R | 150 | 4.90 | 4.13 | 0.010 | 6 | 38 | 30 |
|  | L | 104 | 3.78 | 3.37 | 0.020 | -2 | 48 | 18 |
|  | L | 183 | 3.69 | 3.30 | 0.021 | -38 | 34 | 38 |
|  | L | 22 | 3.56 | 3.21 | 0.023 | -6 | 28 | 34 |
|  | R | 66 | 3.26 | 2.98 | 0.026 | 40 | 30 | 38 |
|  | L | 12 | 3.20 | 2.93 | 0.027 | -40 | 2 | 30 |
|  | R | 16 | 3.04 | 2.80 | 0.030 | 20 | 56 | 32 |
|  | R | 32 | 2.92 | 2.71 | 0.032 | 64 | 8 | 30 |
|  | L | 12 | 2.92 | 2.70 | 0.032 | -40 | 34 | 20 |
|  | L | 21 | 2.82 | 2.62 | 0.034 | -30 | 44 | 34 |
| **PPC** | L | 367 | 5.05 | 4.22 | 0.010 | -26 | -54 | 46 |
|  | R | 581 | 4.89 | 4.12 | 0.010 | 32 | -56 | 54 |
|  | R | 19 | 3.86 | 3.42 | 0.018 | 18 | -80 | 28 |
|  | L | 165 | 3.78 | 3.37 | 0.020 | -2 | -52 | 44 |
|  | R | 152 | 3.78 | 3.37 | 0.020 | 32 | -52 | 60 |
|  | R | 11 | 3.53 | 3.19 | 0.023 | 18 | -70 | 36 |
|  | L | 68 | 3.48 | 3.15 | 0.024 | -14 | -80 | 36 |
|  | R | 30 | 2.80 | 2.61 | 0.035 | 4 | -44 | 52 |
| **Contrast: IH < concentration** | | | | | | | | |
| **STC** | R | 184 | 8.36 | 5.87 | <0.001 | 64 | -4 | 0 |

Data are thresholded at *p*(FDR) < 0.05, with a minimum cluster size of 10 voxels (For all analyses involving multiple comparisons, the *p*-values were further adjusted using an additional FDR correction). NP, natural processing, IH, inhibition, R: right. L: left.

**b.**

| Region | Laterality | Cluster size  (Voxels) | *T* | *Z* | *p*(FDR) | Peak MNI coordinates | | |
| --- | --- | --- | --- | --- | --- | --- | --- | --- |
|  |  |  |  |  |  | x | y | z |
| **Interaction between *task condition* × *time*** | | | | | | | | |
| **DLPFC** | L | 135 | 21.91 | 5.47 | <0.001 | -10 | 40 | 20 |
|  | R | 11 | 12.51 | 4.14 | 0.001 | 44 | 2 | 30 |
|  | R | 51 | 10.36 | 3.73 | 0.004 | 46 | 24 | 42 |
|  | R | 51 | 8.67 | 3.37 | 0.011 | 4 | 34 | 36 |
|  | R | 26 | 7.27 | 3.03 | 0.025 | 46 | 36 | 14 |
| **PPC** | L | 78 | 12.03 | 4.05 | 0.002 | -24 | -56 | 48 |
|  | L | 21 | 8.53 | 3.34 | 0.012 | -24 | -42 | 68 |
| **STC** | R | 120 | 28.16 | 6.13 | < 0.001 | 52 | -14 | 6 |
|  | R | 178 | 24.55 | 5.77 | < 0.001 | 58 | -8 | 2 |
|  | L | 43 | 13.10 | 4.24 | 0.001 | -48 | -18 | 6 |
|  | L | 11 | 8.56 | 3.34 | 0.012 | -62 | -34 | 12 |
|  | L | 21 | 8.54 | 3.34 | 0.012 | -62 | -12 | 4 |
| **Contrast: Post 3s (concentration > NP) – Pre 3s (concentration > NP)** | | | | | | | | |
| **DLPFC** | L | 179 | 6.31 | 5.69 | < 0.001 | -10 | 40 | 20 |
|  | R | 139 | 4.20 | 3.99 | 0.004 | 12 | 48 | 34 |
|  | L | 20 | 4.08 | 3.88 | 0.005 | -34 | 22 | 38 |
|  | L | 12 | 3.59 | 3.45 | 0.014 | -42 | 38 | 22 |
|  | R | 77 | 3.42 | 3.30 | 0.019 | 46 | 28 | 38 |
|  | R | 37 | 3.27 | 3.16 | 0.026 | 44 | 38 | 14 |
| **PPC** | L | 64 | 3.59 | 3.45 | 0.014 | -24 | -44 | 72 |
|  | L | 33 | 3.59 | 3.45 | 0.014 | -42 | -68 | 44 |
|  | R | 22 | 3.22 | 3.11 | 0.028 | 38 | -70 | 52 |
|  | L | 53 | 3.16 | 3.06 | 0.031 | -10 | -48 | 54 |
| **STC** | R | 125 | 4.54 | 4.28 | 0.043 | 50 | -14 | 6 |
|  | L | 49 | 3.81 | 3.65 | 0.008 | -48 | -18 | 8 |
|  | R | 42 | 3.78 | 3.62 | 0.009 | 58 | -8 | 2 |
|  | L | 44 | 3.69 | 3.54 | 0.011 | -62 | -34 | 12 |
|  | R | 62 | 3.16 | 3.07 | 0.030 | 62 | -34 | 10 |
| **Contrast: Post 3s (concentration < NP) – Pre 3s (concentration < NP)** | | | | | | | | |
| No clusters survived | | | | | | | | |
| **Contrast: Post 3s (IH > NP) – Pre 3s (IH > NP)** | | | | | | | | |
| **DLPFC** | R | 11 | 4.99 | 4.65 | 0.010 | 44 | 2 | 30 |
| **PPC** | L | 36 | 4.83 | 4.52 | 0.010 | -24 | -56 | 48 |
|  | L | 15 | 4.42 | 4.18 | 0.013 | -20 | -60 | 54 |
| **Contrast: Post 3s (IH < NP) – Pre 3s (IH < NP)** | | | | | | | | |
| **DLPFC** | L | 149 | 5.91 | 5.39 | < 0.001 | -10 | 40 | 20 |
|  | R | 116 | 4.55 | 4.29 | 0.001 | 46 | 24 | 42 |
|  | R | 91 | 3.79 | 3.63 | 0.004 | 46 | 36 | 14 |
|  | R | 125 | 3.77 | 3.61 | 0.004 | 4 | 44 | 18 |
|  | L | 10 | 3.32 | 3.20 | 0.011 | -54 | 18 | 40 |
|  | L | 14 | 3.27 | 3.16 | 0.012 | -22 | 40 | 36 |
|  | L | 11 | 3.04 | 2.95 | 0.018 | -52 | 30 | 24 |
| **PPC** | L | 67 | 4.08 | 3.88 | 0.002 | -24 | -42 | 68 |
|  | R | 38 | 3.68 | 3.53 | 0.005 | 38 | -70 | 52 |
|  | L | 24 | 3.37 | 3.26 | 0.010 | -10 | -50 | 56 |
|  | R | 19 | 3.15 | 3.05 | 0.015 | 4 | -56 | 56 |
|  | R | 10 | 2.82 | 2.75 | 0.028 | 24 | -38 | 76 |
| **STC** | R | 458 | 7.41 | 6.48 | < 0.001 | 52 | -14 | 6 |
|  | L | 134 | 5.12 | 4.76 | < 0.001 | -48 | -18 | 6 |
|  | L | 55 | 3.95 | 3.77 | 0.003 | -62 | -34 | 12 |

Data are thresholded at *p*(FDR) < 0.05, with a minimum cluster size of 10 voxels (For all analyses involving multiple comparisons, the *p*-values were further adjusted using an additional FDR correction). NP, natural processing, IH, inhibition, R: right. L: left.

**Table S19. Post-hoc beta values (*M ± SD*) for inhibition vs. NP scramble and inhibition vs. NP normal conditions for comparisons in the “Scramble” model of Experiment 3: Analysis of stimulus exposure periods (a) 0-6 seconds, and (b) difference of post-3s vs. pre-3s period.**

**a**

| Items | IH vs. NP scramble | IH vs. NP normal | *t* (degrees of freedom) | *p* | *p*(FDR)^a^ | *Cohen’s d* |
| --- | --- | --- | --- | --- | --- | --- |
| DLPFC | 0.18 ± 0.27 | 0.48 ± 0.34 | -2.85 (14) | 0.013 | 0.039* | -0.74 |
| PPC | 0.33 ± 0.51 | 0.31 ± 0.64 | 0.12 (14) | 0.903 | 0.903 | 0.03 |
| STC | -0.40 ± 0.43 | -0.60 ± 0.56 | 1.04 (14) | 0.318 | 0.477 | 0.27 |

^a^ Corrected using false discovery rate correction method; NP, natural processing, IH, inhibition, **p*(FDR) < 0.05.

**b**

| Items | Post-3s(IH vs. NP scramble) -  Pre-3s(IH vs. NP scramble) | Post-3s(IH vs. NP normal) -  Pre-3s(IH vs. NP normal) | *t* (degrees of freedom) | *p* | *p*(FDR)^a^ | *Cohen’s d* |
| --- | --- | --- | --- | --- | --- | --- |
| DLPFC | -0.01 ± 0.31 | -0.24 ± 0.49 | 2.22 (14) | 0.044 | 0.066 | 0.57 |
| PPC | -0.38 ± 0.60 | -0.09 ± 0.85 | -1.10 (14) | 0.291 | 0.291 | -0.28 |
| STC | -0.60 ± 0.51 | -1.24 ± 0.81 | 2.35 (14) | 0.034 | 0.066 | 0.61 |

^a^ Corrected using false discovery rate correction method. NP, natural processing, IH, inhibition,

**Table S20. Significant activation clusters within the combined ROI (DLPFC, PPC, STC) in the “Scramble model” of Experiment 3: Analysis of stimulus exposure periods (a) 0-6 seconds, and (b) difference of post-3s vs. pre-3s period. (IH,inhibition)**

**a.**

| Region | Laterality | Cluster size  (Voxels) | *T* | *Z* | *p*(FDR) | Peak MNI coordinates | | |
| --- | --- | --- | --- | --- | --- | --- | --- | --- |
|  |  |  |  |  |  | x | y | z |
| **Contrast: IH > NP normal** | | | | | | | | |
| **DLPFC** | L | 544 | 11.20 | Inf | < 0.001 | −48 | 10 | 34 |
|  | R | 244 | 6.85 | 5.81 | < 0.001 | 46 | 20 | 34 |
|  | R | 100 | 6.83 | 5.80 | < 0.001 | 10 | 26 | 36 |
|  | R | 78 | 6.57 | 5.64 | < 0.001 | 46 | 30 | 24 |
|  | L | 49 | 4.86 | 4.42 | < 0.001 | -8 | 26 | 36 |
|  | R | 18 | 4.72 | 4.31 | <0.001 | 50 | 10 | 30 |
| **PPC** | L | 327 | 10.09 | 7.58 | < 0.001 | -26 | -56 | 46 |
|  | R | 239 | 8.50 | 6.78 | < 0.001 | 28 | -60 | 44 |
|  | R | 18 | 3.60 | 3.40 | 0.003 | 26 | -64 | 64 |
| **Contrast: IH < NP normal** | | | | | | | | |
| **STC** | R | 240 | 8.25 | 6.65 | < 0.001 | 62 | -4 | 2 |
| **Contrast: IH > NP scramble** | | | | | | | | |
| **DLPFC** | L | 20 | 6.05 | 5.28 | < 0.001 | -12 | 48 | 28 |
|  | R | 184 | 5.75 | 5.08 | < 0.001 | 8 | 40 | 26 |
|  | L | 51 | 5.41 | 4.83 | < 0.001 | -16 | 52 | 36 |
|  | L | 67 | 5.16 | 4.65 | < 0.001 | -2 | 40 | 30 |
|  | L | 33 | 5.14 | 4.64 | < 0.001 | -20 | 40 | 20 |
|  | R | 83 | 4.84 | 4.41 | 0.001 | 40 | 28 | 42 |
|  | L | 23 | 3.92 | 3.67 | 0.004 | -44 | 30 | 38 |
|  | R | 27 | 3.67 | 3.46 | 0.007 | 64 | 8 | 26 |
|  | L | 25 | 3.29 | 3.13 | 0.014 | -38 | 4 | 38 |
| **PPC** | L | 346 | 5.32 | 4.77 | < 0.001 | -36 | -58 | 50 |
|  | R | 303 | 4.74 | 4.33 | 0.001 | 30 | -58 | 50 |
|  | R | 26 | 4.11 | 3.83 | 0.003 | 24 | -40 | 76 |
|  | R | 16 | 3.90 | 3.66 | 0.004 | 14 | -42 | 78 |
|  | R | 27 | 3.67 | 3.46 | 0.007 | 32 | -50 | 60 |
|  | R | 11 | 3.34 | 3.18 | 0.012 | 10 | -68 | 46 |
|  | L | 32 | 3.24 | 3.09 | 0.015 | -12 | -62 | 46 |
| **Contrast: IH < NP scramble** | | | | | | | | |
| **STC** | R | 183 | 9.34 | 7.22 | < 0.001 | 64 | -4 | 0 |
|  | L | 22 | 4.92 | 4.47 | < 0.001 | -64 | -12 | 4 |
| **Contrast:0-6 (IH-NP normal) > (IH-NP scramble)** | | | | | | | | |
| **DLPFC** | L | 461 | 8.35 | 6.70 | < 0.001 | -44 | 10 | 32 |
|  | R | 33 | 5.16 | 4.64 | < 0.001 | 46 | 20 | 34 |
|  | R | 38 | 4.41 | 4.07 | 0.001 | 46 | 30 | 24 |
|  | R | 50 | 4.36 | 4.03 | 0.001 | 50 | 32 | 34 |
| **PPC** | L | 79 | 5.03 | 4.55 | < 0.001 | -24 | -56 | 46 |
| **Contrast:0-6 (IH-NP normal) < (IH-NP scramble)** | | | | | | | | |
| **DLPFC** | L | 22 | 4.20 | 3.90 | 0.016 | -16 | 52 | 36 |
| **PPC** | L | 41 | 4.86 | 4.42 | 0.006 | -40 | -72 | 44 |
|  | R | 15 | 4.65 | 4.26 | 0.009 | 44 | -68 | 44 |
|  | L | 10 | 3.83 | 3.59 | 0.024 | -2 | -72 | 36 |
|  | R | 13 | 3.68 | 3.46 | 0.033 | 4 | -74 | 34 |

Data are thresholded at *p*(FDR) < 0.05, with a minimum cluster size of 10 voxels (For all analyses involving multiple comparisons, the *p*-values were further adjusted using an additional FDR correction). NP, natural processing, IH, inhibition, R: right. L: left.

**b.**

| Region | Laterality | Cluster size  (Voxels) | *T* | *Z* | *p*(FDR) | Peak MNI coordinates | | |
| --- | --- | --- | --- | --- | --- | --- | --- | --- |
|  |  |  |  |  |  | x | y | z |
| **Interaction between *task condition* × *time* × *voice type*** | | | | | | | | |
| **DLPFC** | L | 76 | 22.99 | 4.42 | 0.018 | -2 | 36 | 36 |
|  | R | 10 | 13.64 | 3.39 | 0.046 | 4 | 36 | 38 |
| **Contrast: Post 3s (IH > NP normal) – Pre 3s (IH > NP normal)** | | | | | | | | |
| **PPC** | L | 63 | 5.04 | 4.77 | 0.003 | -24 | -56 | 48 |
|  | R | 10 | 3.79 | 3.67 | 0.022 | 22 | -62 | 46 |
| **Contrast: Post 3s (IH < NP normal) – Pre 3s (IH < NP normal)** | | | | | | | | |
| **DLPFC** | L | 161 | 5.27 | 4.97 | < 0.001 | -10 | 40 | 20 |
|  | R | 138 | 4.90 | 4.65 | < 0.001 | 46 | 24 | 42 |
|  | R | 99 | 4.57 | 4.37 | < 0.001 | 46 | 38 | 14 |
|  | R | 179 | 4.07 | 3.92 | 0.001 | 12 | 48 | 34 |
|  | L | 47 | 3.96 | 3.82 | 0.002 | -44 | 40 | 22 |
|  | L | 15 | 3.88 | 3.75 | 0.002 | -54 | 18 | 40 |
|  | L | 19 | 3.74 | 3.62 | 0.003 | -22 | 40 | 36 |
|  | R | 10 | 3.63 | 3.53 | 0.004 | 20 | 40 | 38 |
|  | L | 12 | 3.38 | 3.29 | 0.008 | -48 | 18 | 40 |
| **PPC** | L | 77 | 4.50 | 4.30 | < 0.001 | -22 | -40 | 74 |
|  | R | 56 | 4.21 | 4.05 | 0.001 | 38 | -70 | 52 |
|  | L | 53 | 3.86 | 3.74 | 0.003 | -42 | -62 | 52 |
|  | L | 49 | 3.36 | 3.27 | 0.008 | -8 | -50 | 58 |
|  | R | 38 | 3.11 | 3.04 | 0.014 | 4 | -54 | 58 |
|  | R | 11 | 3.11 | 3.03 | 0.015 | 24 | -38 | 76 |
| **STC** | R | 423 | 7.03 | 6.38 | < 0.001 | 52 | -14 | 6 |
|  | L | 113 | 4.12 | 3.97 | 0.001 | -50 | -14 | 6 |
|  | L | 23 | 3.42 | 3.33 | 0.007 | -62 | -34 | 12 |
| **Contrast: Post 3s (IH > NP scramble) – Pre 3s (IH > NP scramble)** | | | | | | | | |
| No clusters survived | | | | | | | | |
| **Contrast: Post 3s (IH < NP scramble) – Pre 3s (IH < NP scramble)** | | | | | | | | |
| **PPC** | R | 204 | 4.27 | 4.10 | 0.003 | 4 | -62 | 44 |
|  | R | 19 | 4.08 | 3.93 | 0.004 | 4 | -76 | 30 |
|  | L | 27 | 3.96 | 3.82 | 0.005 | -40 | -60 | 50 |
|  | L | 99 | 3.95 | 3.82 | 0.005 | -2 | -62 | 44 |
|  | R | 18 | 3.27 | 3.19 | 0.023 | 40 | -60 | 58 |
|  | L | 43 | 3.21 | 3.13 | 0.026 | -14 | -36 | 52 |
|  | L | 10 | 3.19 | 3.11 | 0.027 | -14 | -48 | 42 |
|  | L | 18 | 3.15 | 3.08 | 0.029 | -8 | -78 | 36 |
| **STC** | R | 145 | 5.59 | 5.24 | 0.001 | 60 | -4 | 0 |
|  | R | 54 | 5.15 | 4.87 | 0.001 | 56 | -12 | 6 |
|  | L | 17 | 3.30 | 3.22 | 0.022 | -58 | -12 | 2 |
|  | L | 11 | 3.23 | 3.16 | 0.025 | -50 | -14 | 4 |

Data are thresholded at *p*(FDR) < 0.05, with a minimum cluster size of 10 voxels (For all analyses involving multiple comparisons, the *p*-values were further adjusted using an additional FDR correction). NP, natural processing, IH, inhibition, R: right. L: left.

**Table S****21. Demographic information of participants in the final analysis (*M ± SD*).**

| Experiment 1 | Auditory (n = 51) | Visual (n = 77) |  |
| --- | --- | --- | --- |
| Age (years) | 21 ± 2.5 | 20.73 ± 2.1 |  |
| Sex (male/female) | 30/21 | 38/39 |  |
| Experiment 2 | Exp 2.1  (n = 30) | Exp 2.2  (n = 40) | Exp 2.3  (n = 38) |
| Age (years) | 20.27 ± 1.39 | 20.38 ± 1.7 | 21.55 ± 2.46 |
| Sex (male/female) | 15/15 | 23/17 | 19/19 |
| Experiment 3 | Auditory (n = 15, revisited participants from Experiment 1) | Visual (n = 27, revisited participants from Experiment 1) |  |
| Age (years) | 21.4 ± 2.13 | 21.04 ± 2.46 | 2 |
| Sex (male/female) | 8/7 | 14/13 |  |
| Experiment 4 | Auditory (total n = 36) |  |  |
| Age (years) | 20.34 ± 1.7 |  |  |
| Sex (male/female) | 19/17 |  |  |

**Reference**

1. B. D. Lester, S. P. Vecera, Active Listening Delays Attentional Disengagement and Saccadic Eye Movements. *Psychon Bull Rev* **25**, 1021–1027 (2018).

2. N. Kreddig, M. I. Hasenbring, E. Keogh, Comparing the Effects of Thought Suppression and Focused Distraction on Pain-Related Attentional Biases in Men and Women. *The Journal of Pain* **23**, 1958–1972 (2022).

3. Z. He, Y. Lin, L. Xia, Z. Liu, D. Zhang, R. Elliott, Critical role of the right VLPFC in emotional regulation of social exclusion: a tDCS study. *Social Cognitive and Affective Neuroscience* **13**, 357–366 (2018).
